# Conservative interventions and clinical outcome measures used in the perioperative rehabilitation of breast cancer patients undergoing mastectomy: a scoping review

**DOI:** 10.1186/s12905-022-01927-3

**Published:** 2022-08-16

**Authors:** Janny Mathieu, Catherine Daneau, Nadège Lemeunier, Annabelle Doyon, Andrée-Anne Marchand, Martin Descarreaux

**Affiliations:** 1 Department of Anatomy, Université du Québec à Trois-Rivières, Trois-Rivières, QC Canada; 2 UMR1295, Toulouse III University, Inserm, Equipe EQUITY, Equipe constitutive du CERPOP, Toulouse, France; 3 Faculty of Health Sciences, Ontario Tech University, Oshawa, ON Canada; 4 Department of General Surgery, Centre Intégré Universitaire de Santé et de Services Sociaux de la Mauricie-et-du-Centre-du-Québec, Trois-Rivières, QC Canada; 5 Chiropractic Department, Université du Québec à Trois-Rivières, Trois-Rivières, QC Canada; 6 Department of Human Kinetics, Université du Québec à Trois-Rivières, 3351, boul. des Forges, C.P. 500, Trois-Rivières, QCQC G8Z 4M3 Canada

**Keywords:** Breast neoplasms, Exercise, Mastectomy, Physical disability, Rehabilitation

## Abstract

**Background:**

Mastectomy is the first-line treatment approach for more than 90% of breast cancer patients. The numerous physical impairments associated with this surgical procedure negatively impact the patient’s quality of life. To date, rehabilitation resources available for breast cancer patients undergoing mastectomy within the institutions affiliated to the Centre intégré universitaire de soins de santé et de services sociaux de la Mauricie-et-du-Centre-du-Québec (CIUSSS-MCQ) are lacking and do not always seem to reflect the particularities of breast cancer care pathways. The purpose of this review was to identify and describe the conservative interventions and the clinical outcome measures used in the perioperative physical rehabilitation of women with breast cancer who are awaiting or have undergone mastectomy. We also aimed to report on the barriers and facilitators to study participation and completion.

**Methods:**

MEDLINE, CINAHL, and the Cochrane Library were searched from inception to January 2021, and we updated the search on July 11, 2022. We included peer-reviewed English and French literature with quantitative designs, describing conservative interventions and clinical outcome measures used within rehabilitation programs designed for women who were awaiting or had undergone mastectomy. Paired reviewers independently reviewed all citations and articles using a two-phase screening process and independently extracted the data.

**Results:**

Of the 6080 articles identified, 57 met the inclusion criteria. Most interventions were multimodal, which combined exercise with patient education, manual therapy, and/or lymphatic drainage. The most frequently used objective measures of physical function were shoulder range of motion, muscle strength, and signs of lymphedema. In contrast, the primary patient-reported outcome measures were quality of life, shoulder function, and pain. Undergoing another breast surgery, death, and cancer recurrence were the most reported barriers to study completion.

**Conclusion:**

This scoping review reports on the heterogeneity and wide range of conservative interventions and clinical outcome measures used in the physical rehabilitation of breast cancer patients who had undergone or were scheduled to undergo mastectomy. Tailoring interventions to breast cancer patients’ needs and promoting outpatient rehabilitation interventions appear to be better suited to the particularities of breast cancer care pathways. Further research is needed to better identify barriers and facilitators to study participation and completion.

**Supplementary Information:**

The online version contains supplementary material available at 10.1186/s12905-022-01927-3.

## Background

Breast cancer is a malignant tumor with the second highest incidence rate among females worldwide [1]. In 2020, breast cancer cases accounted for one in four new cancer diagnoses among Canadian women [1]. Implementation of a biennial population-based mammography screening program in 1998 [2], along with the improvement of surgical techniques [3] has fortunately contributed to a significant decrease in breast cancer mortality rates in Canada over the last twenty years [4]. Specifically in Quebec, over 90% of breast cancer patients in the early 2000s were diagnosed with an in situ breast tumor (stage 0) or a stage I or II disease [5]. Early detection of lower histological grade cancer significantly improves breast cancer patients’ prognosis, allowing treatment strategies to be initiated sooner, thus reducing the risk of disease progression [5]. In 2003, patients diagnosed with a stage I or II breast tumor showed a 5-year survival rate of 98.1% and 89.2%, respectively, while this number dropped to 10.5% for patients with a stage IV disease [5]. Although breast cancer patients may now benefit from a longer life expectancy, this is not without consequences for those women, who will still need to undergo a series of therapeutic interventions whose physical, psychological, and socio-economic effects are substantial [6].

Mastectomy (i.e., surgery to remove part of or all the breast) represents the first-line treatment approach for more than 90% of breast cancer patients [7]. Physical impairments associated with this surgical procedure are numerous (e.g., loss of shoulder range of motion (ROM), pain, lymphedema, and muscle weakness) [8], leading to limitations in activities of daily living, which negatively impacts the patient’s quality of life [9, 10]. Several studies aimed to develop effective interventions to support breast cancer patients dealing with musculoskeletal adverse events (AEs) resulting from a mastectomy. A systematic review published in 2015 by De Groef et al. [11] confirmed the effectiveness of multimodal physical therapy (i.e., stretching exercises combined with general active exercises) to treat upper limb impairments after breast cancer treatments. Another systematic review published in 2019 by Ribeiro et al. [12] concluded that ROM and upper extremity strengthening exercises effectively improve shoulder ROM in patients who had undergone breast surgery. However, when comparing the 15 randomized controlled studies included in this review, the rehabilitation interventions described were found to be highly heterogeneous [12]. Although there seems to be no consensus as to which parameters should be chosen to promote optimal postoperative recovery for breast cancer patients, the use of self-management strategies in cancer patients is widely emphasized in the literature for its perceived benefits on patients’ quality of life and ability to manage treatment-related symptoms, besides promoting better utilization of health care and services [13, 14].

To date, there are limited rehabilitation resources available for breast cancer patients undergoing mastectomy within the institutions of the Centre intégré universitaire de santé et de services sociaux de la Mauricie-et-du-Centre du Québec (CIUSSS-MCQ). Previously published systematic reviews certainly provide important insights regarding the rehabilitation of women who have undergone mastectomy for breast cancer, but these have focused primarily on interventions initiated in the early postoperative period, and targeted specific outcome measures. Consequently, to ensure that we will provide timely and comprehensive patient care for women undergoing mastectomy, we must first establish a more comprehensive portrait of perioperative rehabilitation interventions and current clinical outcome measures. It stands to reason that such understanding represents a prerequisite for developing interventions whose modalities will reflect patients’ needs and expectations and consider the particularities of breast cancer care pathways.

Therefore, this study aimed to identify the conservative interventions and the clinical outcome measures used as part of the perioperative physical rehabilitation of women diagnosed with breast cancer who plan to or have undergone mastectomy. As a secondary objective, we aimed to report on the barriers and facilitators to participating and completing these rehabilitation programs.

## Methods

### Study design

To address our broad research question, a scoping review was conducted based on the framework from Arksey and O’Malley [15] and Levac et al. [16]. This type of study allows us to report on the current state of knowledge in a research field and captures the breadth of information on a topic that has been widely studied and for which the available data are numerous and heterogeneous [17]. Consistent with this framework, we did not appraise the methodological quality of the included studies.

### Identifying the research question

Our scoping review was guided by the following research question: *What are the conservative interventions and clinical outcome measures used as part of the perioperative physical rehabilitation of women diagnosed with stage 0-III breast cancer who are awaiting or have undergone a mastectomy?*

### Identifying relevant studies

#### Data sources and searches

Our search strategy was developed by one of the authors (J.M.) and two coauthors (A.A.M., M.D.) subsequently cross-validated the search to ensure completeness of results. The search strategy was first developed in MEDLINE and then adapted to other bibliographic databases. Search terms included controlled vocabulary for each database and free text words for the key concepts of breast cancer, mastectomy, and rehabilitation (see Additional file 1 for full search strategy). In addition, reference lists from relevant articles and previously published systematic reviews were hand searched for any additional relevant studies. We initially searched MEDLINE, CINAHL, and Cochrane databases from inception to January 24, 2021, and updated the search on July 11, 2022. EndNote X9 was used to de-duplicate references electronically across all databases.

### Study selection

#### Eligibility criteria

To be included, studies had to meet the following criteria: (1) be written in the English or French language; (2) were randomized controlled trials, quasi-randomized trials, cohort studies, secondary analysis, exploratory studies or systematic reviews (for reference purposes only); (3) focused on adult women (aged ≥ 18 years) who engaged in a physical rehabilitation intervention before or following any type of mastectomy (e.g., partial mastectomy or breast conserving surgery (BCS), lumpectomy, quadrantectomy, wide local excision, segmental mastectomy) for a stage 0-III breast cancer. Studies including participants that underwent a mastectomy combined with an axillary staging procedure (i.e., axillary sampling or sentinel lymph node biopsy) or a lymph node dissection (ALND) were also included, considering that these surgical interventions are in line with the Society of Surgical Oncology-American Society of Clinical Oncology (SSO-ASTRO) clinical practice guideline recommendations [18]. All included studies also had to match the following characteristics for physical rehabilitation interventions:

1. Initiated within 3 months preceding or following the surgical intervention.
2. Involved at least one active physical modality (i.e., the patient physically contributed to its own treatment), including but not limited to exercises, conditioning, yoga, Taiichi, and Pilates.
3. Provided alone or in combination with other types of conservative interventions (e.g., patient education, manual therapy, manual lymphatic drainage (MLD), nutritional or psychological interventions).

Study exclusion criteria included: cross-sectional studies, case report and case series designs, study protocol, practice guidelines, letters, editorials, commentaries, unpublished manuscripts, books and book chapters, conference proceedings, cost analyses, meeting and conference abstracts, thesis and dissertations, non-systematic reviews, qualitative studies, laboratory studies and cadaveric or animal studies. Studies focusing on breast cancer survivors (i.e., patients who had completed all forms of cancer treatments), on patients with a stage IV disease, on managing or preventing the AEs of systemic treatments (i.e., chemotherapy, radiation, or hormonal therapy) rather than surgery, and studies who failed to provide enough methodological details (i.e., minimally a description of the intervention’s procedures and its initiation time) to enable interventions’ replication were also excluded.

#### Screening and agreement

A two-phase screening process was used to select eligible studies. In phase I screening, a pair of independent reviewers (J.M., C.D.) screened citation titles and abstracts to determine the eligibility of studies (categorizing studies as possibly relevant or irrelevant). In instances where eligibility could not be ensured due to limited information in the title/abstract, the citation was considered “possibly relevant’’ until a final decision could be made upon full text review. A pair of independent reviewers (J.M., N.L.) screened possibly relevant studies in full text during phase II screening to determine eligibility and reasons for exclusion were documented. Reviewers met to discuss disagreements and to reach consensus in both phases. An additional reviewer (A.A.M.) was involved if consensus could not be reached.

### Data charting

Both reviewers (J.M., N.L.) extracted the following data (when available) from half of the eligible studies: (1) study description (first author, publication year and country of origin); (2) study population (sample size, cancer stage, surgery type and systemic treatment administered); (3) rehabilitation interventions provided (e.g., type, initiation, duration, frequency); (4) outcome measures and outcome validation information and (5) patients’ experience data (e.g., reasons for not completing the study or for declining to participate, adherence outcomes, postoperative complications, AEs). An evidence table was built (see Additional file 2: Table S1) using a Microsoft Word document. A third reviewer (M.D.) independently verified the extracted data to minimize error.

### Data synthesis and analysis

A descriptive synthesis was conducted to provide details regarding the total number of studies kept for analysis, their authors and year of publication, country where they were conducted, study design, and study population. The summary of evidence table includes a brief description of conservative rehabilitation interventions identified as well as outcome measures used for each of them. Interventions’ procedures and data on barriers and facilitators to engagement in these interventions were summarized separately in Additional file 2: Tables S1 and S2. To answer our research question, our review findings were sorted by themes of interest: “conservative rehabilitation interventions,” “clinical outcome measures,” and “patients’ experience.”

## Results

### Descriptive synthesis

**Fig. 1:**
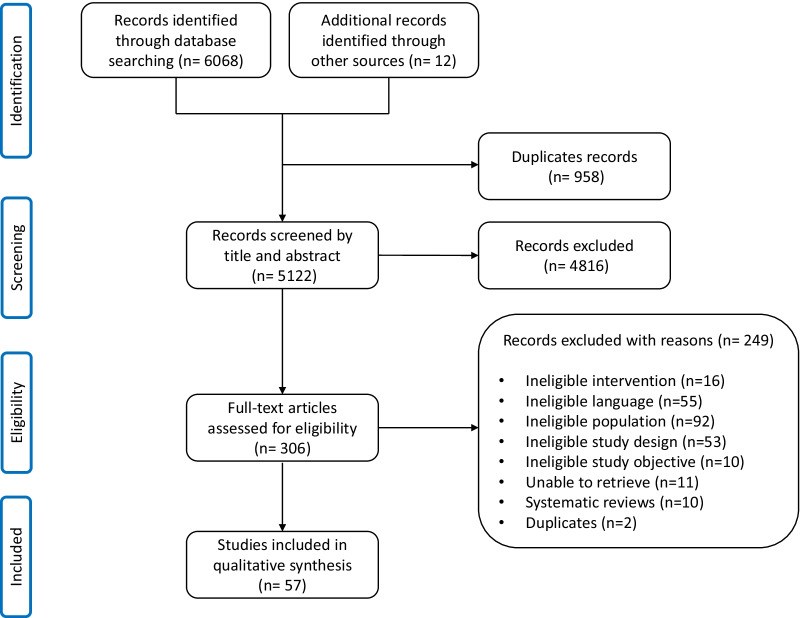
Flowchart

A total of 6068 articles were identified from the literature search, and twelve articles were retrieved from additional data sources. Following the removal of duplicates (n = 958), 5065 articles were excluded (see Fig. 1), bringing the total count to 57 papers, including 54 original studies.

Table 1 summarizes the key findings from the included articles. Most studies (39 of 57) were RCT [19–57], five were controlled non randomized clinical trials [58–63], four were prospective cohort studies [61, 64–66], one was a retrospective cohort study [67], two was a case–control study [68, 69], four were quasi-experimental [70–73], one was a pilot study [74] and one was a feasibility study [75]. The studies originated from 22 countries distributed across 4 continents (i.e., Americas, Europe, Asia and Oceania), most of which were high income countries [19–26, 29, 31, 33–36, 39–41, 44–47, 50, 52–54, 56, 57, 59, 61–69, 71, 73–75] or upper-middle income countries [27, 28, 30, 32, 37, 38, 42, 43, 48, 49, 58, 60, 72], with only three studies conducted in lower-middle income countries [51, 55, 70]. The body of literature on this topic turned out to be quite recent, with 71.9% (41 of 57) of studies published between 2010 and 2022 and 22.8% (13 out of 57) between 2000 and 2009.

**Table 1:** Summary table of evidence

First author (year) Country	Study design	Participants	Intervention	Outcome measures
Ammitzbøll [1] (2019) Denmark	RCT	N = 158 **Exercise IG; n = 82** *Age, mean* ± *SD*: 53 ± 10 *Stage, n (%):* I: 12 (15)/II: 48 (59)/III: 15 (18)/N/A: 7 (9) *Sx type, n (%):* LUMP + ALDN: 43(52)/MX + ALDN:39(48) *Systemic treatment, n (%)* RT: 82 (100)/Adj Ch: 48 (59) Neoadj Ch: 25 (30)/HT: 64 (78) **Usual-care CG; n = 76** *Stage, n (%):* I: 16 (21)/II: 35 (46)/III: 18 (24)/N/A: 7 (9) *Sx type, n (%):* LUMP + ALDN: 41(54) MX + ALDN: 35(46) *Systemic treatment, n (%)* RT: 82 (100)/Adj Ch: 45 (59) Neoadj Ch: 21 (28)/HT: 51 (67)	**Exercise intervention group** Resistance exercises program (covered all major muscles groups of the UL and lower limbs, and core strength and stability) *Phase* 1 (w1-w20) Initiation: 3rd post-op w Frequency: 3 days/w *Phase 2 (w21-w50)* Initiation: after phase 1 Frequency: 3 days/w *Exercise sessions duration*: 50–55 min (10–15-min warm-up, 40 min of resistance training) **Usual-care control group** No intervention provided but were allowed to participate in municipality-led rehabilitation programs without restrictions	Arm VOL*-ILVD* (water displacement) LE-related symptoms: heaviness, tightness and swelling (NRS-11) Muscle strength (7RM-test and dynamometer) Shoulder movement (goniometer) Interlimb mass difference-*ILMD* (DXA and arm scan) Clinical examinationLE (Stanton & al. criteria) Clinically relevant LE (> 3% increased ILVD, NRS-11 ≥ 2 and 2 or more clinical criteria)
Ammitzbøll [2] (2019) Denmark	RCT	N = 158 **Exercise IG; n = 82** *Age, mean* ± *SD*: 53 ± 10 *Stage, n (%):* I: 12 (15)/II: 48 (59)/III: 15 (18) N/A: 7 (9) *Sx type, n (%):* LUMP + ALDN: 43(52)/MX + ALDN:39(48) *Systemic treatment, n (%)* RT: 82 (100)/Adj Ch: 48 (59) Neoadj Ch: 25 (30)/HT: 64 (78) **Usual-care CG; n = 76** *Stage, n (%):* I: 16 (21)/II: 35 (46)/III: 18 (24)/N/A: 7 (9) *Sx type, n (%):* LUMP + ALDN: 41(54)/MX + ALDN: 35(46) *Systemic treatment, n (%)* RT: 82 (100)/Adj Ch: 45 (59) Neoadj Ch: 21 (28)/HT: 51 (67)	**Exercise intervention group** Resistance exercises program (covered all major muscles groups of the UL and lower limbs, and core strength and stability) Phase 1 (w1-w20) Initiation: 3rd post-op w Frequency: 3 days/w *Phase 2 (w21-w50)* Initiation: after phase 1 Frequency: 3 days/w Exercise sessions duration: 50–55 min (10–15-min warm-up, 40 min of resistance training) **Usual-care control group** No intervention provided but were allowed to participate in municipality-led rehabilitation programs without restrictions	HRQOL (EORTC QLQ C-30 v3; FACIT-f) Symptom clustered: pain-sleep-fatigue (EORTC QLQ-C30 v3)
Anderson [3] (2012) USA	RCT	N = 104 **Intervention arm; n = 52** *Age group, n (%):* < *50:* 21 (40)/*50–64:* 23 (44) *65–74:* 4 (8)/ > *75:* 4 (8) *Stage, n (%):* I: 25 (48)/II: 19 (37)/III: 8 (15)/N/A: 1(2) *Sx type, n (%):* LUMP: 23 (44)/MX: 28 (54)/N/A: 1 (2) *Type of node dissection, n (%):* SND only: 10 (19)/AND: 39 (75) Neither: 1 (2)/N/A: 2 (4) *Systemic treatment, n (%):* *Ch:* 31(60)/*HT:* 26(50)/*RT:* 31(60) **Comparison arm; n = 52** *Age group, n (%)* < *50:* 23 (44)/*50–64:* 19 (37) *65–74:* 7 (13)/ > *75:* 3 (6) *Stage, n (%):* I: 26 (50)/II: 21 (40)/III: 4 (8)/N/A: 1(2) *Sx type, n (%):* LUMP: 25 (48)/MX: 24 (46)/N/A: 3 (6) *Type of node dissection, n (%):* SND only: 9 (17)/ALND: 40 (77) Neither: 0/N/A: 3 (6) *Systemic treatment, n (%):* Ch: 31(60)/HT: 23(44)/RT: 36(69)	**Intervention arm** Comprehensive program consisting of tailored exercises and LE prevention module *Initiation*: 4-12w post-op *Intensive phase (m1-3)* Frequency: 2 days/w *Phase 2 (m4-6):* Transition to HB exercises (Supervised exercises 1 day/w) *Phase 3 (m7-12):* HB exercises *Exercise sessions duration*: 65 min (5-min warm-up, 30-min of walking, 20 min of strengthening exercises and 10 min of stretching) **Comparison arm** Usual care consisting of patient ED (LE awareness, tips about PA and nutrition, recommendations for improving function and strength)	Arm VOL (Water displacement) Function (6MWT) HRQOL (FACT-B)
Bendz [4] (2002) Sweden	RCT	N = 230 **Group A; n = 115** *Age, mean* ± *SD*: 58 ± 11 *Stage*: N/A *Sx type, n (%):* MX: 31 (31)/MX + RT: 5 (5) QT: 20 (20)/QT + RT: 45 (44) **Group B; n = 115** *Age, mean* ± *SD*: 58 ± 11 *Stage:* N/A *Sx type, n (%):* MX: 22 (21)/MX + RT: 7 (7) QT: 23 (22)/QT + RT: 52 (50)	**Group A** Early shoulder exercises (to be started on the 1st pod) *Day 1–13:* Early shoulder exercises including intermittent hand contractions and basic ROM exercises/*From Day 14*: Comprehensive ROM exercise program **Group B; n = 115** Delayed shoulder exercises *Day 1–13:* No further information was provided/*From Day 14:* Comprehensive ROM exercise program Frequency: 3 times daily 5 times in every set	Arm VOL (water displacement) Shoulder ROM (Myrin goniometer) Grip strength (vigorimeter) Patient-reported measures of pain, heaviness and tension (VAS scale)
Beurskens [5] (2007) Netherlands	RCT	N = 30 **Physiotherapy group; n = 15** *Age, mean* ± *SD*: 53.7 ± 13.0 *Stage:* N/A *Sx type, n (%):* BCS + ALDN: 3 (20)/MX + ALDN: 12 (60) *Systemic treatment, n (%):* Ch: 2 (13)/HT: 1 (7)/RT + Ch: 6 (40) Ch + HT: 1 (7)/RT + HT: 1 (7) RT + HT + Ch: 1 (7) **Control group; n = 15** *Age, mean* ± *SD*: 55.4 ± 9.3 *Stage*: N/A *Sx type, n (%):* BCS + ALDN: 4 (27)/MX + ALDN: 11(73) *Systemic treatment, n (%):* RT: 2 (13)/Ch: 2 (13)/HT: 1 (7) RT + Ch: 8 (53)/Ch + HT: 1 (7) RT + HT: 1 (7)	**Physiotherapy group** PT sessions (advice and exercises for arm/shoulder, posture correction, coordination exercises, exercises for muscular strength, improvement of general physical condition, exercises to prevent LE and instruction for ST massage of the scar if required Initiation: 2w following surgery Duration: 3 months Frequency: 1–2/w for the first 3w and then once a fortnight or less + 10 min of home exercises daily **Control group** Leaflet flyer with advice and exercises for the arm/shoulder and had no further contact with the physiotherapist Initiation: 1st w following surgery	Arm/shoulder pain (VAS) Shoulder mobility (digital inclinometer) Disabilities in daily life (DASH) Arm edema (water displacement) Grip strength (hand-held dynamometer) Quality of life (SIP questionnaire short version)
Box [6] (2002) Australia	RCT	N = 65 **Treatment group; n = 33** *Age, mean* ± *SD*: 53.03 ± 9.49 Stage: N/A *Sx type (%):* BCS + ALND: 46.9/MRM: 53.1 **Control group; n = 32** *Age, mean* ± *SD*: 59.00 ± 10.95 Stage: N/A *Sx type (%):* BCS + ALND: 51.5/MRM: 48.5	**Treatment group** *Physiotherapy Management Care Plan (PMCP)* Included a thorough preop assessment and explanation with postop reviews to monitor shoulder ROM, progress exercise program, LE awareness ED and individualized intervention as required **Control group** Exercise instruction booklet	Shoulder ROM (goniometer) Function (12-items functional questionnaire)
Box [7] (2002) Australia	RCT	N = 65 **Treatment group; n = 33** *Age, mean* ± *SD*: 53.03 ± 9.49 *Stage*: N/A *Sx type (%):* BCS + ALND: 46.9/MRM: 53.1 **Control group; n = 32** *Age, mean* ± *SD*: 59.00 ± 10.95 *Stage*: N/A *Sx type* (%): BCS + ALND: 51.5/MRM:48.5	**Treatment group; n = 33** *Physiotherapy Management Care Plan (PMCP)* Included a thorough preop assessment and explanation with postop reviews to monitor shoulder ROM, progress exercise program, LE awareness ED and individualized intervention as required **Control group; n = 32** Exercise instruction booklet	Arm size-*CIRC* Arm VOL (water displacement) Multi-frequency bioimpedance-*MFBIA* (spectroscopy) Incidence of secondary LE (based on preop CIRC, preop VOL and MFBIA ratio)
Cho [8] (2016) South Korea	RCT	N = 48 BC patients with AWS **PTMLD group; n = 24** *Age, mean* ± *SD*: 50.7 ± 9.6 *Stage, n (%):* I: 5 (24)/III: 16 (76) *Sx type, n (%):* MX: 12 (57)/LUMP: 7 (33) Breast recons: 2 (10) *Systemic treatment, n (%):* *Ch:* 9 (43)/*RT:* 21(100)/*HT:* 14 (67) **PT group; n = 24** *Age, mean* ± *SD*: 46.6 ± 6.8 *Stage, n (%):* I: 12 (60)/III: 8 (40) *Sx type, n (%):* MX: 16 (80)/LUMP: 3 (15) Breast recons: 1 (5) *Systemic treatment, n (%):* *Ch:* 11 (55)/*RT:* 19(95)/*HT:* 12 (60)	**PTMLD group** PT program combined with MLD *Supervised PT program* UE strengthening and stretching exercises combined with MT session (ST mobs and stretching, shoulder stretching exercises, shoulder girdle mobs and PROM exercises *Initiation*: At least 4w after BSx *Duration*: 4w *Frequency*: 3 times/w *MLD* Frequency: 5 days/w for 4w MLD sessions duration: 30 min **PT group; n = 24** PT program solely	Arm VOL (CIRC tape measurements) Muscular strength (dynamometer) Active ROM (inclinometer) Pain (NRS-11) Arm disability (DASH) QoL (EORTC QLQ-C30 v3, EORTC QLQ-BR23) Visible cording (Subjective assessment by a rehab doctor)
Cinar [9] (2008) Turkey	RCT	N = 57 **Treatment group; n = 27** *Age, mean* ± *SD*: 51.1 ± 13.0 *Stage, n (%):* N/A *Sx type*: *MRM* *Systemic treatment, n (%):* Ch: 29 (97)/RT: 14 (47) **HB exercise program; n = 30** *Age, mean* ± *SD*: 52.6 ± 12.2 *Stage, n (%):* N/A *Sx type*: *MRM* *Systemic treatment, n(%):* Ch:23(85)/RT:10(4)	**Treatment group** Early shoulder ROM exercises (to be started on the 1st post-op day) and PT program *PT program* Included ROM, stretching and strengthening exercises *Initiation*: Following drains removal *Duration*: 15 supervised sessions and 8w self-A **HB exercise program group** Postoperative exercise forms to perform at home	ROM (Myrin goniometer) Arm VOL (CIRC tape measurements) Function (10-item functional questionnaire)
de Almeida Rizzi [10] (2020) Brazil	RCT	N = 62 **Free ROM group; n = 31** *Age, mean* ± *SD*: 49.90 ± 10.11 *Stage, n (%):*0: 10 (33)/I: 4 (13)/II: 3 (10) IIB: 7 (23)/III: 5 (17)/IIIB: 1(3)/IV: 0 (0) *Sx type, n (%):* Breast sparing Sx: 14 (47) MX: 16 (53)/Breast recons: 30(97) *Type of node dissection, n (%):* SNB: 15 (50)/ALND: 14 (47) *Systemic treatment, n (%):* Neoadj Ch: 13 (43) **Limited ROM group; n = 31** *Age, mean* ± *SD*: 54.46 ± 10.68 *Stage, n (%):* 0: 11 (37)/I: 4 (13)/II: 7 (23) IIB: 5 (17)/III: 2 (7)/IIIB: 0 (0)/IV: 1 (3) *Sx type, n (%):* Breast sparing Sx: 10 (33) MX: 20 (67)/Breast recons: 30(97) *Type of node dissection, n (%):* SNB: 21 (70)/ALND: 7 (23) *Systemic treatment, n(%):*Neoadj Ch:10(33)	**Both groups** Exercise protocol consisting of neck and UL stretching exercises and shoulder ROM exercises *Initiation*: 1st pod *Day1-14:* Exercises 1–6 *From Day15:* Exercises 1–8 **Free ROM group** Were allowed to perform the protocol exercises and ADL in free amplitude **Limited ROM group** Had ROM maintenance limited to 90° until the 30th post-op day, then started free ROM exercises	Dehiscence (Inspection, palpation and tape measure) Seroma (Inspection and palpation, medical record) Infection (Inspection and palpation, medical record) Necrosis (Inspection and medical record) Shoulder ROM (Goniometer) Pain (VAS) Upper limb function (DASH)
De Groef [11] (2017) Belgium	RCT	N = 147 **Intervention group; n = 72** *Age, mean* ± *SD*:53.9 ± 11.5 *Stage, n (%):* 0: 7 (10)/I: 16 (22)/II: 36 (50)/III: 13 (18) IV: 0 (0) *Sx type, n (%):* MX: 46 (64)/BCS: 26 (36) *Systemic treatment, n (%):* RT, IMC and medial supraclavicular:72(100) RT, axilla: 8(11)/Ch: 60(83) Neoadj Ch:29(40) Target therapy: 22(31)/HT: 57(79) **Control group; n = 75** *Age, mean* ± *SD*: 54.7 ± 11.9 *Stage, n (%):*0: 2 (3)/I: 20 (27)/II: 37(48)/III:14(19)/IV:2 (3) *Sx type, n (%):* MX: 50 (67)/BCS: 25 (33) *Systemic treatment, n (%):* RT, IMC and medial supraclavicular:75(100) RT, axilla: 9(12)/Ch:55(73) Neoadj Ch:21(28) Target therapy: 9 (12)/HT: 62 (83)	**Both groups** Individual standard PT program consisting passive mobs, stretching and transverse strain of pectoral muscles, scar tissue massage, exercises schemes, posture and movement control and shoulder AROM *Initiation*: after surgery *Duration:* 4 months *Exercise sessions duration*: 30 min *Frequency*: 2 session/w, reducing to once/w after the first 2 months **Intervention group** Individual standard PT program + MT *Initiation*: 2 months post-surgery *Duration* (MT): 2 months *Freq of MT sessions*: once/w **Control group** Individual standard PT program + placebo t_x_ consisting of static bilateral hand t_x_ at the upper body and arm *Initiation:* 2 months post-surgery *Duration* (placebo): 2 months *Frequency*: once/w *Placebo t*_*x*_* duration*: 30 min	Point prevalence of pain (Yes/No question) Pain intensity (VAS) Pressure hypersensitivity (Digital algometer) Pain quality (McGill Pain Questionnaire) Point prevalence of impaired shoulder function (DASH score of more than 15%) Shoulder function (DASH) Quality of life (SF-36)
De Rezende [12] (2006) Brazil	RCT	N = 60 **Directed exercises group; n = 30** *Age, mean* ± *SD*: 54.00 ± 10.11 *Stage, n (%):* I: 5 (17)/IIA: 4 (13)/IIB: 5 (16) IIIA: 4 (13)/IIIB: 8 (27)/IIIC: 2 (7)/IV: 2(7) *Sx type, n (%):* Halsted RMX: 5(17)/MRM: 16 (53)/QT: 9(30) *Systemic treatment, n (%):* Previous Ch: 8 (27) **Free exercises group; n = 30** *Age, mean* ± *SD*: 55.40 ± 11.24 *Stage, n (%):* I: 6 (20)/IIA: 10 (33)/IIB: 6 (20) IIIA: 3 (10)/IIIB: 3 (10)/IIIC: 0 (0)/IV: 2 (7) *Sx type, n (%):* Halsted RMX: 1(3)/MRM: 21(70)/QT:8 (27) *Systemic treatment, n (%):* Previous Ch: 9 (30)	**Directed exercises group** 19 ROM-exercises program performed in groups of 5 to 20 women and supervised by a team of PT and students *Initiation*: 1st post-op day *Duration*: 3 days/w for 42 days *Exercise sessions duration*: 40 min **Free exercises group** Same ROM exercises program without a previously defined sequence or number of repetitions	Shoulder ROM (Manual goniometer) Lymphatic disturbance (Drainage VOL) Arm CIRC (tape measure)
Devoogdt [13] (2018) Belgium	RCT	N = 160 **Experimental group; n = 79** *Age, mean* ± *SD*: 56 ± 13 *Stage, n (%):* 0: 1 (1)/I: 21 (27)/II: 38 (48)/III: 13 (17) IV: 6 (8) *Sx type, n (%):* MX + ALDN: 52 (66)/BCS + ALDN: 27(34) *Systemic treatment, n (%):* Ch: 50 (63)/Target therapy:14(18)/HT:55(70) **Control group; n = 81** *Age, mean* ± *SD:* 55 ± 11 *Stage, n (%):* 0: 0 (0)/I: 26 (32)/II: 39 (48)/III: 12 (15) IV: 4 (5) *Sx type, n (%):* MX + ALDN: 56 (69)/BCS + ALDN: 25(31) *Systemic treatment, n (%):* Ch: 58 (72)/Target therapy: 7(9)/HT:66(82)	**Both groups** *During hospitalization:* Received information about the prevention of LE + exercise therapy (mobilizing exercises) *After hospitalization:* 30-min individual exercise sessions *Duration*: 6 months *Frequency*: 2 times/w, gradually diminished to 1/2w **Experimental group** Protocol described above + MLD Initiation: one week after removal of axillary drains *Duration of MLD*: 20 weeks Frequency of exercise sessions during this period: one to 3 times/w and then gradually decreased to once/w **Control group** Protocol described above without MLD	Incidence of arm LE (Water displacement, arm CIRC) Point prevalence of arm LE (water displacement, arm CIRC) Point prevalence of subjective arm and trunk LE (Questioned at interview) Arm VOL difference (water displacement) Shoulder ROM-abd, flexion, ext and int rotation (Goniometer, tape measure) HRQoL (SF-36) Problems in functioning (Lymph-ICF)
Fatima [14] (2022) Pakistan	RCT	N = 30 **Treatment group; n = 15** **Control group; n = 15** *Overall mean age (y), mean* ± *SD:* 46 ± 10.75 *Sx type:* All participants were scheduled to undergo unilateral MRM and axillary clearance	**Treatment group** Preoperative EX protocol Initiation: Pre-operative period Freq: 2–3 times/day, 2–5 sessions/w REPS: ROM EX 10–12; stretching EX 8–10 Duration: Preoperative period and was repeated after the 1st and 3rd pod; continued with routine care in the postop period (unclear) **Control group** **Routine care (ROM EX)** Initiation: Pre-operative period Freq: 2–3 times/day REPS: 10–12 Duration: unclear	Pain intensity (NPRS) Shoulder ROM (Goniometer) Ability to perform ADLs (Groningen Activity Restriction Scale-GARS)
Feyzioğlu [15] (2020) Turkey	RCT	N = 40 **Kinect-based rehabilitation group; n = 20** *Age, mean* ± *SD*: 50.84 ± 8.53 Stage, n (%): N/A *Sx type*: Unilateral BSx + ALND *Systemic treatment, n (%):* Ch: 4 (21)/RT: 13 (68)/HT: 2 (11) **Standardized physiotherapy group; n = 20** *Age, mean* ± *SD*: 51.00 ± 7.06 *Stage, n (%):* N/A *Sx type*: Unilateral BSx + ALND *Systemic treatment, n (%):* Ch: 2 (12)/RT: 13 (77)/HT: 2 (12)	**Both groups** Breathing, ROM and pumping exercises, limitations for shoulder ROM amplitudes, weightlifting, jumping and running up to 6w post-op *Initiation*: 1st post-op day *Duration*: 2w **KBR group** Xbox 360 Kinect video game program combined with tissue massage and passive mobs **SPT group** Standard UE PT program including scar tissue massage and mobilizations *Initiation*: 2nd post-op w *Duration:* 2 days/w for 6 w *Program sessions duration:* 45 min	Pain intensity (VAS) Shoulder ROM (Digital goniometer) Shoulder muscle strength (Handheld dynamometer) Handgrip strength (Hydraulic hand dynamometer) Upper extremity function (DASH) Fear of movement (TKS)
Heiman [16] (2021) Sweden	RCT	N = 400 **Intervention group; n = 200** *Age (median; range):* 61 (52–68; 30–84) *Stage, n(%):*I:92(51.4)/II:83(46.4)/III:4(2.2) *Sx type, n(%):* BCS:147(80.3)/MX:36(19.7) *Type of node dissection, n (%):* SNB: 161 (88)/ALND: 11 (6.0) **Control group; n = 200** *Age (median; i.g.r; range):* 63 (54–71; 38–89) *Stage, n (%):* I: 75 (38.7)/II: 108 (55.7)/III: 10 (5.2)/IV: 1 (0.5) *Sx type, n (%):* BCS: 154 (78.2)/MX:43 (21.8) *Type of node dissection, n (%):* SNB: 176 (89.3)/ALND: 9 (4.6)	**Intervention group** Instructions by a PT to add 30 min of aerobic PA daily + 2 follow-up calls Initiation: 1-3w before Sx Duration: up to 4w after discharge from hospital **Control group** Routine care (did not receive any advice regarding PA) **Both groups** Received standardized information from a PT regarding early mobs and shoulder movement before hospital discharge	Physical recovery (self-reported questionnaires, SGPALS) Mental recovery (self-reported questionnaire) Duration of hospital stay Unplanned reoperations and readmissions (retrieved from medical records) Postoperative complications (CCI)
Joo [17] (2021) Korea	RCT	N = 56 **Early shoulder exercise group; n = 28** *Age, mean* ± *SD*: 44.50 ± 6.70 *Sx type, n (%):* MX + Immediate Brecons: 28 (100) *Type of node dissection, n (%):* SNB: 26 (92.86)/ALND: 2 (7.14) **Arm restriction group; n = 28** *Age, mean* ± *SD*: 44.10 ± 8.35 *Sx type, n (%):* MX + Immediate Brecons: 28 (100) *Type of node dissection, n (%):* SNB: 22 (78.57)/ALND: 6 (21.43)	**Early shoulder exercise group** Shoulder ROM exercise routine Initiation: 2nd pod Duration: unclear **Arm restriction group** Any type of arm exercise was restricted until drains removal	Drainage volume Duration of drain placement
Kilbreath [18] (2012) Australia	RCT	N = 160 **Exercise group; n = 81** *Age, mean* ± *SD*: 53.5 ± 12.1 Stage, %: I: 17/II: 44/III: 38 *Sx type, %:* MX + SNB: 48/ALDN: 62 *Systemic treatment, %:* Ch: 68/RT: 79 **Control group; n = 79** *Age, mean* ± *SD*: 51.6 ± 11.0 *Stage, n (%):* I: 19/II: 37/III: 44 *Sx type, %:* MX: 47/ALDN: 58 *Systemic treatment, %:* Ch: 71/RT: 76	**Both groups** Postop care including information outlining arm exercises and prevention of LE *Initiation*: 4-6w post-surgery *Duration*: 8 w **Exercise group:** Resistance training and passive stretching for shoulder muscles + HB program of resistance training and stretching *Initiation*: 4–6 w post-surgery *Duration*: 8w *Frequency*: once/w **Control group** No exercises or advice was provided	Self-reported arm symptoms (EORTC-BR23) Breast symptoms (EORTC-BR23) Shoulder ROM (Digital inclinometer) Upper shoulder muscle strength (hand-held dynamometer) Presence of LE (Bioimpedance spectroscopy)
Kilbreath [19] (2006) Australia	RCT	N = 22 **Exercise group; n = 14** *Age, mean* ± *SD*: 52.7 ± 14.0 *Stage*: N/A *Sx type, n (%):* MX + ALDN: 8 (57)/WLE + ALDN: 6 (43) *Systemic treatment, n (%):* RT: 9(64)/ Ch: 7(50) **Control group; n = 8** *Age, mean* ± *SD*: 51.5 ± 10.2 *Stage*: N/A *Sx type, n (%):* MX + ALDN: 4 (50)/WLE + ALDN: 4 (50) *Systemic treatment, n (%):* RT:7(88)/ Ch:6 (75)	**Exercise group** Usual care + shoulder ROM, strengthening and stretching exercises *Initiation:* 4 to 5w post-surgery *Frequency:* performed daily and supervised once/w by a PT **Control group** Usual care (monitoring by a breast care nurse, may be seen by a PT to review UL exercises and by an OT who discussed prevention of lymphedema) provided at the hospital, were discharged 2 to 7 days post-surgery	Quality of life (EORTC-QLC-C30, EORTC-QLC-BR23) Presence of LE (Arm CIRC measurements) Shoulder ROM (inclinometer) Maximal isometric shoulder strength (dynamometer)
Klein [20] (2021) Israel	RCT	N = 160 **Intervention group; n = 73** *Age, mean* ± *SD*: 53.3 ± 12.7 *Stage, n (%):* IA: 40 (55.6)/IB: 2 (2.8)/IIA: 12(16.7)/IIB:4(5.6)/IIIA:0/IIIC: 1(1.4) *Sx type, n (%): LUMP*: 4 (4.6); *LUMP* + *SNB:* 23 (31.9); *LUMP* + *ALND*: 7 (9.7); *PMMX* + *SNB*: 14 (19.4); *PMMX* + *ALND*: 1 (1.4); *PMMX* + *Brecons*: 23 (31.9) *Systemic treatment, n (%):* Neoadj Ch: 17 (23.6)/Adj Ch: 33 (45.8)/RT: 51 (70.8)/IORT: 8 (11.1) **Control group; n = 87** *Age, mean* ± *SD*: 51.2 ± 13.1 *Stage, n (%):* IA: 34 (40.0)/IB: 5 (5.9)/IIA: 7 (8.2)/IIB:2(2.4)/IIIA: 1(1.2)/IIIC: 0 *Sx type, n (%): LUMP*: 15 (17.6); *LUMP* + *SNB:* 16 (18.8); *LUMP* + *ALND*: 0; *PMMX* + *SNB*: 21 (24.7); *PMMX* + *ALND*: 4 (4.7); *PMMX* + *Brecons*: 29 (34.1) *Systemic treatment, n (%):* Neoadj Ch: 18 (21.1)/Adj Ch: 27 (31.8)/RT: 45 (52.9)/IORT: 4 (4.7)	**Intervention group** PT treatment that included therapeutic, stretching and strengthening exercises + patient education Initiation: 2nd pod Duration: unclear **Control group** Usual nursing care (guidance on postoperative complications and instructions in case of persistence of symptoms more than 3w) *Follow-up:* 1, 3 and 6 m post-op	Pain (NPRS) UL function (QuickDASH) Shoulder ROM (Goniometer application) Presence of LE or AWS (Patient self-reported)
Lauridsen [21] (2005) Denmark	RCT	N = 139 **Group A; n = 72** *Age (age range):* MRM + RT: 49 (40–70)/MRM: 60 (37–74) BCS: 54 (31–79) *Stage*: N/A *Sx type, n (%):* MRM + RT:20(28)/MRM: 21(29)/BCS: 31(43) *Systemic treatment, n (%):* Ch: 26 (36)/RT: 23(32)/HT:25(35) **Group B; n = 67** *Age (age range):* MRM + RT: 51 (29–70)/MRM: 63 (32–77) BCS: 54 (32–69) *Stage*: N/A *Sx type, n (%):* MRM + RT: 23 (34)/MRM: 13(19)/BCS: 31(46) *Systemic treatment, n (%):* *Ch:* 21 (31)/RT: 17(25)/HT: 17(25)	**Group A** Team instructed PT program consisting of relaxation and strengthening exercises, combined to vein pump therapy and stretching of scar tissue *Initiation*: 6th to 8th post-op w *Duration:* 2 days/w for 6w *Exercise sessions duration*: 60 min **Group B** ‘’Standard treatment of the ward’’ and were offered the same PT program after the 26th post-op w	Shoulder function (Constant Shoulder Score) Presence of ‘’strings’’ in the axilla (Physical assessment)
Odynets [22] (2021) Ukraine	RCT	N = 77 **Group A; n = 38** *Age, mean* ± *SD*: 57.10 ± 1.37 *Stage, n (%):* I: 9 (24.0)/II: 29 (76.0) *Sx type, n (%):* Madden MX: 38 (100) **Group B; n = 39** *Age, mean* ± *SD*: 57.40 ± 1.24 *Stage, n (%):* I: 10 (26.0)/II: 29 (74.0) *Sx type, n (%):* Madden MX: 39 (100)	**Group A** Progressive muscular relaxation and visualization exercises + yoga intervention Initiation: 2-3w after surgery Duration: 1 m **Group B** Yoga intervention only Initiation: 2–3 w after surgery Duration: 1 m	Pain experience and intensity (McGill Pain Questionnaire and VAS)
Majed [23] (2020) USA	RCT	N = 69 BC women undergoing MRM **Intervention group; n = 35** *Age group, n (%):* 35–42: 14 (47)/43–48: 10 (33) 49–55: 6 (20) **Control group; n = 34** Age group, n (%): 35–42: 14 (47)/43–48: 10 (33) 49–55: 6 (20)	**Intervention group** *Phase 1 and 2* Measurements: QoL-BC survey and shoulder ROM Intervention: one-to-one ED in addition to routine hospital care. Demonstration of the exercises by the researcher with a return demonstration by the patient was done *Phase 3 (post-surgery)* Deep breathing + shoulder exercises. Shoulder flexion was limited to 90° of assisted AROM until the drains were removed, gradually increased after the 3rd pod **Control group** Routine hospital care that did not include any exercise training or ED. Routine hospital care included explanation by the surgeon on the surgical procedure	Quality of life (Breast Cancer Patient Version (QoL-BC)) Shoulder ROM (Goniometer)
Pace do Amaral [24] (2012) Brazil	RCT	N = 131 **MT + UL exercises group; n = 65** *Age, mean* ± *SD*: 55.0 ± 11.4 *Stage, n (%):* I/II: 46 (72)/III/IV: 18 (28) *Sx type, n (%):* BCS: 15 (23)/RM: 50 (77) *Systemic treatment, n (%):* Ch: 22(88)/RT: 13(52)/HT: 15(60) **UL exercises group; n = 66** *Age, mean* ± *SD:* 56.7 ± 11.7 *Stage, n (%):* I/II: 38 (58)/III/IV: 28 (42) *Sx type, n (%):* BCS: 13 (20)/RM: 53 (80) *Systemic treatment, n (%):* Ch: 27 (90)/RT: 24(80)/HT: 18(60)	**Both groups** Initiated PT on the 1st pod **MT + UL exercises group** UL exercises sessions, followed by an MT protocol consisting of scapular and glenohumeral joint mobs and therapeutic massage *Duration*: 1 month *Frequency*: twice a week *MT sessions duration*: 20 min **UL exercises group** Outpatient physical therapy program combining UL exercises to precautions to prevent LE *Initiation*: 3rd post-op day *Duration*: 1 month *Frequency*: 3 times a week *Exercise sessions duration*: 45 min	Shoulder ROM (goniometer) Upper limb function (Modified-University of California at Los Angeles Shoulder Rating Scale) Postoperative complications (Observations made by the main investigator)
Paskett [25] (2021) USA	RCT	N = 568 **LEAP group; n = 315** *Age, year, median (range):* 58 (27–88) *Grade, n (%):* Low: 65 (22.0)/Intermediate: 138 (46.6)/High: 93 (31.4) *Sx type, n (%):* PMX or LUMP: 199 (64.8); MX: 108 (35.2)/Missing: 5 *Type of node dissection, n (%):* SLND: 158 (50.6)/ALND: 67 (21.5)/SLND + ALND: 87 (27.9) *Systemic treatment, n (%):* Ch: 109 (34.9)/RT: 214 (68.6) **EO group; n = 253** *Age, year, median (range):* 59 (24–83) *Grade, n (%):* Low: 54 (22.5)/Intermediate: 93 (38.8)/High: 93 (38.8) *Sx type, n (%):* PMX or LUMP: 155 (65.1); MX: 83 (34.9)/Missing: 4 *Type of node dissection, n (%):* SLND: 100 (41.3)/ALND: 64 (26.4)/SLND + ALND: 78 (32.2) *Systemic treatment, n (%):* Ch: 95 (39.3)/RT: 166 (68.6)	**LEAP group** LE education and prevention (LE etiology, signs, symptoms, treatments, preventive self-care practices) and exercise program (breathing, stretching, strengthening and ROM EX) Initiation: ≤ 6w after Sx Freq: daily Exercises duration: 15 min Duration: unclear **EO group** LE education and prevention only *Follow-up:* 6, 12 and 18 months after Sx	Presence of LE (arm CIRC) Severity of LE (change in arm CIRC at the site of greatest difference) Shoulder ROM (self-reported) Adherence to study protocol
Petito [26] (2014) Brazil	RCT	N = 77 **Early group; n = 40** *Age, mean* ± *SD*: 55 ± 8 *Sx type, n (%):* MX: 24 (59)/QT: 17 (42) **Late group; n = 40** *Age, mean* ± *SD*: 53 ± 12 *Sx type, n (%):* MX: 21 (57)/QT: 16 (43)	**Exercise program (both groups)** 9 exercises outside hospital with illustrated manual *Duration*: 105 post-operative days *Frequency*: daily at home **Early group** *Initiation*: 1st post op day **Late group** *Initiation*: After drain removal (postoperative day 7–10, mean postoperative day: 9)	Evaluation incision (presence of seroma formation and dehiscence) ROM (goniometer)
Rizzi [27] (2021) Brazil	RCT	N = 60 **Free ROM group; n = 30** *Age (y), mean* ± *SD*: 55.06 ± 10.56 *Stage, n (%):*0:6(20.0)/I:23(76.7)/II: 1 (3.3) *Sx type, n (%):* QT + symmetrization: 26 (86.7); Margin re-excision + symmetrization: 4 (13.3) *Type of node dissection, n (%):* SNB: 23 (76.7)/ALND: 1 (3.3) *Systemic treatment, n (%):* Neoadj Ch: 5 (16.7) **Limited ROM group; n = 30** *Age (y), mean* ± *SD*: 52.53 ± 9.08 *Stage, n (%):*0:3(10.0)/I:23(76.7)/II:4(13.3) *Sx type, n (%):* QT + symmetrization: 29 (96.7); Margin re-excision + symmetrization: 1 (3.3) *Type of node dissection, n (%):* SNB: 2 (66.6)/ALND: 5 (16.7) *Systemic treatment, n (%):* Neoadj Ch: 7 (23.3)	**Both groups** Exercise protocol (UL ROM and cervical muscles stretching EX) Initiation:1st pod Day1-14: Exercises 1–6 From Day15: Exercises 1–8 Duration: unclear **Free ROM group** Were allowed to perform the protocol exercises and ADL in free amplitude **Limited ROM group** Had ROM maintenance limited to 90° until the 30th post-op day, then started free ROM exercises *Follow-up:* PO 60 and 90	Shoulder ROM (Goniometer) Pain intensity (Analog verbal scale) UL function (DASH) Presence of dehiscence, seroma, infection or necrosis (inspection and/or palpation)
Sagen [28] (2009) Norway	RCT	N = 207 **No activity restriction group (NAR); n = 104** *Age, mean* ± *SD*: 54 ± 90.6 *Sx type, n (%):* BSx: 46 (44)/BCS: 57 (55) *Systemic treatment, n (%):* RT, nodes: 47 (45)/RT, breast: 78 (75) Ch: 42 (40)/HT: 48 (46) **Activity restriction group; n = 100** *Age, mean* ± *SD*: 55 ± 90.6 *Sx type, n (%):* BSx: 51 (51)/BCS: 49 (49) *Systemic treatment, n (%):* RT, nodes: 40 (40)/RT, breast: 73 (73) Ch: 38 (38)/HT: 50 (50)	**NAR group** Supervised physical therapy program which emphasized moderate progressive resistance exercise training *Duration*: 6 months *Frequency*: 2–3 times a week *Exercise duration*: 45 min **AR group** Physical therapy program with restricted activities of the OA avoiding heavy (> 3 kg) and strenuous activity *Program*: 6 different passive manual techniques emphasizing flexibility and light massage of the affected shoulder, arm and scar *Duration:* 6 months *Frequency*: 1/week *Program duration*: 45 min	Development of arm LE (VOL diff in mL) Pain and sensation of heaviness (VAS)
Schultz [29] (1997) Sweden	RCT	N = 163 with MRM **Early postoperative shoulder exercise group; n = 89** Age, median (range): 59 (35–83) **Delayed postoperative shoulder exercise group; n = 74** Age, median (range): 62 (41–84)	**Early postoperative shoulder exercise group** Active shoulder exercise (anteflexion, abduction, rotation) *Initiation:* 1st postop day *Frequency:* 3 times/day **Delayed postoperative shoulder exercise group** Active shoulder exercise (anteflexion, abduction, rotation) *Initiation*: 1st postop w *Frequency:* 3 times/day	Shoulder mobility (abduction and anteflexion) Volume of seroma aspirations and number of aspirations
Siedentopf [30] (2013) Germany	RCT	N = 93 **Intervention group; n = 48** *Age, mean* ± *SD*: 55.82 ± 10.72 *Sx type, n (%):* BCS: 29 (62)/RM: 18 (38) SND: 37 (71)/ALND: 15 (29) *Systemic treatment, n (%):* Ch: 17 (53)/RT: 23 (70) **Control group; n = 41** *Age, mean* ± *SD*: 58.41 ± 9.91 *Sx type, n (%):* BCS: 24 (60)/RM: 16 (40) SND: 32 (78)/ALND: 9 (22) *Systemic treatment, n (%):* Ch: 7 (30)/RT: 16 (64)	**Intervention group** Yoga classes *Initiation*: Immediately after Sx *Duration*: 5 w *Frequency*: 2 times/w *Class duration*: 75 min 10 classes over 5 w **Control group** *Yoga classes* *Initiation*: 5 weeks after surgery Duration: 5 w *Frequency:* 2 times/w, 10 classes over 5 w *Class duration*: 75 min Yoga classes: started with lying postures and the gradual mobilization of arms and legs + breathing exercises + dynamic exercises	Quality of life (German version of the European Organization of Research and Treatment of Cancer Quality of Life questionnaire (EORTC QLQ-C30) and its breast-cancer-specific module EORTC QLQ-BR23)
Temur [31] (2019) Turkey	RCT	N = 72 **Intervention group; n = 36** *Age, mean* ± *SD*: 46.7 ± 9.96 *Stage, n (%):* I: 2 (7)/II: 16 (53)/III: 12 (40) *Sx type, n (%):* MRM: 22 (73)/BCS: 8 (27) **Control group; n = 36** *Age, mean* ± *SD*: 45.6 ± 9.03 *Stage, n (%):* I: 2 (7)/II: 16 (52)/III: 13 (12) *Sx type, n (%):* MRM: 17 (55)/BCS: 14 (45)	**Intervention group** Self-management of LE program (SMLP) + exercising program + simple LD *SMLP program:* Training booklet containing information about mechanisms and risk factors of LE and about prevention interventions” *Exercising program:* Hand squeezing exercises, active and passive arm exercises *Frequency:* 3–6 times/day at first and gradually increased to 10 *Exercise sessions duration*: 30–60 min *Duration*: 6 months *Simple lymphatic drainage:* Deep diaphragmatic breathing exercises, neck drainage, axillary drainage and UE drainage *Frequency of breathing exercises*: 3 times a day *Frequency of self-massage*: 2 times a day **Control group** Usual post-op care	Upper extremity function (DASH) Presence of LE-upper extremity CIRC (measuring tape) Quality of life (EORTC QLQ-30 and EORTC QLQ-BR23)
Teodózio [32] (2020) Brazil	RCT	N = 572 **Free ROM group, n = 254** *Age, mean* ± *SD*: 52.54 ± 12.03 *Sx type, n (%):* Segmentectomy: 107 (42) MX: 147 (58) **Restricted ROM group, n = 211** *Age, mean* ± *SD*: 54.53 ± 10.95 *Sx type, n (%):* Segmentectomy: 94 (45) MX: 117 (56)	**Free ROM group** Active UL movements with ROM over 90° (leaflet + home guide) **Restricted ROM group** Active UL movements with ROM restricted to 90° from 1st pod until removal of all surgical stitches (leaflet + home guide) *Initiation*: 1st postop day *Frequency*: 3 times/day (at least once a day)	Presence of seroma Necrosis Dehiscence Hematoma Infection Bruise
Testa [33] (2014) Italy	RCT	N = 70 **Treated group, n = 35** *Age, mean* ± *SD*: 54.3 ± 8.02 *Stage*: N/A *Sx type, n (%):* Maddens’ MRM: 19 (54) Segmental MX + ALDN: 16 (45) *Systemic treatment, n (%):* Ch: 24 (69)/RT: 30 (86) **Control group, n = 35** *Age, mean* ± *SD*: 55.3 ± 8.5 *Stage*: N/A *Sx type, n (%):* Maddens’ MRM: 21 (60) Segmental MX + ALDN: 14 (40) *Systemic treatment, n (%):* Ch: 25 (71)/RT: 27 (77)	**Treated group** Early physical rehabilitation program from latest guidelines for rehabilitation in BC *Initiation*: 2nd postop day *Program duration*: 40 min *Frequency*: 5 times/w during all the duration of axillary drainage Once drainage removed (approximatively postoperative day 7): 20 PT sessions *Frequency*: 5 times/w *Duration*: 60 min/session **Control group:** *N*o early physical rehabilitation program with no instructions of a PT. Rehabilitation program from the old rehabilitation guidelines	Mobility of the glenohumeral joint (goniometer) Grade of pain perceived (VAS) Quality of life (EORTC QLQ30 and QLQ-BR23)
Todd [34] (2008) UK	RCT	N = 116 **Delayed shoulder mobs; n = 58** *Age, mean* ± *SD*: 56.5 ± 12.4 *Stage, n (%):* I: 8 (14)/II: 24 (41)/III: 26 (45) *Sx type, n (%):* WLE: 36 (57)/MX: 24 (43) *Systemic treatment, n (%):* RT: 39 (67)/Ch: 30(52)/HT: 34 (59) **Early full shoulder mobs; n = 58** *Age, mean* ± *SD:* 57 ± 14 *Stage, n (%):* I: 8 (14)/II: 27 (48)/III: 23 (38) *Sx type, n (%):* WLE: 29 (50)/MX: 29 (50) *Systemic treatment, n (%):* RT: 41 (71)/Ch: 26(45)/HT: 41(71)	**Delayed shoulder mobs** Exercise program that limited arm movements < 90° in all planes, followed by a full shoulder ROM program **Early full shoulder mobs** Full shoulder mobilization (i.e., movement > 90°) and shoulder ROM exercises *Initiation:* *Limited ROM program*: 2nd pod *Full ROM program:* 2nd post op w *Exercise sessions duration*: 10 min *Frequency*: 4 times/day until full shoulder ROM was restored and then once/day for the 1st postop year	Incidence of LE-limb VOL difference (Water displacement) Shoulder ROM (Manual goniometer) Grip strength (hand-held dynamometer) Health-related QoL (FACT-B + 4 and SDQ)
Torres [35] (2010) Spain	RCT	N = 120 **Early physiotherapy group; n = 60** *Age, mean* ± *SD*: 52.9 ± 10.7 *Stage*: N/A *Sx type, n (%):* QT: 24 (40)/Modified MX: 23 (38)/LUMP:13 (22) *Systemic treatment, n (%):* RT: 44 (75)/Ch: 50(85)/HT: 39 (66) **ED strategy group; n = 60** Age, mean ± SD: 52.9 ± 12.5 *Stage*: N/A *Sx type, n (%):* QT: 26 (43)/Modified MX: 20 (34)/LUMP:14(23) *Systemic treatment, n (%):* *RT*: 49 (86)/*Ch*: 45(79)/*HT:* 33 (58)	**Early physiotherapy group** MLD + progressive massage of the scar, stretching exercises and progressive active and action assisted shoulder exercises, combined with functional activities and proprioceptive neuromuscular exercises + ED strategy **ED strategy only group** Instruction with printed materials about the lymphatic system, concepts of normal load vs overload, source of 2ndary LE, precipitating factors and 4 preventive interventions *Initiation*: 3 to 5 days after hospital discharge *Duration of both programs*: 3 w *Frequency of both programs*: 3 times/w	Incidence of secondary LE (Arm CIRC)
Wingate [36] (1989) USA	RCT	N = 115 **Treated group, n = 61** *Age, mean*: 56.26 **Control group, n = 54** *Age, mean:* 58.27	**Treated group:** Physical therapy: active hand, wrist, elbow and postural exercises, active and active assisted shoulder exercises, functional activities and PNF After drain removal: HB program with progressive restrictive exercises and PNF *Initiation:* 1st postop day *Duration:* 8 w minimum *Frequency:* 2 session/day Exercise sessions duration: 30 min **Control group** Untreated group with no physical therapy	Psychopathologic self-report inventory (SCL-90-R) Shoulder ROM for flexion and abduction (goniometer) Functional evaluation of the ipsilateral shoulder (Scale of difficulty) Upper extremity CIRC measurement
Zhang [37] (2016) China	RCT	N = 1000 **Physical exercise group; n = 500** *Age group, n (%):* < *50:* 272 (54)/ ≥ *50:* 228 (46) *Stage, n (%):* I/II: 211 (42)/III: 289 (58) *Sx type, n (%):* MRM: 500 (100) **MLD group; n = 500** *Age group, n (%):* < 50: 266 (53)/ ≥ 50: 234 (47) *Stage, n (%):* I/II: 197 (39)/III: 303 (61) *Sx type, n (%):* MRM: 500 (100)	**Physical exercise group** Physical exercise alone *Initiation*: 24 h before surgery with patient ED *Frequency*: pod 1, 2, 3 and day of discharge *Session duration*: 20–30 min *Postop day1-7*: Passive exercises *Frequency*: 3 times/day *Session duration*: 15 min *Postop day7-30*: (after drain removal to sutures removal): Exercises progressed to localized exercises on the affected UL *After removal sutures to 6 months:* Extensive active exercises involving affected shoulder *Frequency*: 3 times/day **MLD group** Physical exercises + Self MLD *Initiation*: after sutures removal *Frequency:* 3 sessions/day *Session duration:* 30 min	Stage of upper limb LE (Observation and tape-measuring) Scar formation (Vancouver Scare Scale) Shoulder function (max. shoulder abduction)
Zhou [38] (2019) China	RCT	N = 92 **Intervention group; n = 46** *Age, mean* ± *SD*: 49.94 ± 8.88 *Stage, n (%):* I: 18 (35)/II: 27 (53)/III: 6 (12) *Sx type, n (%):* MX + SND: 24 (47)/MX + ALND: 15 (29) BCS + SND:10(20)/BCS + ALND:2(4) *Systemic treatment, n (%):* Ch: 41 (80) **Control group; n = 46** *Age, mean* ± *SD*: 49.40 ± 9.88 *Stage, n (%):* I: 14 (28)/II: 29 (57)/III: 8 (16) *Sx type, n (%):* MX + SND: 25 (49)/MX + ALND: 17 (33) BCS + SND: 6(12)/BCS + ALND: 3(6) *Systemic treatment, n (%):* Ch: 43 (84)	**Intervention group** Progressive UL exercises and muscle relaxation training by nurses *Initiation*: before surgery *Duration*: 6 months *Frequency:* 1 session/day at hospital and 1 session/week at home after discharge **Control group** Routine nursing care (surgery district nursing, drainage tube nursing, routine health ED, physical exercises, vital sign monitoring and post-surgery complications)	Quality of function (Constant-Murley Score) HRQOL (FACT-Bv4.0)
Zimmermann [39] (2012) Germany	RCT	N = 67 **MLD group; n = 33** *Age, mean* ± *SD*: 60.3 ± 8.2 Stage, n (%): I: 12 (36)/II: 15 (46)/III: 6 (18) *Sx type, n (%):* BCS: 20 (61)/MRM: 13 (39) SND:14 (42)/ALND: 19 (58) *Systemic treatment, n (%):* Ch: 13 (39)/RT: 22 (67) **Control group; n = 34** *Age, mean* ± *SD*: 58.6 ± 12.2 *Stage, n (%):* I: 11 (32)/II: 16 (47)/III: 7 (21) *Sx type, n (%):* BCS: 20 (59)/MRM: 14 (41) SND: 18 (53)/ALND: 16 (47) *Systemic treatment, n (%):* Ch: 15 (44)/RT: 25 (74)	**Both groups** Exercises of limb and chest physiotherapy *Initiation*: 2nd postop day **MLD group** *Manual lymph drainage* *Initiation:* 14th postop day *Duration*: 6 months *Frequency:* 5 sessions/week **Control group** Applied self-drainage from modification of the method described by Földi and Strönbenreuher	VOL of both arms (water displacement With glass cylinder with water) VOL of LE
de Oliveira [40] (2014) Brazil	Controlled non-randomized clinical trial	N = 96 **Exercise group; n = 48** *Age, mean* ± *SD*: 56.7 ± 15.1 *Stage, n (%):* I: 1 (2)/II: 17 (37)/III/IV: 28 (61) *Sx type, n (%): MRM:* 48 (100) *Systemic treatment, n (%):* *Neoadj Ch:* 22 (48) **MLD group; n = 48** *Age, mean* ± *SD*: 55.6 ± 11.9 *Stage, n (%):* I: 0 (0)/II: 9 (20)/III/IV: 34 (79) *Sx type, n (%):* MRM: 42 (62)/Halsted RM: 1 (2) *Systemic treatment, n (%):* Neoadj Ch: 29 (67)	**Both groups** *ED strategy:* Information leaflets about proper care for the OA and lectures delivered by a multi-D team *Initiation*: 1st post-op day **Exercise group** 19-exercise supervised program including neck and rotator cuff muscles stretching and active assisted and free AROM exercises *Initiation*: 3rd post-op day *Duration:* 2 days/w for 30 days *Exercise sessions duration:* 40 min **MLD group** MLD applied by 3 experienced PT *Initiation*: 3rd post-op day *Duration*: 2 days/w for 30 days *MLD sessions duration*: 40 min	Upper limb CIRC (Measuring tape) Shoulder ROM (Goniometer) Scarring complications (Signs of wound dehiscence, infection, seroma and puncture)
Huo(^41^ (2021) China	Controlled non-randomized clinical trial	N = 93 **Observation group; n = 47** *Age, mean* ± *SD*: 48.5 ± 7.0 *Stage, n (%):* I: 7 (14.9)/II: 22 (46.8)/III: 18 (38.3) *Sx type, n (%):* MRM: 47 (100) **Control group; n = 46** *Age, mean* ± *SD*: 47.8 ± 6.4 *Stage, n (%):* I: 5 (10.9)/II: 27 (58.7)/III: 14 (30.4) *Sx type, n (%):* MRM: 47 (100)	**Observation group** Routine nursing care + personalized rehabilitation EX intervention Initiation: 24 h post-Sx Duration: up to 6 m post-Sx **Control group** Routine nursing care	Immune function (Blood sample) UL edema (arm CIRC) Presence of subcutaneous fluid (Teiler’s approach) Shoulder ROM (Goniometer) UL function (DASH questionnaire, ADL score) QoL (FACT-B)
Na [42] (1999) South Korea	Controlled non-randomized clinical trial	N = 33 **Rehabilitation group; n = 20** *Age, mean* ± *SD*: 43.8 ± 2.1 *Stage*: N/A *Sx type, n (%):* MRM: 15 (75)/Partial MX: 5 (25) **Control group; n = 13** Age, mean ± SD: 46.9 ± 9.8 Stage: N/A *Sx type*, n (%): MRM: 7 (54)/Partial MX: 6 (46)	**Rehabilitation group** Early postmastectomy rehabilitation program *Initiation*: 1st post-op day *Duration*: 4w (40 min of PT and 30 min of exercises) *Frequency*: 4 times/day *1*st* postop day:* Postural exercises, AROM of the shoulder, elbow, wrist, and hands with active use of the involved arm *From the 3*rd* postop day:* Physical modalities for pain relief and therapeutic exercises *After drains removal:* Progressive resistance exercises with an increase in functional activities **Control group** Instructions alone for ROM exercises pertaining to the affected shoulder and postural exercises	Symptoms Checklist (SCL-90-R) Shoulder ROM (Goniometer) Shoulder function (10 items provided by Wingate) Upper limb circumference (Tape measurement)
Oliveira [43] (2018) Brazil	Controlled non-randomized clinical trial	N = 116 **Active exercise group; n = 58** *Age group, n (%):* < *55:* 22 (42)/ ≥ *55:* 31(59) *Stage, n (%):* I: 1 (20)/II: 17 (34)/III/IV: 32 (64) *Sx type, n (%):* MRM Patey: 29 (55)/MRM Madden: 24 (45) RM Halsted: 0 (0) *Systemic treatment, n (%):* Neoadj Ch: 24 (45)/Adj Ch: 8 (36) RT: 16 (73)/HT: 14 (64)/IT: 3(14) **MLD group; n = 58** *Age group, n (%):* < *55:* 24 (45)/ ≥ *55:* 29 (55) *Stage, n (%):* I: 0 (0)/II: 9 (18)/III/IV: 43 (82) *Sx type, n (%):* MRM Patey: 19 (36)/MRM Madden: 33 (62) RM Halsted: 1 (2) *Systemic treatment, n (%):* Neoadj Ch: 36 (68)/Adj Ch: 18 (62) RT: 26 (87)/HT:18 (60)/IT: 5 (17)	**Both groups** Educational strategy: Information leaflets about proper care for the OA and daily active exercises to do at home) + lectures delivered by the multidisciplinary team during the first month after surgery *Initiation:* 1st postop day **Active exercise group** *Initiation*: 48 h after surgery *Duration:* 30 days *Frequency:* 40 min group session, 2/w **MLD group** Manual lymphatic drainage *Initiation*: 48 h after surgery *Duration*: 30 days *Frequency:* 40 min individual session, 2/w	Velocity visualization of axillary lymph nodes and degree uptake in axillary lymph nodes (Lymphoscintigraphy) ROM Upper limb CIRC
Tirolli Rett [44] (2022) Brazil	Controlled non randomized clinical trial	N = 65 *Age, mean* ± *SD*: 50.61 ± 11.14 *Sx type, n (%):* MRM: 40 (81.6); QT: 9 (18.4) *Systemic treatment, n (%):* Ch: 29 (59.1)/RT: 23 (46.9)	**PT protocol** Initiation: Between 4-8w after Sx Freq: 3 times/w Sets/Reps: 3/8–12 Consultation duration: 60 min Duration: 20 sessions, 7w	Shoulder ROM (Goniometer) Pain intensity and experience (VAS and McGill Pain Questionnaire)
Kim [45] (2019) South Korea	Retrospective case–control study	N = 115 **Early rehabilitation group; n = 49** *Age (age range):* 43 (34–61) *Stage*: N/A *Sx type*: Skin-sparing total MX and immediate Brecons with tissue expander *Type of node dissection, n (%):* SNB: 41 (84)/ALND: 8 (16) *Systemic treatment, n (%):*Neoadj Ch: 3 (6) **Conventional protocol; n = 66** *Age (age range):* 42 (24–61) *Stage*: N/A *Sx type*: Skin-sparing total MX and immediate Brecons with tissue expander *Type of node dissection, n (%):* SNB: 46 (70)/ALND: 20 (30) *Systemic treatment, n (%):*Neoadj Ch:7 (11)	**Both groups** Self-exercise ED *Initiation*: 1st post-op w **Early rehabilitation group** Short term immobilization period (2w) followed by a self-exercise program including progressive shoulder stretch exercises and strengthening exercises *Initiation*: 3rd post-op w *Frequency*: 4 times a day/7 days per w **Conventional protocol** Were asked to immobilize the OA for more than 4w and engaged themselves in the same self-exercise program after the immobilization period *Initiation:* From the 5th post-op w *Frequency*: 4 times a day/7 days per w	Shoulder ROM (goniometer) Pain (NRS-11) QoL (SF-36) Upper limb function (DASH) Postoperative complications (Plastic surgeon assessment)
Lu [46] (2015) Taiwan	Retrospective cohort study	N = 1217 **Group A; n = 415** *Age, mean* ± *SD*: 51.79 ± 11.97 *Stage, n (%):*0–2: 326 (79)/3: 89 (21) *Sx type, n (%):* BCS: 123 (30)/Simple MX: 25 (6) MRM: 267 (64) *Systemic treatment, n (%):* RT: 182 (44)/Ch: 342 (82) **Group B; n = 672** *Age, mean* ± *SD*: 52.67 ± 11.01 *Stage, n (%):*0–2: 503 (75)/3: 169 (25) *Sx type, n (%):* BCS: 152(23)/ Simple MX:11(2)/MRM: 509(76) *Systemic treatment, n (%):* RT: 297 (44)/Ch: 549 (82) **Group C; n = 130** *Age, mean* ± *SD*: 51.88 ± 10.08 *Stage, n (%):* 0–2: 92 (71)/3: 38 (29) *Sx type, n (%):* BCS: 303(25) Simple MX: 41(3)/MRM: 873(72) *Systemic treatment, n (%):* RT: 66 (51)/Ch: 111 (85)	**Group A** No ED or PT provided **Group B** ED only which provided information on the lymphatic system, the symptoms and signs of LE, suggestions for preventing LE **Group C** ED + PT sessions which included the following treatments: breathing exercise, postsurgical positioning, massaging of scar tissue, mobs of the shoulders and UE exercises, passive and active stretching of the major and minor pectoral muscles *Initiation*: 1st postop w in the hospital and was continued at outpatient clinics post discharge *Frequency*: 2 times/w PT sessions duration: 30 min	Occurrence of LE (Limb-to-limb CIRC difference) LE severity (Criteria defined by the International Society of Lymphology)
Manfuku [47] (2021) Japan	Retrospective case–control study	N = 153 **BME + PT group; n = 78** *Age, mean* ± *SD*: 54.2 ± 9.8 *Stage, n (%):* 0-I: 28 (48.3)/II-III: 30 (51.7) *Sx type, n (%)* MX: 28(48.3); BCS:30(51.7) *Type of node dissection, n (%):* SNB: 39 (67.3)/ALND: 19 (32.8) *Systemic treatment, n (%):* Ch: 23 (39.7)/RT: 37 (63.8)/HT: 42 (72.4) **PNE + PT group; n = 75** *Age, mean* ± *SD*: 52.3 ± 11.3 < *Stage, n (%):* 0-I: 35 (58.3)/II-III: 25 (41.7) *Sx type, n (%):*MX: 37(61.7); BCS:23(38.3) *Type of node dissection, n (%):* SNB: 42 (70.0)/ALND: 18 (30.0) *Systemic treatment, n (%):* Ch: 17 (28.8)/RT: 32 (53.3)/HT: 43 (71.7)	**BME + PT group** PT program that comprised shoulder joint EX and mobs + educational sessions on breast anatomy and surgical procedures Initiation: 1w before Sx Duration: 3 m **PNE + PT group** PT program + educational sessions on pain mechanisms (purpose was to change the patient’s knowledge of their pain states) Initiation: 1w before Sx Duration: 3 m *Follow-up*: 1 year after Sx	Pain intensity and pain interference (BPI) Shoulder ROM (Goniometer) Handgrip strength (Dynamometer) CS-related symptoms (CSI) Pain-related catastrophizing (PCS) Presence of LE (arm CIRC)
Morimoto [48] (2003) Japan	Prospective observational study	N = 72 BC women stage I or II **PCM group; n = 33** *Age, mean* ± *SD*: 50.0 ± 11.0 *Stage*: N/A *Sx type*: PCM **BCS group; n = 38** *Age, mean* ± *SD*: 50.8 ± 8.8 *Stage*: N/A *Sx type*: BCS	**Both groups** Initiation: postoperative day 1 Duration: After hospital discharge, was entrusted to the patient’s own initiative *Postoperative day 1:* Prevention of development of rigidity of shoulder joint on the OA: Lateral and forward arm raising on the affected side in the dorsal sitting positions *Postoperative day 2:* Training for force releasing through exercise of shoulder joint *Postoperative day 3:* Exercise to approximate preoperative life *Postoperative day 4:* Exercise to reduce functional differences between the normal and affected sides	Shoulder joint ROM (goniometer) Grip strength Pain after surgery Movement associated chest pain Operative wound pain ADL (Ability to sleep on the affected side, ability to tie an apron, ability to air the futon in the sun)
Paolucci [49] (2021) Italy	Prospective cohort study	N = 38 *Age, mean* ± *SD*: 57.40 ± 1.24 *Sx type, n (%):* Total MX + breast prostheses or tissue expanders: 38 (100)	**Rehabilitative treatment group** Relaxation and breathing exercises, stretching, GH joint ROM EX, cervical pumping, isometric strengthening EX Initiation: unclear Freq: 2 times/w Rehabilitation sessions duration: 1 h Duration: 5w + 2 m at home *Follow-up*: 1 year	Pain intensity (VAS) QoL (EORTCQLQ-C30) Personality Traits (MMPI-2)
Scaffidi [50] (2012) Italy	Prospective observational study	N = 83 **Group A; n = 25** *Age, mean* ± *SD*: 49.6 ± 8.8 *Sx type, n:* *LUMP:* 10 with 7 SND and 3 ALND *RM:* 15 with 2 SND and 13 ALND **Group B; n = 58** *Age, mean* ± *SD*: 52.1 ± 11.9 *Sx type, n:* *LUMP:* 35 with 26 SND and 9 ALND *RM:* 23 with 6 SND and 17 ALND	**Group A** Preoperative information orally + home rehabilitation program **Group B** Preoperative information orally + information materials + PT treatment at hospital + home rehabilitation program PT at hospital: 1 per day, 30–40 min Home rehab program: 3 times/day	Shoulder arm mobility (goniometer) Upper limb function (Constant and Murley Score) Presence of LE (Universal level meter)
Springer [51] (2010) USA	Prospective observational study	N = 94 Age, mean ± SD: 53.39 ± 11.80 *Stage, n (%):* 0: 11 (12)/I: 40 (43)/II: 30 (32)/III: 13 (14) *Sx type, n (%):* BCT: 41(44)/MRM: 50(53)/Simple MX: 3(3) Lymph nodes dissection, n (%): None: 8 (9)/SND: 20 (21)/ALND: 66 (70) *Systemic treatment, n (%):* Ch: 57 (61)/RT: 64 (66)/HT: 67 (7)	**Upper Limb ROM program** Flexion, abduction, internal and external rotation Pre-operative examination: subjects were instructed in a post-operative UL ROM exercise program, and were educated regarding UL LE precautions and physical exercise initiation and progression *Initiation*: post-surgery Reviewed at 1 month	Pain (NRS) Bilateral shoulder ROM (Goniometer) Bilateral shoulder strength (Break testing of upper limbs) Volume and girth measurements for both upper limbs in standard position (Optoelectronic volumeter, Perometer®) Upper limb function and disability (Upper Limb Disability Questionnaire)
Hsieh [52] (2008) USA	Pretest and post-test quasi-experimental study	N = 96 Women referred by local oncologists for rehabilitative exercises *Sx type:* N/A *Stage*: N/A **Surgery alone; n = 22** *Age, mean* ± *SD*: 55.6 ± 11.3 **Surgery and Ch; n = 30** *Age, mean* ± *SD*: 55.6 ± 11.0 **Surgery and RT; n = 17** *Age, mean* ± *SD*: 57.2 ± 9.4 **Surgery, Ch and RT; n = 27** *Age, mean* ± *SD*: 63.1 ± 9.8	**All groups** Individualized exercise intervention based on the results of the medical and cancer history, physical examination, and the initial physiologic and psychological assessments *Initiation*: immediately following treatment for BC *Exercise sessions duration* 10-min warm-up, 40-min of aerobic exercises, resistance training and stretching and concluded with a 10-min cooldown *Intensity*: 40–65% of HR reserve (based on the treadmill assessment results)	Cardiovascular endurance (Bruce Treadmill Protocol; HR, BP, predicted VO_2max_, time on treadmill and oxygen saturation) Pulmonary function-FVC, FEV_1_ (Flowmate™ spirometer) Cancer-related fatigue (Piper Fatigue Scale)
Petito [53] (2012) Brazil	Quasi-experimental, before and after study	N = 64 **Mastectomy group; n = 43** *Age, mean* ± *SD*: 52.2 ± 9.6 *Sx type, n (%):* MRM: 37(86)/ Simple MX:4 (9)/RMX:2 (5) **QT group; n = 21** *Age, mean* ± *SD*: 63.4 ± 9.0	**Exercise program** *Initiation*: 1st post-op day *Duration*: 105 post-op days *Frequency*: daily *Phase 1 (until drain removal):* Two stretches for the cervical region, two exercises for movement of the scapular girdle, one for shoulder flexion and one for extension beyond the midline *Phase 2 (after drain removal)* Three additional exercises: one exercise for flexion and two for abduction of the shoulder	Shoulder ROM: flexion, extension, abduction (goniometer)
Singh [54] (2013) Canada	Quasi-experimental pretest post-test study	N = 73 **Experimental group; n = 42** *Age, mean* ± *SD*: 55.1 ± 14.8 *Stage, n (%):*0 or I: 2 (5)/II: 14 (34) III: 19 (46)/N/A: 6 (15) *Sx type, n (%):* MRM: 22 (54)/Simple MX: 7 (17) BCS: 12 (29)/B recons: 22 (54) *Systemic treatment, n (%):* RT: 22 (54)/Ch: 16 (39) **Comparison group; n = 31** *Age, mean* ± *SD:* 62.8 ± 14.1 *Stage, n (%):* 0 or I: 2 (7)/II: 10 (32) III: 13 (42)/N/A: 6 (19) *Sx type, n (%):* MRM: 7 (23)/Simple MX: 9 (29) BCS: 15 (48)/Brecons: 3 (10) *Systemic treatment, n (%):* RT: 14 (45)/Ch: 16 (32)	**Experimental group** Standardized preoperative ED + PT treatment if needed focusing on teaching self-management strategies, scar tissue massage and AROM and assisted shoulder exercises *Standardized preoperative ED program:* General postop mobility exercises AROM exercises ED on LE Scar management **Comparison group** Standardized preoperative ED alone	Arm mobility-Shoulder ROM (goniometer) Presence of LE (Arm CIRC, tape measure) UE strength (Manual muscle testing) UE function (DASH) Quality of life (FACT-B + 4) Postoperative pain (VAS)
Rekha [55] (2020) India	Quasi-experimental study	N = 20 *Age range*: 40–60 *Sx type*: Unilateral BSx (MX or BCS) within a month	**Group A; n = 10** Swiss ball exercises + diaphragmatic breathing exercises (10 repetitions) *Duration:* 4w; 5 days/w **Group B; n = 10**: Stretching exercises + diaphragmatic breathing exercises (10 repetitions) *Duration:* 4 w; 5 days/w	Chest expansion (inch tape) FEV_1_ (computerized spirometer) Shoulder ROM (goniometer)
Kilgour [56] (2008) Canada	Pilot study	N = 40 **Home-based exercise (HBE) group; n = 20** *Age, mean* ± *SD*: 50.6 ± 9.3 *Stage, n (%):* N/A *Sx type*: MRM + ALDN **Usual care (UC) group; n = 20** *Age, mean* ± *SD*: 49.1 ± 5.7 *Stage, n (%):* N/A *Sx type*: MRM + ALDN	**HBE group** HB exercise video program that incorporated the exercises and guidelines described in a brochure from CCS *Initiation*: 3rd postop day *Phase 1 (Day3-9):* Self-adm shoulder ROM and flexibility exercises *Frequency*: 3 set/day *Sets duration*: 5–7 min *Phase 2 (Day 10–14):* Same exercises as Phase 1 *Frequency*: 2 sets/day *Sets duration*: 10–15 min **UC group** Received information on diet and skin scare and a 9-page brochure containing stretching and ROM shoulder exercises printed by the CCS, without further instructions	Shoulder ROM (goniometer) Shoulder strength (Manual muscle testing techniques) Grip strength (Hand-grip dynamometer) Forearm CIRC (Tape measurement) Frequency of medication intake, VOL of fluid from the axillary drains and self-perceived pain level (CR-10 Pain Scale) and exertion (Borg Scale)
Baima [57] (2017) USA	Feasibility study	N = 60 *Age, mean, stage and systemic treatment*: N/A *Sx type*: MX or lumpectomy **Group 1-in person teaching; n = 36** **Group 2-video-only teaching; n = 24**	**Both groups** Prehabilitation exercise program and postsurgery shoulder ROM exercises restrictions > 90° until drains were removed *Initiation* 1-4w prior to surgery *Frequency*: once daily, suspended postsurgery **Group 1-in person teaching** Physical demonstration and instructions of supervised shoulder ROM exercises **Group 2-video-only teaching** Instruction’s sheet of shoulder ROM exercises and optional exercises video without additional supervision	Pain (NRS-11) Shoulder abduction ROM (Goniometer) Postoperative seroma formation

ADL: Activities of daily living; Adj Ch: Adjuvant chemotherapy; ALND: Axillary lymph node dissection; AROM: Active range of motion; AWS: Axillary web syndrome; BC: Breast cancer; BCS: Breast conserving surgery; BP: Blood pressure; BSx: Breast surgery; B recons: Breast reconstructive surgery; CCS: Canadian Cancer Society; CIRC: Circumference; Ch: Chemotherapy; CG: Control group; DASH: Disabilities of the Arm, Shoulder and Hand; DXA: Dual-energy X-ray absorptiometry; ED: Education; EORTC QLQ: European Organization for Research and Treatment of Cancer quality of life questionnaire; FACT-B: Functional Assessment of Cancer Therapy-Breast; FEV_1_: Forced expiratory volume in one second; FVC: Forced vital capacity; HB: Home-based; HR: Heart rate; HRQOL: Health-related quality of life; HT: Hormonotherapy; IG: Intervention group; IORT: Intraoperative radiotherapy; IVLD: Interlimb volume difference; KBR: Kinect based rehabilitation; LE: lymphedema; LUMP: Lumpectomy; MFBIA: Multi-frequency bioimpedance; min: minutes; MLD: Manual lymphatic drainage; Mobs: mobilizations; MRM: Modified radical mastectomy; MT: Manual therapy; MX: Mastectomy; N/A: Not available; Neoadj: Neoadjuvant; NRS: Numeric Rating Scale; OA: Operated arm; OT: Occupational therapist; PCM: Pectoral muscle-conserving mastectomy; PROM: Passive range of motion; PT: Physical therapy(ist); QT: Quadrantectomy; RCT: Randomized controlled trial; RM: Repetition maximum; RMX: Radical mastectomy; ROM: Range of motion; RPE: Rated Perceived Exertion; RT: Radiotherapy; SD: Standard deviation; SDQ: Shoulder Disability Questionnaire; SIP: Sickness Impact Profile; SNB: Sentinel lymph node biopsy; SND: Sentinel lymph node dissection; ST: Soft tissue; Sx: Surgery; TKS: Tampa Kinesiophobia Scale; UE: Upper extremity; UL: Upper limb; VAS: Visual Analog Scale; VOL: Volume; w: week; WLE: Wide local excision; 6MWT: 6-Minute Walk Test

### Participants

The studies’ sample size varied from 22 to 1217 participants, with participants’ mean age ranging from 44.1 to 63.4 years old. Most studies (34 out of 57) included women diagnosed with different breast cancer stages (i.e., 0-III), who underwent breast surgery combined with either an axillary staging procedure or ALND, and systemic treatments (i.e., chemotherapy, hormonotherapy, or radiotherapy). In 80.7% of the studies (46 out of 57), the study groups included patients who underwent BCS and those who underwent a total mastectomy. Therefore, no conclusions could be drawn as to whether the type of surgery might have an impact on clinical outcomes and on breast cancer patients' motivation to engage in and complete a rehabilitation intervention.

### Conservative rehabilitation interventions

Four main modalities were identified amongst rehabilitation programs, which were consistent with exercises, patient education, MLD, and manual therapy. Exercises were part of every rehabilitation program, with 43.9% (25 out of 57) of these interventions being unimodal. Multimodal interventions were characterized by 2 to 4 modalities, the most common combinations being: (1) exercise and patient education (40.6%); (2) exercise and manual therapy (15.6%); (3) exercise, patient education, and manual therapy (15.6%); and (4) exercise, patient education, and MLD (12.5%). Nearly half of rehabilitation interventions (47.4%) were delivered using a mixed approach, initially performed under nursing staff or physical therapists' supervision and, in most instances, transitioned to a home-based intervention upon hospital discharge. Home-based interventions (15.8%) all consisted of exercises, which were either performed alone [38, 43, 68, 72], combined with patient education [36, 56, 64, 74] or with manual therapy [63]. Six studies reported implementing group interventions consisting solely of supervised exercise programs [30, 41, 49, 55, 60] or exercise combined with manual therapy [37].

**Fig. 2:**
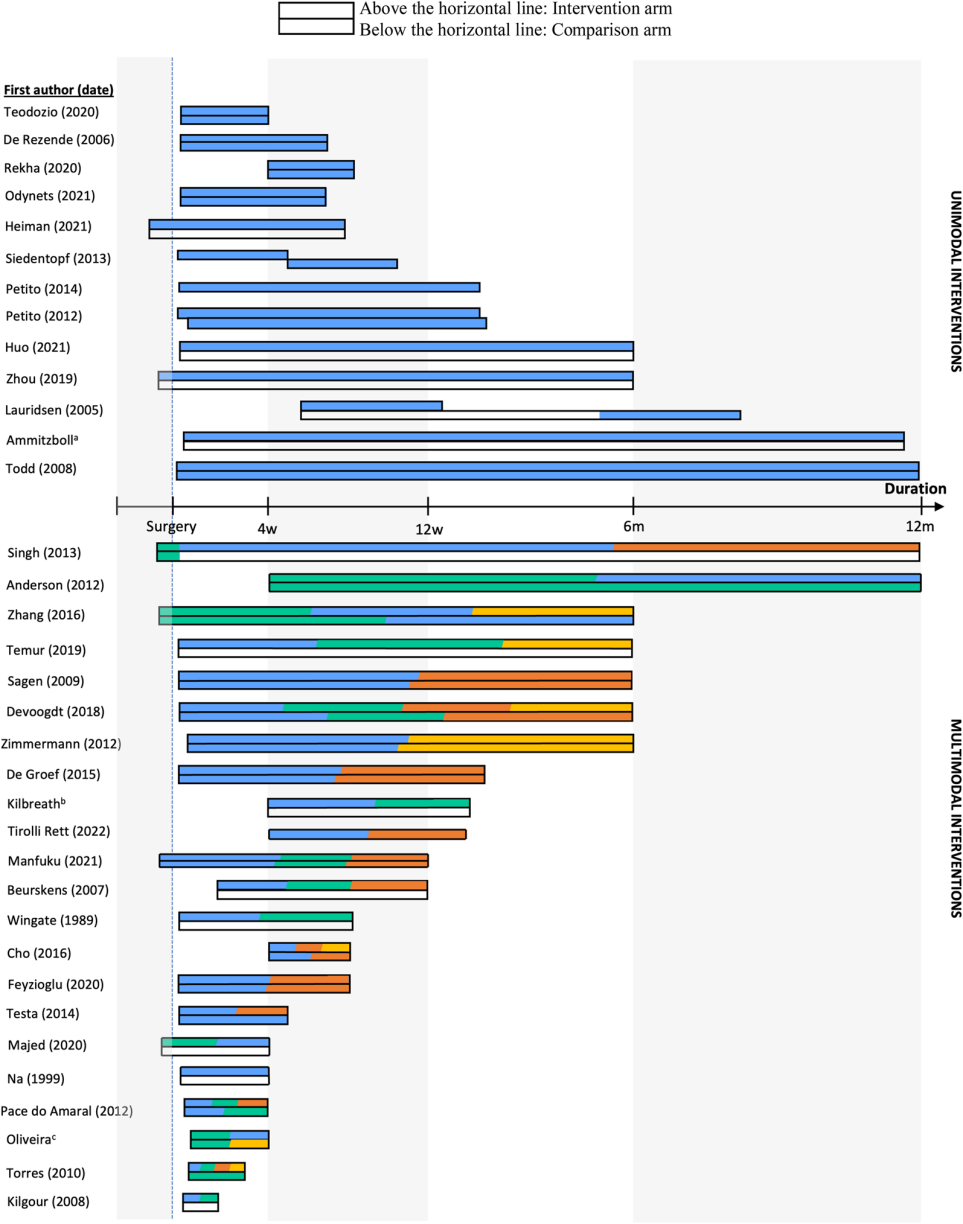
Initiation, duration and modalities characterizing rehabilitation interventions. ^a^Ammitzboll, Kristina Kjaer et al., 2019 & Ammitzboll, Johanssen et al., 2019; ^b^Kilbreath et al. [33, 34] ^c^Oliveira et al. [58, 60]  Blue: Exercises; Orange: Manual therapy; Yellow: Lymphatic drainage; Green: Education; White: Usual care

Figure 2 illustrates the identified rehabilitation interventions’ timing, duration, and modalities. This graphical representation was constructed only for studies that clearly defined all three components. Looking at these studies (35 out of 57), we noted that 74.3% of interventions were initiated a few days to 4 weeks following surgery and went on for 2 to 24 weeks, while 3 interventions [21, 67, 73] lasted up to 12 months.

#### Preoperative rehabilitation interventions

Out of all included studies, 10 interventions [36, 48, 49, 51, 52, 61, 64, 69, 73, 75] were initiated before surgery, including eight that were pursued from 4 weeks to 12 months post-surgery. Only patient education and active shoulder ROM or aerobic exercises were implemented in the preoperative period. Six studies used these modalities as stand-alone, while 4 studies [48, 61, 64, 69, 73] combined them postoperatively with manual therapy or MLD. Educational strategies primarily focused on sharing information about postoperative complications, activity restrictions, prevention of lymphedema, infections or injuries, and explaining the upcoming surgical procedure.

#### Exercises

**Fig. 3:**
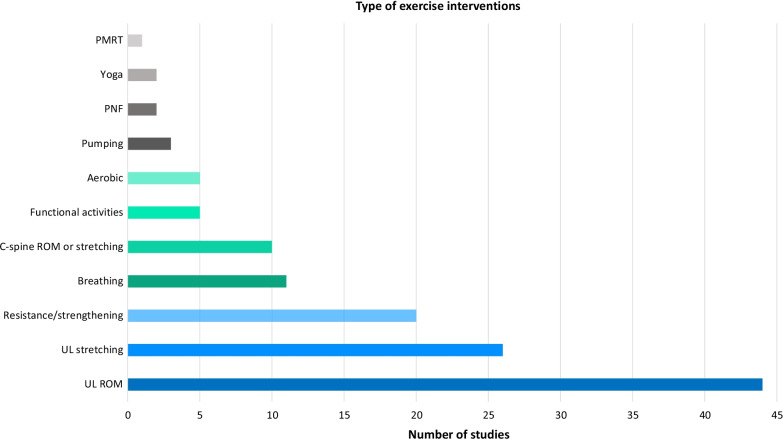
Types of exercise used in rehabilitation interventions. PMRT: Progressive muscle relaxation training; PNF: Proprioceptive neuromuscular facilitation; C-spine: Cervical spine; ROM: Range of motion; UL: Upper limb

Types of exercises included in the rehabilitation programs are detailed in Fig. 3. Eleven types of exercises were identified, the most frequently reported being: (1) upper limb ROM exercises (77.2%); (2) stretching of shoulder muscles (45.6%); and (3) upper limb strengthening exercises (35.1%). Although a small proportion of studies (21.1%) suggested a single type of exercise, most built programs including 2–5 different types. Exercises targeting upper limb tissues and function were predominant. Fewer studies adopted a more global approach, providing aerobic exercises [21, 23, 52, 66, 71] or yoga [41, 55], as well as strengthening or stretching of the lower extremity [19–21] or neck muscles [28, 44, 57, 58, 60–63, 66, 72].

#### Patient education

Educational strategies were included in 42.1% (24 out of 57) of rehabilitation interventions identified. Prevention and lymphedema awareness, skincare routine, risks of postoperative complications, and physical activity or nutrition counseling were the cornerstones of these strategies. Nine studies also reported prescribing shoulder ROM limitations and activity restrictions (e.g., avoiding lifting, carrying heavier items, running, jumping, or other strenuous activities) up to 6 weeks following surgery [22, 32, 39, 51, 57] or upon surgical drains removal [36, 53, 54, 75].

#### MLD

Eight studies [26, 31, 42, 46, 48, 50, 58, 60] included MLD within their rehabilitation programs. Gentle pressure and circular massage were generally applied along the course of superficial lymph nodes lining the axillary region, the lateral aspect of the shoulder, the base of the neck, the chest region, and the affected and non-affected arm and hand. MLD was performed either by trained physical therapists or self-administered following supervised sessions. While most studies reported initiating this modality a few days following surgery without further indications, two studies [31, 48] described waiting for suture and surgical drain removal before proceeding.

#### Manual therapy

Thirteen studies incorporated manual therapy into their rehabilitation programs. This modality was always paired with exercises and, in some cases, complemented with MLD [26, 31, 46]^.^ Passive scapular and shoulder joint mobilizations, scar tissue massage and passive shoulder muscle stretching performed by trained physical therapists [23, 26, 31, 32, 37, 46, 63, 69, 73] mainly characterized manual therapy. Two studies also included passive mobilizations of the elbow, wrist, and hand on the affected side [31, 44].

### Reporting of interventions

Details of interventions’ components were extracted using the TIDieR checklist and guide [76] and are provided in Additional file 2: Table S2. Almost all studies (55 out of 57) reported more than 50% of TIDieR checklist items. Only 3 studies reported modifications to their protocol, and 17 out of 57 provided details regarding intervention adherence. Although 87.72% of studies described the intervention schedule, 15 studies did not specify the duration of interventions.

### Clinical outcome measures

**Fig. 4:**
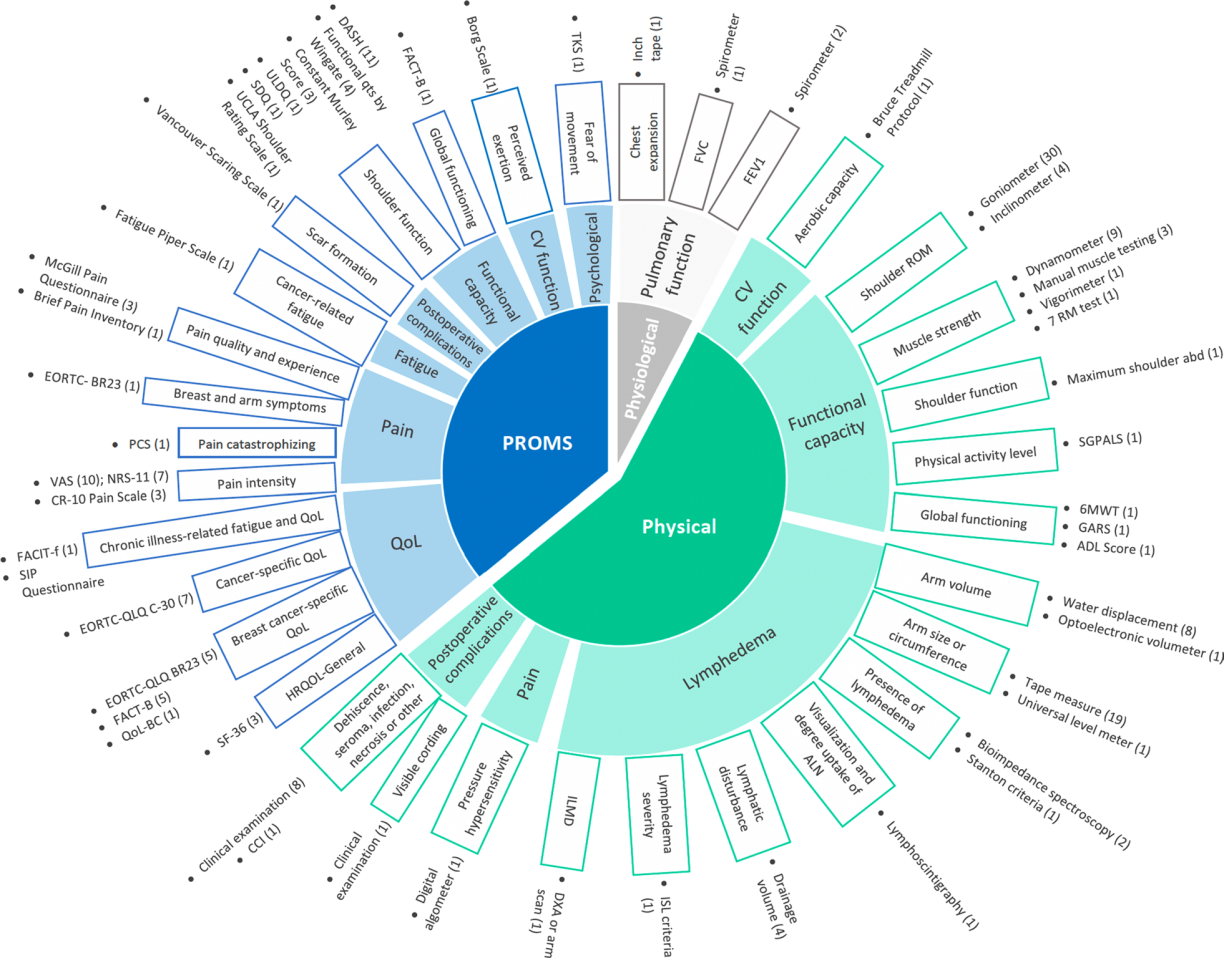
Outcome measures. Abd: Abduction; ADL: Activities of daily living; ALN: Axillary lymph nodes; BC: Breast Cancer; CCI: Comprehensive complication index; CR-10: Borg’s Category Scale for Ratings of Perceived Pain; CV: Cardiovascular; DASH: Disabilities of the Arm, Shoulder and Hand; DXA: Dual-energy X-ray absorptiometry; EORTC QLC: European Organization for Research and Treatment of Cancer quality of life questionnaire; FACT-B: Functional Assessment of Cancer Therapy—Breast; FACIT-f: Functional Assessment of Chronic Illness Therapy – Fatigue; FVC: Forced vital capacity; FEV1: Forced expiratory volume in one second; GARS: Groningen Activity Restriction Scale; HRQOL: Health-related quality of life ILMD: Interlimb mass difference; ISL: International Society of Lymphology; NRS: Numeric Rating Scale; PCS: Pain Catastrophizing Scale; PROMS: Patient-reported outcome measures; QoL: Quality of life; RM: Repetition Maximum; ROM: Range of motion; SDQ: Shoulder Disability Questionnaire; SF-36: 36-Item Short Form Health Survey; SGPALS: Saltin-Grimpy Physical Activity Level Scale; SIP: Sickness Impact Profile; TKS: Tampa Kinesiophobia Scale; UCLA: University of California at Los Angeles; ULDQ: Upper Limb Disability Questionnaire; VAS: Visual analogue scale; 6MWT: 6-Minute Walking Test

Three categories of outcome measures were used to report the effects of rehabilitation interventions on breast cancer patients undergoing mastectomy, including objective measures of physiological and physical function and patient self-reported outcome measures (PROMS). Figure 4 illustrates the outcomes investigated in each category and the measurement tools used for each. Thirty-three unique outcome measures (i.e., 15 physical, 15 PROMS, and 3 physiological) were used across studies, using 54 different measurement tools. Each study used a range of 1 to 7 outcomes, and most studies (37 out of 57) included outcomes from at least 2 of the 3 categories, all of which but one combined PROMS with objective measures of physical function. The most reported outcomes of physical function were shoulder ROM, muscle strength, and signs of lymphedema, measured by the goniometer, the dynamometer and arm circumference or volume, respectively. Quality of life (QoL), shoulder function, and pain were the PROMS most often reported. The European Organization for Research and Treatment of Cancer questionnaire (EORTC QLC C-30/BR23), the Disability of the Arm, Shoulder and Hand questionnaire, and the Visual Analogue Scale were the most frequently used outcome measures for these three domains. Three studies also investigated objective measures of physiological function, such as chest expansion [70], the forced expiratory volume in one second (FEV1) [70, 71], and the forced vital capacity (FVC) [71].

### Patients’ experience

#### Study participation

Twenty-one of the 57 selected studies reported the number of patients who chose not to engage in rehabilitation interventions. Refusal rates ranged between 2 and 75% (MED = 9.0; IQR = 30), with 5 studies reporting rates higher than 40%. The main reasons cited for refusal were disclosed in only 6 studies. They involved transportation issues [21, 29, 35], a preference for another intervention [23, 31, 37] or requesting their own therapist [29, 31], lack of interest [29, 31] and a desire to minimize hospital appointments in favor of getting back to work, and to a normal lifestyle [45].

#### Compliance with the study protocol

Adherence to rehabilitation interventions was measured in 19.3% of studies (11 out of 57) and deemed reasonable in each case (see Additional file 2: Table S2 for details). Coordinating therapy sessions with oncologist appointments [21, 39, 45], follow-up calls and positive reinforcement by physical therapists [24, 25, 42, 74], individualization of interventions based on the patient's needs [71], support from spouses or family members [74] and obtaining positive effects from the intervention [55] were identified as factors promoting adherence. Dropout rates were reported in 31 of the 57 included studies and were highly heterogeneous, ranging from 1 to 58% (MED = 10.0; IQR = 12.8). Main reasons stated for not completing the study were undergoing another breast surgery [19, 20, 22, 28, 38, 39, 42, 43, 72, 75], death [19, 20, 22, 24, 31, 35, 37–39, 45, 56, 60, 63], cancer recurrence or other medical conditions [19, 20, 22, 24, 31, 32, 35], having to deal with systemic treatment-related AEs [31, 32, 39, 41, 42], moving away [19, 20, 22, 24, 37, 39, 45], lack of interest or time [21, 24, 36] and transportation issues [29, 31]. Two studies also identified lack of support from family and friends [74] and hospital anxiety [19] as barriers to completion.

#### Adverse events

Only six studies included in this review explicitly discussed the occurrence of AEs. Of these, most studies (5 out of 6) found that the intervention did not affect the patients’ clinical presentation and symptoms. Sagen et al. [39] reported two cases of adhesive capsulitis and one case of supraspinatus tendinopathy. However, the timing of these AEs was not specified, therefore it is unclear whether these are due to the rehabilitation interventions or related to breast cancer treatments. A significant proportion of studies (25 out of 57) also reported that some participants suffered postoperative complications. Among these, lymphedema, seroma, wound dehiscence, and scar contracture were the most frequent. Once again, with little or no description of when these complications occurred, it remains unclear whether these were acute or late effects of breast cancer treatments.

## Discussion

This scoping review examined the extent and nature of clinical research on perioperative physical rehabilitation for women with breast cancer who were awaiting or had undergone mastectomy. Our main objective was to identify conservative interventions and relevant clinical outcome measures currently used for this population. As a secondary objective, we aimed to report on barriers and facilitators of participating and completing these interventions. Over half of the eligible studies included mixed breast cancer stages (0-III) populations who underwent various types of breast surgery, axillary procedures, and a series of adjuvant treatments.

### Conservative interventions

Rehabilitation programs identified four main modalities: exercise, patient education, manual therapy, and MLD. Multimodal rehabilitation interventions were most frequently reported, all of which included exercise. Rehabilitation interventions consisted primarily of one-on-one sessions initially performed under supervision in hospital settings until discharge. This review also established that rehabilitation interventions were by far the most studied after breast surgery. Only ten interventions were initiated preoperatively, consisting primarily of self-management strategies to be implemented in the postoperative period. Most interventions lasted less than 6 months.

The rehabilitation interventions identified in this scoping review reflect, to some extent, the recommendations provided by cancer care guidelines. However, we noted that the eligible studies had placed less emphasis on aerobic training, primarily providing rehabilitation programs that included exercises targeting upper extremity function. Few identified recommendations concerning rehabilitation strategies to be implemented before surgery, either in the eligible studies or in cancer care guidelines, indicating that further research is needed in this area. In 2017, the World Health Organization (WHO) urged for a coordinated and concerted global action toward improving the accessibility of high-quality rehabilitation services in health systems. Given the systemic effects of cancer and its associated treatments, oncology was designated as a priority area for this initiative [77]. Accordingly, a systematic review was conducted to identify and synthesize rehabilitation-specific recommendations provided by the most recent cancer care guidelines [78]. Of these, the American Cancer Society (ACS)/American Society for Clinical Oncology (ASCO) guideline [79] concluded that there was insufficient evidence to support a specific intervention that would promote optimal postoperative recovery for breast cancer patients. Nevertheless, physical rehabilitation recommendations endorsed by this guideline advised clinicians to encourage their patients to adhere to the ACS’s physical activity recommendations [80], which include moderate to vigorous aerobic exercises and strength training. Returning to normal daily activities as soon as possible after diagnosis and including spouses and family members in usual breast cancer care were also promoted. In turn, to manage breast cancer patients with or at risk for lymphedema, the National Comprehensive Cancer Network Survivorship Guideline [81] recommended a supervised multimodal rehabilitation intervention consisting of progressive resistance training, shoulder ROM exercises, manual lymphatic drainage, education regarding signs and symptoms of postoperative complications and self-care management strategies. This multimodal strategy is also consistent with the recommendations issued from the American College of Sports Medicine guideline [82], which supported the effectiveness of combined moderate-intensity aerobic and progressive resistance training, performed for 8 to 12 weeks, in improving cancer-related health outcomes, including physical functioning, QoL and fatigue. Interestingly, none of these recommendations provided guidance as to what parameters (i.e., frequency, repetitions, sets, etc.) should characterize shoulder ROM exercises. It should also be stressed that these guidelines were primarily derived from studies performed on breast cancer survivors. Therefore, these recommendations may not be fully applicable to breast cancer patients dealing with the acute effects of mastectomy.

### Clinical outcome measures

A significant number of outcome measures were used to report the effects of perioperative rehabilitation in breast cancer patients, each of which was measured through a wide range of questionnaires and measurement tools. Objective measures of physical function were the most frequently used and combined with PROMS in over half of the eligible studies. Considering the large spectrum of side effects of breast cancer and its treatments, selecting relevant clinical outcome measures for this population can be challenging. The WHO’s International Classification of Functioning, Disability and Health (ICF) is a common framework that describes health and disability worldwide [83]. As the ICF was considered hardly practical for research and clinical practice, the WHO developed core sets from this classification, which are lists of predetermined outcome measures known to be relevant for specific health conditions [83]. The ICF Core Set for breast cancer [84] covers all the factors that may impact breast cancer patients’ functioning. This model acknowledges that breast cancer patients may experience disabilities not only related to (1) body structures and (2) functions, but also in relation to (3) activities participation and (4) environment interaction [84]. Most studies (39 out of 57) included in this review used outcome measures belonging to at least 2 of the 4 categories of the ICF core sets for breast cancer. Objective measures of physical function were used extensively to account for items pertaining to the first two categories. In contrast, QoL questionnaires were mostly used to report on patients’ ability to carry out activities of daily living and interact with their environment. As QoL is a construct that encompasses many dimensions, the data obtained from these questionnaires may not be as informative. For psychological, social, and environmental factors to be adequately measured, it is advisable to select tools that can provide individual scores for these domains. As an example, the Functional Assessment of Cancer Therapy-Breast Questionnaire (FACT-B) is a questionnaire designed to measure five domains of health-related QoL in breast cancer patients: physical, social, emotional, functional well-being as well as breast cancer-specific concerns [85].

### Patients’ experience

This literature review also revealed that a variable proportion of breast cancer patients refused to engage in a rehabilitation intervention despite their eligibility. Studies identified a significant discrepancy in refusal rates. When comparing studies with higher refusal rates to those with lower rates, we noted that these studies had similar characteristics in terms of population, type of interventions, duration, and postoperative complications. However, most studies with higher refusal rates appeared to be conducted partly or entirely in hospital settings. As some wanted to minimize hospital appointments in favor of returning to a normal lifestyle, this information might suggest that transitioning from a supervised inpatient to a home-based intervention or implementing rehabilitation interventions in outpatient clinics or community settings may promote patient engagement. Study withdrawals were mainly attributed to personal or treatment-related factors rather than the intervention itself, which seems to support the appropriateness and safety of rehabilitation interventions for this population. Recognizing the positive impact that support from family and friends had on participants' motivation raises the possibility that breast cancer patients could also benefit from a group intervention, where they could support each other as they go through the same challenges. Tailoring interventions to participants’ needs and circumstances also appears to promote intervention compliance. However, given the small number of studies from which these data were obtained, further work is needed to better document these issues.

### Reporting of interventions and outcome measures

We identified several gaps in interventions and harm reporting by relying on the revised CONSORT statement and extensions [76, 86, 87] to guide data extraction. As shown in Additional file 2: Tables S1 and S2, these limitations are such that it remains unclear which parameters should be preferred to promote optimal postoperative recovery in breast cancer patients. Improvements in reporting are needed to ensure patient safety and replicability of interventions in clinical settings. A better description of recruitment and compliance issues arising in this clinical context is also warranted to foster the development of interventions tailored to breast cancer patients’ needs and concerns. As for clinical outcome measures, several studies have used measurement tools and questionnaires without mentioning their validity for the population of interest. To ensure the effects of rehabilitation interventions are accurately measured, future studies should focus on better describing these tools while providing evidence supporting their validity for breast cancer patients.

### Limitations

Our scoping review has some limitations. Despite conducting robust systematic searches in multiple relevant databases, we excluded studies not published in English or French (authors’ native language), which may have resulted in relevant studies being missed. However, it has been reported that excluding non-English publications from evidence-syntheses does not lead to bias as it would have a minimal effect on overall conclusions [88, 89]. Some studies were also excluded as they focused on breast cancer survivors. However, some organizations, such as the National Cancer Institute, identify cancer patients as survivors from the day of their diagnosis until the end of their lives [90]. Therefore, studies that did not provide a clear definition of survivorship may have been excluded despite their eligibility. We must also consider that conducting a mixed method scoping review, which would have included qualitative designs, would probably have been better suited to identify barriers and facilitators to study engagement and completion.

## Conclusion

This review reports on the variability and wide range of conservative interventions and clinical outcome measures used in physical rehabilitation for breast cancer patients undergoing mastectomy. Exercise, patient education, manual therapy, and MLD were identified as key components characterizing rehabilitation strategies for this population. Although most studies failed to describe interventions’ procedures and characteristics adequately, we were able to determine that most interventions were multimodal, initiated a few days following surgery, and initially performed in supervised hospital settings. More emphasis should be placed on selecting measurement tools and questionnaires that have already been validated for this population. Tailoring interventions to patients’ needs and promoting outpatient rehabilitation interventions appear to be better suited to the particularities of breast cancer care pathways. Ultimately, given the significant heterogeneity characterizing the interventions identified, a better understanding of breast cancer patients’ perioperative care needs and expectations is needed before we can work towards developing rehabilitation resources that can be embedded in our institutions' standards of care.

## Supplementary Information

**Additional file 1**. MEDLINE search strategy

**Additional file 2**. Summary of included studies and description of rehabilitation the interventions

## Data Availability

All data is contained within the manuscript and the additional file.

## Abbreviations

ACS: American Cancer Society
AES: Adverse events
ALND: Axillary lymph node dissection
ASCO: American Society of Clinical Oncology
BCS: Breast conserving surgery
CIUSSS-MCQ: Centre intégré universitaire de santé et de services sociaux de la Mauricie-et-du-Centre-du-Québec
FEV1: Forced expiratory volume in one second
FVC: Forced vital capacity
ICF: International Classification of Functioning, Disability and Health
MLD: Manual lymphatic drainage
PROMS: Patient-reported outcome measures
QoL: Quality of life
RCT: Randomized control trial
ROM: Range of motion
WHO: World Health Organization
