# Supplementary material for: Conservative interventions and clinical outcome measures used in the perioperative rehabilitation of breast cancer patients undergoing mastectomy: a scoping review
Source: BMC Womens Health. 2022 Aug 16;22:343. doi: 10.1186/s12905-022-01927-3 (PMC9380320; doi:10.1186/s12905-022-01927-3)
Supplement: Supplementary file 1 — Additional file 1. MEDLINE search strategy [file 12905_2022_1927_MOESM1_ESM.docx]

**Additional file 1**

Medline Search Strategy

1. Breast Neoplasms [Mesh – no exp]
2. Carcinoma, Ductal, Breast [Mesh]
3. Carcinoma, Lobular [Mesh]
4. Breast Carcinoma In Situ [Mesh -no exp]
5. [Carcinoma, Intraductal, Noninfiltrating](https://www.ncbi.nlm.nih.gov/mesh/68002285) [Mesh]
6. Unilateral Breast Neoplasms [Mesh]
7. Triple Negative Breast Neoplasms [Mesh]
8. breast cancer* [Title/Abstract]
9. breast tumo*
10. breast carcinoma*
11. breast neoplasm*
12. lobular N2 (carcinoma* or neoplasm* or tumo* or cancer*)
13. ductal carcinoma*
14. intraductal carcinoma*
15. breast malignant neoplasm*
16. breast malignant tumo*
17. mammary carcinoma*
18. mammary cancer*
19. mammary tumo*
20. mammary neoplasm*
21. **1-20/ OR**
22. exp Mastectomy [Mesh]
23. mastectom*
24. mammectom*
25. postmastectom*
26. postmammectom*
27. simple mastectomy
28. total mastectomy
29. extended simple mastectomy
30. radical-mastectomy
31. modified radical-mastectomy
32. prophylactic-mastectomy
33. risk-reducing (surgery or mastectomy)
34. preventive mastectomy
35. contralateral mastectomy
36. bilateral mastectomy
37. breast-conserving (surgery or therapy)
38. partial (mastectomy or mammectomy)
39. lumpectomy
40. postlumpectomy
41. segmental (mastectomy or mammectomy)
42. breast segmentectomy
43. quadrantectomy
44. breast tumorectomy
45. breast tumo* (resection or excision)
46. wide local excision
47. limited resection (mastectomy or mammectomy)
48. local excision (mastectomy or mammectomy)
49. sector* resection
50. conservative breast cancer treatment
51. partial-breast treatment
52. breast conservation therapy
53. nipple-sparing-mastectomy
54. aerola-sparing-mastectomy
55. breast-sparing (mastectomy or surgery)
56. subcutaneous mastectomy
57. **22-56/OR**
58. Rehabilitation [Mesh- no exp]
59. Activities of Daily Living [Mesh]
60. Range of Motion, Articular [Mesh]
61. exp Exercise Therapy [Mesh]
62. Physical Therapy Modalities [Mesh- no exp]
63. Complementary Therapies [Mesh- no exp]
64. Tai Ji [Mesh]
65. Yoga [Mesh]
66. Musculoskeletal Manipulations [Mesh-no exp]
67. Manipulation, Chiropractic [Mesh]
68. Manipulation, Osteopathic [Mesh]
69. exp Therapy, Soft Tissue [Mesh]
70. Conservative Treatment [Mesh]
71. exp Exercise Movement Techniques [Mesh]
72. exp Exercise [Mesh]
73. rehabilitation [Title/Abstract]
74. preoperative N2 (rehabilitation or exercise*)
75. postoperative N2 (rehabilitation or exercise*)
76. prehabilitation
77. activit* N3 (daily living or daily life)
78. exercise*
79. training
80. conditioning
81. stretching
82. physical activit*
83. range of motion
84. (shoulder or arm or upper limb or upper extremity) N3 (pain or morbidity or impairment*)
85. manipulation* or mobilization* or mobilisation* or massage or manual therap*
86. osteopath* or chiropractic or physiotherapy or kines*
87. conservative or nonsurgical
88. therap* N3 (exercise* or motion or soft tissue)
89. yoga
90. qigong
91. **58-91/ OR**
92. **21 AND 57 AND 91**
