# Supplementary material for: Conservative interventions and clinical outcome measures used in the perioperative rehabilitation of breast cancer patients undergoing mastectomy: a scoping review
Source: BMC Womens Health. 2022 Aug 16;22:343. doi: 10.1186/s12905-022-01927-3 (PMC9380320; doi:10.1186/s12905-022-01927-3)
Supplement: Supplementary file 2 — Additional file 2. Summary of included studies and description of rehabilitation the interventions [file 12905_2022_1927_MOESM2_ESM.docx]

**Additional file 2**

Supplementary Table 1: Evidence Table

| **First author**  **(Year)**  **Country** | **Study design** | **Participants** | **Intervention** | **Outcome measures** | **Outcome validation information** | **Patients’ experience** |
| --- | --- | --- | --- | --- | --- | --- |
| Ammitzbøll  (2019)  Denmark | RCT | N= 158  **Exercise IG; n= 82**  *Age, mean ± SD*: 53 ± 10 *Stage, n (%):*  I: 12 (15)/II: 48 (59)/III: 15(18)/ N/A: 7 (9)  *Sx type, n (%):*  LUMP +ALDN: 43(52)/  MX + ALDN: 39(48)  *Systemic treatment, n (%)*  RT: 82 (100)/Adj Ch: 48 (59)  Neoadj Ch: 25 (30)/HT: 64 (78)  **Usual-care CG; n = 76**  *Stage, n (%):*  I: 16 (21)/II: 35 (46)/III: 18 (24)/N/A: 7 (9)  *Sx type, n (%):*  LUMP + ALDN: 41(54) MX +ALDN: 35(46)  *Systemic treatment, n (%)*  RT: 82 (100)/Adj Ch: 45 (59)  Neoadj Ch: 21 (28)/HT: 51 (67) | **Exercise intervention group**  Resistance exercises program (covered all major muscles groups of the UL and lower limbs, and core strength and stability)  *Phase* 1 (w1-w20)  I*nitiation*: 3^rd^ post-op w  F*requency*: 3 days/w (supervised in group: 2/w; self-A: 1/w)  *Phase 2 (w21-w50)*  *Initiation*: after phase 1  *Frequency*: 3 days/w (self-A exercises solely)  *Exercise sessions duration*:50-55 min (10-15-min warm-up, 40 min of resistance training)  *Rep/Load/Sets:*  w1-4 :15-20/25 RM/2-3 sets  w5-8: 15-17/20 RM/3 sets  w9-12: 10-12/15 RM/3 sets  w13-50: 10-12/10-12 RM/3 sets  **Usual-care control group**  No intervention provided but were allowed to participate in municipality-led rehabilitation programs without restrictions  *Follow up:*  12 months | -Arm VOL*-ILVD* (water displacement)  LE-related symptoms: heaviness, tightness and swelling  (NRS-11)  -Muscle strength (7RM-test and dynamometer)  -Shoulder movement (goniometer)  -Interlimb mass difference-*ILMD* (DXA and arm scan)  -Clinical examination- LE  (Stanton & al. criteria)  -Clinically relevant LE (> 3% increased ILVD, NRS-11 ≥ 2 and 2 or more clinical criteria) | *BC-specific outcomes:*  Water displacement  *General*:  DXA  *Undisclosed*:  NRS-11  ILMD  Clinical criteria  7RM-test  Goniometer | Intervention group:  17 participants experienced an increased in symptoms during phase 1  *Lost to follow-up*  Intervention group: hospital anxiety (n=1), cancer recurrence (n=1), deaths (n=3)  Control group: pain due to scar tissue (n=1), recent breast recons (n=1), no time or resources (n=2), cancer recurrence (n=3) |
| Ammitzbøll  (2019)  Denmark | RCT | N= 158  **Exercise IG; n= 82**  *Age, mean ± SD*: 53 ± 10 *Stage, n (%):* I: 12 (15)/II: 48 (59)/III: 15 (18) N/A: 7 (9)  *Sx type, n (%):*  LUMP +ALDN: 43(52)/MX + ALDN: 39(48)  *Systemic treatment, n (%)*  RT: 82 (100)/Adj Ch: 48 (59)  Neoadj Ch: 25 (30)/HT: 64 (78)  **Usual-care CG; n = 76**  *Stage, n (%):*  I: 16 (21)/II: 35 (46)/III: 18 (24)/N/A: 7 (9)  *Sx type, n (%):*  LUMP + ALDN: 41(54)/MX +ALDN: 35(46)  *Systemic treatment, n (%)*  RT: 82 (100)/Adj Ch: 45 (59)  Neoadj Ch: 21 (28)/HT: 51 (67) | **Exercise intervention group**  Resistance exercises program (covered all major muscles groups of the UL and lower limbs, and core strength and stability)  *Phase* 1 (w1-w20)  *Initiation*: 3^rd^ post-op w  *Frequency:* 20 w; 3 days/w (supervised in group: 2/w; self-A: 1/w)  *Phase 2 (w21-w50)*  *Initiation:* after phase 1  *Duration*: 30 w; 3 days/w  (self-A exercises)  *Exercise sessions duration:* 50-55 min (10-15-min warm-up, 40 resistance training)  *Rep/Load/Sets:*  w1-4 :15-20/25 RM/2-3 sets  w5-8: 15-17/20 RM/3 sets  w9-12: 10-12/15 RM/3 sets  w13-50: 10-12/10-12 RM/3 sets  **Usual-care control group**  No intervention provided but were allowed to participate in municipality-based PT (heterogeneous offers of MT and mobility exercises)  *Follow up:*  20w, 12 months | -HRQOL  (EORTC QLQ C-30 v3; FACIT-f)  -Symptom clustered: pain-sleep-fatigue  (EORTC QLQ-C30 v3) | *Cancer-specific outcomes:*  EORTC QLQ C-30  FACIT-f  *Undisclosed:*  Symptom clustered | *Lost to follow-up:*  IG: death (n=3),  logistics (n=3), unable to contact (n=1), hospital anxiety (n=1), personal reasons (n=1) and cancer recurrence (n=1)  CG: unable to contact (n=3), other Sx (n=1), no time (n=2), personal reasons (n=3), withdrew consent (n=1), pain from scar (n=1), cancer recurrence (n=3) |
| Anderson  (2012)  USA | RCT | N= 104  **Intervention arm; n= 52**  *Age group, n (%):*  *< 50:* 21 (40)/*50-64:* 23 (44)  *65-74:* 4 (8)/ *>75:* 4 (8)  *Stage, n (%):*  I: 25 (48)/II: 19 (37)/III: 8 (15)/N/A: 1(2)  *Sx type, n (%):* LUMP: 23 (44)/MX: 28 (54)/ N/A: 1 (2)  *Type of node dissection, n (%):*  SND only: 10 (19)/AND: 39 (75)  Neither: 1 (2)/N/A: 2 (4)  *Systemic treatment, n (%):*  *Ch:* 31(60)/*HT:* 26(50)/*RT:* 31(60)  **Comparison arm; n=52**  *Age group, n (%)*  *< 50:* 23 (44)/*50-64:* 19 (37)  *65-74:* 7 (13)/ *>75:* 3 (6)  *Stage, n (%):*  I: 26 (50)/II: 21 (40)/III: 4 (8)/N/A: 1(2)  *Sx type, n (%):*  LUMP: 25(48)/MX: 24(46)/N/A: 3(6)  *Type of node dissection, n (%):*  SND only: 9 (17)/ALND: 40 (77) Neither: 0/ N/A: 3 (6)  *Systemic treatment, n (%):*  Ch: 31(60)/HT: 23(44)/RT: 36(69) | **Intervention arm**  Comprehensive program consisting of tailored exercises and LE prevention  *Initiation*: 4-12w post-op  *LE prevention module (LPM)*  Included instructions and care for the affected arm and hand, video-taped tutorial of arm strengthening and lymph flow exercises and instructions to wear a compression sleeve  *Tailored exercise program*  Included an aerobic warm-up, moderate to hard walking (RPE scale), upper and lower body strength training and stretching exercises  *Initiation*: following initiation of LPM  *Intensive phase (m1-3)*  Frequency: 2 days/w (supervised exercises)  *Phase 2 (m4-6)*  Transition to HB exercises (supervised exercises 1 day/w)  *Phase 3 (m7-12)*  HB exercises (supervised exercises not mandatory)  *Exercise sessions duration*: 65 min (5-min warm-up, 30-min of walking, 20 min of strengthening exercises and 10 min of stretching)  *Load/Repetitions*:  50% 1 RM /up to 12 repetitions (weights were increased weekly)  **Comparison arm**  Usual care consisting of patient ED (LE awareness, tips about PA and nutrition, recommendations for improving function and strength)  *Follow up:*  6, 9, 12 and 18 months post-Sx | Arm VOL  (Water displacement)  -Function  (6MWT)  -HRQOL  (FACT-B) | *BC-specific outcomes:*  Water displacement  FACT-B  *Cancer-specific outcomes*  6MWT | Reasons stated for not completing  the study:  -Feeling overwhelmed or lack of time to  participate (38%)  -Lost to follow-up (19%)  -Lack of interest  (10%)  -Family issues (10%)  -Death (10%)  -Other reasons (10%)  Adherence: Participants completing 71.2% of all prescribed exercise sessions with a range of 0–97%. Majority (61%) of participants attending more than 75% of prescribed sessions and only 13% of participants attending less than 50% of sessions |
| Bendz. (2002)  Sweden | RCT | N= 230  **Group A; n= 115**  *Age, mean ± SD*: 58 ± 11  *Stage*: N/A  *Sx type, n (%):*  MX: 31 (31) /MX + RT: 5 (5)  QT: 20 (20) / QT + RT: 45 (44)  **Group B; n = 115**  *Age, mean ± SD*: 58 ± 11  *Stage:* N/A  *Sx type, n (%):*  MX: 22 (21) / MX + RT: 7 (7)  QT: 23 (22) /QT + RT: 52 (50) | **Group A**  Early shoulder exercises (to be started on the 1^st^ post-op day)  Day 1-13: Early shoulder exercises including intermittent hand contractions and basic ROM exercises  From Day 14: Comprehensive ROM exercise program  **Group B; n = 115**  Delayed shoulder exercises  Preoperative instructions: were advised to use the arm as much as comfortable but to avoid lifting and carrying heavier items  Day 1-13: No further information was provided  From Day 14: Comprehensive ROM exercise program  Patients were told to perform each exercise 5 times in every set and repeat the session 3 times daily.  *Follow up:* 2w, 1 month, 6 months, 2 years | Arm VOL  (water displacement)  Shoulder ROM  (Myrin goniometer)  Grip strength  (vigorimeter)  Patient-reported measures of pain, heaviness and tension  (VAS scale) | *BC-specific outcomes:*  Water displacement  *Disclosed for general population:*  Myrin Goniometer  Vigorimeter  VAS scale | During the study period 25 patients  dropped out. The causes were: death (five patients),  moving from the area (six patients), other diseases (three  patients), Sx on the opposite side (three patients)  and personal reasons (eight patients). |
| Beurskens  (2007)  Netherlands | RCT | N= 30  **Physiotherapy group; n= 15**  *Age, mean ± SD*: 53.7 ± 13.0  *Stage:* N/A  *Sx type, n (%):*  BCS + ALDN: 3 (20)/MX+ ALDN: 12 (60)  *Systemic treatment, n (%):*  Ch: 2 (13)/HT: 1 (7)/RT+ Ch: 6 (40)  Ch+ HT: 1 (7)/RT+ HT: 1 (7)  RT + HT + Ch: 1 (7)  **Control group; n=15**  *Age, mean ± SD*: 55.4 ± 9.3  *Stage*: N/A  *Sx type, n (%):*  BCS + ALDN: 4 (27)/MX + ALDN: 11 (73)  *Systemic treatment, n (%):*  RT: 2 (13)/Ch: 2 (13)/HT: 1 (7)  RT+ Ch: 8 (53)/Ch + HT: 1 (7)  RT + HT: 1 (7) | **Physiotherapy group**  PT sessions in a private office of their own choice. Enrolled PTs received information about treatment guidelines: advice and exercises for arm/shoulder, posture correction, coordination exercises, exercises for muscular strength, improvement of general physical condition, exercises to prevent LE and instruction for soft tissue massage of the scar if required  *Initiation*: 2w following Sx  *Duration*: 3 months  *Frequency*: 1-2/w for the first 3w and then once a fortnight or less + 10 min of home exercises daily  **Control group**  Leaflet flyer with advice and exercises for the arm/shoulder and had no further contact with the physiotherapist  *Initiation*: 1^st^ w following Sx | Arm/shoulder pain  (VAS)  Shoulder mobility  (digital inclinometer)  Disabilities in daily life  (DASH)  Arm edema  (water displacement)  Grip strength  (hand-held dynamometer)  Quality of life  (SIP questionnaire short version) | *Disclosed for general population*  DASH  SIP questionnaire  *Undisclosed*  VAS  Digital inclinometer  Water displacement  Hand-held dynamometer | Thirty women completed the study protocol. In the follow-up period one patient from the control group died before the last assessment |
| Box  (2002)  Australia | RCT | N= 65  **Treatment group; n= 33**  *Age, mean ± SD*: 53.03 ± 9.49  Stage: N/A  *Sx type (%):* BCS + ALND: 46.9/ MRM: 53.1  **Control group; n = 32**  *Age, mean ± SD*: 59.00 ± 10.95  Stage: N/A  *Sx type (%):* BCS + ALND: 51.5/MRM: 48.5 | **Treatment group**  *Physiotherapy Management Care Plan (PMCP)*  Included a thorough preop  assessment and explanation with postop reviews to monitor shoulder ROM, progress exercise program, LE awareness ED and individualized intervention as required.  **Control group**  Exercise instruction booklet  *Follow up*  Day 5, 1 month, 3, 6, 12 and 24 months postoperatively | Shoulder ROM  (goniometer)  Function  (12-items functional questionnaire) | *Undisclosed* | Dropouts (9%): relocation interstate or overseas (2), unavailability for final assessment (1) and death (3).  Both groups: reduction in their compliance with  the prescribed exercise protocol from the 1 m. review onwards  It is possible that the greater compliance of the TG women over the first 12 m may have been due to the PTs reinforcement of the benefits of the continued exercise  programme. |
| Box  (2002)  Australia | RCT | N= 65  **Treatment group; n= 33**  *Age, mean ± SD*: 53.03 ± 9.49  *Stage*: N/A  *Sx type (%):* BCS + ALND: 46.9/ MRM: 53.1  **Control group; n = 32**  *Age, mean ± SD*: 59.00 ± 10.95  *Stage*: N/A  *Sx type* (%): BCS + ALND: 51.5/MRM: 48.5 | **Treatment group; n = 33**  *Physiotherapy Management Care Plan (PMCP)*  Included a thorough preop  assessment and explanation with postop reviews to monitor shoulder ROM, progress exercise program, LE awareness ED and individualized intervention as required.  **Control group; n = 32**  Exercise instruction booklet  *Follow up*  Day 5, 1 month, 3, 6, 12 and 24 months postoperatively | Arm size- *CIRC*  Arm VOL  (water displacement)  Multi-frequency bioimpedance-*MFBIA* (spectroscopy)  Incidence of secondary LE  (based on preop CIRC, preop VOL and MFBIA ratio) | Disclosed but unable to retrieve the study | In this current study, 28% of women (n=16/57) reported that they felt that their arm was swollen at 24 m. More than 50% of these women reported subjectively that  the swelling was in the UA but that it occurred  intermittently and could not always be associated with a precipitating event |
| Cho  (2016)  South Korea | RCT | N= 48  BC patients with AWS  **PTMLD group; n= 24**  *Age, mean ± SD*: 50.7 ± 9.6  *Stage, n (%):*  I: 5 (24)/III: 16 (76)  *Sx type, n (%):* MX: 12 (57)/ LUMP: 7 (33)  Breast recons: 2 (10)  *Systemic treatment, n (%):*  *Ch:* 9 (43)/*RT:* 21(100)/*HT:* 14 (67)  **PT group; n = 24**  *Age, mean ± SD*: 46.6 ± 6.8  *Stage, n (%):*  I: 12 (60)/III: 8 (40)  *Sx type, n (%):* MX: 16 (80) / LUMP: 3 (15)  Breast recons: 1 (5)  *Systemic treatment, n (%):*  *Ch:* 11 (55)/*RT:* 19(95)/*HT:* 12 (60) | **PTMLD group**  Underwent a PT program combined with MLD  Initiation: At least 4w after BSx  *Supervised PT program*  UE strengthening and stretching exercises combined with MT session (ST mobs and stretching, shoulder stretching exercises, shoulder girdle mobs and PROM exercises  Duration: 4w  Frequency: 3 times/w  Repetitions/Sets/Int :10/3/60-80% 1 RM  *MLD*  Frequency: 5 days/w for 4w  MLD sessions duration: 30 min  *w1*: performed by 2 certified PT  *w2-4*: self-A  **PT group; n = 24**  PT program solely | Arm VOL (CIRC tape measurements)  Muscular strength (dynamometer)  Active ROM  (inclinometer)  Pain  (NRS-11)  Arm disability  (DASH)  QoL  (EORTC QLQ-C30 v3, EORTC QLQ-BR23)  Visible cording  (Subjective assessment by a rehab doctor) | *BC-specific outcomes:*  CIRC tape measurements  EORTC QLQ-BR23  *Cancer-specific outcomes:*  EORTC QLQ-C30 v3  *Disclosed for general population:*  Dynamometer  DASH  NRS-11  *Undisclosed:*  Inclinometer | Six of the 20 patients in the PT group developed LE after 4 weeks.  Seven patients were unable to complete the final evaluation: therefore, only 41 patients completed the study |
| Cinar  (2008)  Turkey | RCT | N = 57  **Treatment group; n = 27**  *Age, mean ± SD*: 51.1 ± 13.0  *Stage, n (%):* N/A  *Sx type*: *MRM*  *Systemic treatment, n (%):*  Ch: 29 (97)/RT: 14 (47)  **HB exercise program; n= 30**  *Age, mean ± SD*: 52.6 ± 12.2  *Stage, n (%):* N/A  *Sx type*: *MRM*  *Systemic treatment, n (%):* Ch:23(85)/RT:10(4) | **Treatment group**  Early shoulder ROM exercises (to be started on the 1^st^ post-op day) and PT program  *Day1:* AROM exercises supervised by a PT  *Day2*: Isometric hand and forearm exercises  *Day3-4*: Active assistive and AROM exercises of the shoulder joint  *PT program*  Included ROM, stretching and strengthening exercises  Initiation: Following drains removal  Duration: 15 supervised sessions and 8w self-A  **Home-based exercise program group**  Postoperative exercise forms to perform at home  *Follow up*  Day 5, 1 month, 3 and 6 months postoperatively | ROM  (Myrin goniometer)  Arm VOL  (CIRC tape measurements)  Function  (10-item functional questionnaire) | *Undisclosed:*  CIRC tape measurements  Functional questionnaire  Myrin Goniometer | No statistically  significant difference in postoperative complication like  seroma and wound infections. None of the patients had  postoperative hematome  Early rehabilitation started on the first postoperative day did not have an adverse effect  on local infection, hematoma, and seroma formation and did not cause an increase in duration and amount of LD |
| De Groef  (2017)  Belgium | RCT | N= 147  **Intervention group; n= 72**  *Age, mean ± SD* :53.9 ± 11.5  *Stage, n (%):*  0: 7 (10)/ I: 16 (22) /II: 36 (50)/ III: 13 (18) IV: 0 (0)  *Sx type, n (%):* MX: 46 (64)/ BCS: 26 (36)  *Systemic treatment, n (%):*  RT, IMC and medial supraclavicular: 72(100)  RT, axilla: 8(11)/Ch: 60(83)/Neoadj Ch: 29(40)  Target therapy: 22(31)/HT: 57(79)  **Control group; n = 75**  *Age, mean ± SD*: 54.7 ± 11.9  *Stage, n (%):*0: 2 (3)/ I: 20 (27)/ II: 37(48)/ III:14(19)/ IV:2 (3)  *Sx type, n (%):* MX: 50 (67)/ BCS: 25 (33)  *Systemic treatment, n (%):*  RT, IMC and medial supraclavicular: 75 (100)  RT, axilla: 9(12)/Ch:55(73)/Neoadj Ch:21(28)  Target therapy: 9 (12)/HT: 62 (83) | **Both groups**  Individual standard physical therapy program consisting of different PT modalities: passive mobs to improve PROM and AROM, stretching and transverse strain of pectoral muscles, scar tissue massage, exercises schemes to restore and improve muscle flexibility, endurance and strength, posture and movement control and active shoulder ROM  Initiation: immediately after Sx  Duration: 4 months  Exercise sessions duration: 30 min  Frequency: 2 session/w, reducing to once/w after the first 2 months  **Intervention group**  Individual standard physical therapy program + myofascial therapy  Initiation: 2 months post-Sx  Duration (MT): 2 months  Frequency of MT sessions: once/w  **Control group**  Individual standard physical therapy program + placebo treatment consisting of static bilateral hand treatment at the upper body and arm  Initiation: 2 months post-Sx  Duration (placebo): 2 months  Frequency: once/w  Placebo treatments duration: 30 min  *Follow-up*  1w, 2, 4, 9 and 12 months post-Sx | Point prevalence of pain  (Yes/No question)  Pain intensity  (VAS)  Pressure hypersensitivity  (digital Wagner FPX algometer)  Pain quality  (McGill Pain Questionnaire)  Point prevalence of impaired shoulder function  (DASH score of more than 15%)  Shoulder function  (DASH)  Quality of life  (SF-36) | *BC-specific outcome*  SF-36  *Disclosed for general population*  Pressure algometer  McGill Pain Questionnaire  DASH  *Undisclosed*  VAS | *Dropouts*  Intervention group: not able to come to the hospital (n=3)  Control group:  No dropouts |
| De Rezende  (2006)  Brazil | RCT | N= 60  **Directed exercises group; n = 30**  *Age, mean ± SD*: 54.00 ± 10.11  *Stage, n (%):*  I: 5 (17)/ IIA: 4 (13)/ IIB: 5 (16) IIIA: 4 (13)/ IIIB: 8 (27)/ IIIC:2 (7)/ IV: 2 (7)  *Sx type, n (%):*  Halsted RMX: 5(17)/MRM:16 (53)/ QT: 9 (30)  *Systemic treatment, n (%):*  Previous Ch: 8 (27)  **Free exercises group; n = 30**  *Age, mean ± SD*: 55.40 ± 11.24  *Stage, n (%):*  I: 6 (20)/ IIA: 10 (33)/ IIB: 6 (20)  IIIA: 3 (10)/ IIIB: 3 (10)/ IIIC: 0 (0)/IV: 2 (7)  *Sx type, n (%):*  Halsted RMX: 1 (3)/ MRM: 21 (70)/QT:8 (27)  *Systemic treatment, n (%):*  Previous Ch: 9 (30) | **Directed exercises group**  19 ROM-exercises program performed in groups of 5 to 20 women and supervised by a team of PT and students  Initiation: 1^st^ post-op day  Duration: 3 days/w for 42 days  Exercise sessions duration: 40 min  Repetitions: 10 rep each exercise, with a 60-s interval between exercises  **Free exercises group**  Same ROM exercises program without a previously defined sequence or number of repetitions | Shoulder ROM  (Manual goniometer)  Lymphatic disturbance  (Drainage VOL)  Arm CIRC  (Tape measure) | *Undisclosed:*  Manual goniometer  Drainage VOL  Tape measure of arm CIRC | The groups were homogeneous with respect to the  number of physiotherapy sessions performed, with  13.83 ± 3.05 sessions in the directed group and 13.19 ±  1.9 sessions in the free group. |
| Devoogdt  (2018)  Belgium | RCT | N= 160  **Experimental group; n = 79**  *Age, mean ± SD*: 56 ± 13  *Stage, n (%):*  0: 1(1)/ I:21(27)/II: 38(48)/III: 13(17)/ IV: 6 (8)  *Sx type, n (%):*  MX + ALDN: 52 (66)/BCS + ALDN: 27 (34)  *Systemic treatment, n (%):*  Ch: 50 (63)/Target therapy:14(18)/ HT: 55(70)  **Control group; n = 81**  *Age, mean ± SD:* 55 ± 11  *Stage, n (%):*  0: 0 (0)/ I: 26 (32)/ II: 39 (48)/III: 12 (15)/IV: 4 (5)  *Sx type, n (%):*  MX + ALDN: 56 (69)/BCS + ALDN: 25 (31)  *Systemic treatment, n (%):*  Ch: 58 (72)/ Target therapy: 7 (9)/HT: 66 (82) | **Both groups**  *During hospitalization:* Received information about the prevention of LE + exercise therapy (mobilizing exercises)  *After hospitalization:* 30-min individual exercise sessions were provided at the hospital, consisting of passive shoulder mobs, stretching of the breast muscles, scar tissue massage and active mobilizing and stabilizing exercises.  Duration: 6 months  Frequency: 2 times/ w, but gradually diminished to 1/ 2w  **Experimental group**  Protocol described above + MLD  Initiation: one week after removal of axillary drains  Duration of MLD: 20 weeks  Frequency of exercise sessions during this period: one to 3 times/ w and then gradually decreased to once/w  **Control group**  Protocol described above without MLD  *Follow-up:* 6, 12, 24 and 60 months post-Sx | Incidence of arm LE  (water displacement, arm CIRC)  Point prevalence of arm LE (water displacement, arm CIRC)  Point prevalence of subjective arm and trunk LE  (Questioned at interview)  Arm VOL difference  (Water displacement)  Shoulder ROM-abd, flexion, ext and int rotation  (Goniometer, tape measure)  Health-related QoL  (SF-36)  Problems in functioning  (Lymph-ICF) | *BC-specific outcome(s)*  Lymph-ICF  *Disclosed for general population*  SF-36  Water displacement  *Undisclosed*  Goniometer  Tape measurement  Arm CIRC | *Lost to follow-up:*  Exp: died (n=5), withdrew (n=6), unable to contact (n=3), cancer recurrence (n=1)  Control: died (n=4), withdrew (n=6), unable to contact (n=2), cancer recurrence (n=2)  The main  reason for absence during the therapy sessions was illness related  to Ch and/or RT. Other reasons were problems with transport, holiday, and illness from other causes. |
| Fatima  (2022)  Pakistan | RCT | N=30  **Treatment group; n=15**  **Control group; n=15**  *Overall mean age (y), mean ± SD:* 46 ± 10.75  *Sx type:* All participants were scheduled to undergo unilateral MRM and axillary clearance | **Treatment group**  Preoperative EX protocol  Initiation: Pre-operative period  Freq: 2-3 times/day, 2-5 sessions/w  REPS: ROM EX 10-12; stretching EX 8-10  Duration: Preoperative period and was repeated after the 1^st^ and 3^rd^ pod; continued with routine care in the postop period (unclear)  **Control group**  **Routine care (ROM EX)**  Initiation: Pre-operative period  Freq: 2-3 times/day  REPS: 10-12  Duration: unclear | Pain intensity  (NPRS)  Shoulder ROM  (Goniometer)  Ability to perform ADLs  (Groningen Activity Restriction Scale-GARS) | *Disclosed for another population:*  NPRS  GARS  *Disclosed for general population:*  Goniometer | - |
| Feyzioğlu (2020)  Turkey | RCT | N= 40  **Kinect-based rehabilitation group; n =20**  *Age, mean ± SD*: 50.84 ± 8.53  Stage, n (%): N/A  *Sx type*: Unilateral BSx + ALND  *Systemic treatment, n (%):*  Ch: 4 (21)/ RT: 13 (68)/ HT: 2 (11)  **Standardized physiotherapy group; n = 20**  *Age, mean ± SD*: 51.00 ± 7.06  *Stage, n (%):* N/A  *Sx type*: Unilateral BSx + ALND  *Systemic treatment, n (%):*  Ch: 2 (12)/ RT: 13 (77)/ HT: 2 (12) | **Both groups**  w0-2: Breathing, ROM and pumping exercises. Limitations for shoulder ROM amplitudes, weightlifting, jumping and running up to 6w post-op  Initiation: 1^st^ post-op day  Duration: 2w  **KBR group**  Xbox 360 Kinect video game program (requiring AROM of the UL) combined with tissue massage and passive mobs  Initiation: 2^nd^ post-op w  Duration: 2 days/w for 6 w  **SPT group**  Standard UE PT program (shoulder ROM, stretching and strengthening exercises) including scar tissue massage and mobs  Initiation: 2^nd^ post-op w  Duration: 2 days/w for 6 w  Program sessions duration (for both groups): 45 min | Pain intensity  (VAS)  Shoulder ROM  (Digital goniometer)  Shoulder muscle strength  (Handheld dynamometer)  Handgrip strength  (Hydraulic hand dynamometer)  Upper extremity function  (DASH)  Fear of movement  (TKS) | *BC-specific outcome(s):*  DASH  *Disclosed for general population:*  VAS  Handheld dynamometer  *Undisclosed:*  Digital goniometer  Hydraulic hand dynamometer  TKS | Dropouts:  *KBR group* (n=1)  Declined to participate  *SPT group* (n =3)  New metastasis focus (n=1)  Declined to participate (n =1)  Ch side effect  (n =1)  No complications occurred during Xbox Kinect VR training performed early in the postop period after BSx. The patients in the KBR group participated more motivationally and had less fear of movement during the entire program |
| Heiman  (2021)  Sweden | RCT | N= 400  **Intervention group; n=200**  *Age (median; i.g.r; range):* 61 (52-68; 30-84)  *Stage, n (%):* I: 92 (51.4)/ II: 83 (46.4)/ III: 4 (2.2)  *Sx type, n (%):* BCS: 147 (80.3)/ MX: 36 (19.7)  *Type of node dissection, n (%):*  SNB: 161 (88)/ ALND: 11 (6.0)  **Control group; n=200**  *Age (median; i.g.r; range):* 63 (54-71; 38-89)  *Stage, n (%):* I: 75 (38.7)/ II: 108 (55.7)/ III: 10 (5.2)/ IV: 1 (0.5)  *Sx type, n (%):* BCS: 154 (78.2)/ MX:43 (21.8)  *Type of node dissection, n (%):* SNB: 176 (89.3)/ ALND: 9 (4.6) | **Intervention group**  Instructions by a PT to add 30 min of aerobic PA daily + 2 follow-up calls  Initiation: 1-3w before Sx  Duration: up to 4w after discharge from hospital  **Control group**  Routine care (did not receive any advice regarding PA)  **Both groups**  Received standardized information from a PT regarding early mobs and shoulder movement before hospital discharge | Physical recovery  (self-reported questionnaires, SGPALS)  Mental recovery  (self-reported questionnaire)  Duration of hospital stay  Unplanned reoperations and readmissions  (retrieved from medical records)  Postoperative complications  (CCI) | *Disclosed for another population:*  SGPALS  *Undisclosed*:  Physical and mental recovery self-reported questionnaires  CCI | Complications developed within 30d of Sx in 15.6% in the IG compared  with 18.4 % in the CG.  Grade III complications  were documented in five patients in the CG and  one in the IG  Complications were seen within 90d of Sx in 23.5 % in the IG compared with 24.9% in the CG. One patient in the CG had one grade IV complication: grade III complications  were recorded for five patients in the CG and two in the IG |
| Joo  (2021)  Korea | RCT | N=56  **Early shoulder exercise group; n=28**  *Age, mean ± SD*: 44.50 ± 6.70  *Sx type, n (%):* MX + Immediate Brecons: 28 (100)  *Type of node dissection, n (%):* SNB: 26 (92.86)/ ALND: 2 (7.14)  **Arm restriction group; n=28**  *Age, mean ± SD*: 44.10 ± 8.35  *Sx type, n (%):* MX + Immediate Brecons: 28 (100)  *Type of node dissection, n (%):* SNB: 22 (78.57)/ ALND: 6 (21.43) | **Early shoulder exercise group**  Shoulder ROM exercise routine  Initiation: 2^nd^ pod  Duration: unclear  **Arm restriction group**  Any type of arm exercise was restricted until drains removal | Drainage volume  Duration of drain placement | - | *Early exercise group:* None of the patients suffered pain that required them to stop exercising.  No significance differences between groups regarding the total drainage volume and the duration of drainage maintenance period |
| Kilbreath  (2012)  Australia | RCT | N= 160  **Exercise group; n = 81**  *Age, mean ± SD*: 53.5 ± 12.1  Stage, %: I: 17 / II: 44 / III: 38  *Sx type, %:* MX + SNB: 48/ ALDN: 62  *Systemic treatment, %:* Ch: 68/ RT: 79  **Control group; n = 79**  *Age, mean ± SD*: 51.6 ± 11.0  *Stage, n (%):* I: 19/ II: 37/ III: 44  *Sx type, %:* MX: 47/ ALDN: 58  *Systemic treatment, %:* Ch: 71/ RT: 76 | **Both groups**  Postoperative care which included written information outlining postop arm exercises they were to perform, literature on prevention of LE  Initiation: 4-6w post-Sx  Duration: 8 w  **Exercise group**  Supervised exercise sessions of resistance training and passive stretching for shoulder muscles + home program of resistance training and stretching (positions maintained 5-15 min)  Initiation: 4-6 w post-Sx  Duration: 8w  Frequency: once/w  Sets/Reps (resistance training): 2/8-15  **Control group**  Were seen fortnightly to assess their arm for the presence of LE. No exercises or advice was provided. If LE was detected, patient was seen by an OT form at minimum, fitting of a compression garment  *Follow-up:* 6 months after the intervention | Self-reported arm symptoms  (EORTC- BR23)  Breast symptoms  (EORTC-BR23)  Shoulder ROM  (Digital inclinometer)  Upper shoulder muscle strength  (Hand-held dynamometer)  Presence of LE  (Bioimpedance spectroscopy) | *BC-specific outcome*  EORTC-BR23  Bioimpedance spectrometry  *Undisclosed*  Digital inclinometer  Hand-held dynamometer | Both groups reported little impairment including swelling  immediately following the intervention and at 6 months  post-intervention*.*  The median  number of sessions attended was 7 out of 8 sessions, with  adherence to the supervised training 78%.  Home program mean compliance: 90%. Seven women completed less than 75% of their stretching sessions and 4 women completed less than 75% of their resistance training sessions |
| Kilbreah  (2006)  Australia | RCT | N= 22  **Exercise group; n = 14**  *Age, mean ± SD*: 52.7 ± 14.0  *Stage*: N/A  *Sx type, n (%):* MX + ALDN: 8 (57)/ WLE + ALDN: 6 (43)  *Systemic treatment, n (%):* RT: 9(64)/Ch: 7(50)  **Control group; n = 8**  *Age, mean ± SD*: 51.5 ± 10.2  *Stage*: N/A  *Sx type, n (%):* MX + ALDN: 4 (50)/ WLE + ALDN: 4 (50)  *Systemic treatment, n (%):* RT:7(88)/ Ch:6 (75) | **Exercise group**  Usual care + shoulder ROM, strengthening and stretching exercises  Initiation: 4 to 5w post-Sx  Frequency: performed daily and supervised once/w by a PT  *Stretching exercises:* Was held passively for 5 min on day 1 and progressed up to 15 min over the next 2w  *Strengthening exercises:* Focused on shoulder flexors, abductors, and external rotators, a Theraband was used  Sets/Reps: 2/8-12  (Exercises were progressed by either increasing resistance or repetitions at subsequent weekly sessions)  **Control group**  Usual care (monitoring by a breast care nurse, may be seen by a PT to review UL exercises and by an OT who discussed prevention of LE) provided at the hospital, were discharged 2 to 7 days post-Sx  *Follow-up:* Following the 8-week intervention period | Quality of life  (EORTC-QLC-C30, EORTC-QLC-BR23)  Presence of LE  (arm CIRC measurements)  Shoulder ROM  (inclinometer)  Maximal isometric shoulder strength  (dynamometer) | *BC-specific outcome*  EORTC-QLC-BR23  Arm CIRC measurements  *Cancer-specific outcome*  EORTC-QLC-C30  *Undisclosed*  Inclinometer  Dynamometer | ‘’No adverse events were reported during the conduct of this study’’ |
| Klein  (2021)  Israel | RCT | N= 160  **Intervention group; n= 73**  *Age, mean ± SD*: 53.3 ± 12.7  *Stage, n (%):* IA: 40 (55.6)/ IB: 2 (2.8)/ IIA: 12 (16.7)/ IIB: 4 (5.6)/ IIIA: 0/IIIC: 1(1.4)  *Sx type, n (%): LUMP*: 4 (4.6); *LUMP + SNB:* 23 (31.9); *LUMP + ALND*: 7 (9.7); *PMMX + SNB*: 14 (19.4); *PMMX + ALND*: 1 (1.4); *PMMX + Brecons*: 23 (31.9)  *Systemic treatment, n (%):*  Neoadj Ch: 17 (23.6)/Adj Ch: 33 (45.8)/ RT: 51 (70.8)/ IORT: 8 (11.1)  **Control group; n= 87**  *Age, mean ± SD*: 51.2 ± 13.1  *Stage, n (%):* IA: 34 (40.0)/ IB: 5 (5.9)/ IIA: 7 (8.2)/ IIB: 2 (2.4)/ IIIA: 1 (1.2) /IIIC: 0  *Sx type, n (%): LUMP*: 15 (17.6); *LUMP + SNB:* 16 (18.8); *LUMP + ALND*: 0; *PMMX + SNB*: 21 (24.7); *PMMX + ALND*: 4 (4.7); *PMMX + Brecons*: 29 (34.1)  *Systemic treatment, n (%):*  Neoadj Ch: 18 (21.1)/Adj Ch: 27 (31.8)/ RT: 45 (52.9)/ IORT: 4 (4.7) | **Intervention group**  PT treatment that included therapeutic, stretching and strengthening exercises + patient education  Initiation: 2^nd^ pod  Duration: unclear  **Control group**  Usual nursing care (guidance on postoperative complications and instructions in case of persistence of symptoms more than 3w)  *Follow-up:* 1, 3 and 6m post-op | Pain  (NPRS)  UL function  (QuickDASH)  Shoulder ROM  (Goniometer application)  Presence of LE or AWS  (Patient self-reported) | *Disclosed for general population:*  NPRS  QuickDASH  Goniometer application | *Short-term postoperative complications (IG/CG)*  Hematoma: 1 (1.4)/ 5 (5.9); Bleeding: 4 (5.6)/ 3 (3.5); Revision Sx: 1 (1.4)/3 (3.5)  *Long-term postoperative complications (IG/CG)*  Seroma: 2 (2.8)/ 9 (10.6); Infection: 6 (8.3)/ 5 (5.9); LE: 9 (13.0)/ 6 (7.8); AWS: 8 (11.4)/ 11 (12.9)  There were no significant differences between groups |
| Lauridsen  (2005)  Denmark | RCT | N= 139  **Group A; n = 72**  *Age (age range):*  MRM + RT: 49 (40-70)/MRM: 60 (37-74)  BCS: 54 (31-79)  *Stage*: N/A  *Sx type, n (%):*  MRM + RT: 20(28)/ MRM: 21(29)/BCS: 31 (43)  *Systemic treatment, n (%):*  Ch: 26 (36)/ RT: 23(32)/ HT:25(35)  **Group B; n = 67**  *Age (age range):*  MRM + RT: 51 (29-70)/MRM: 63 (32-77)/BCS: 54 (32-69)  *Stage*: N/A  *Sx type, n (%):*  MRM + RT: 23 (34)/MRM: 13(19)/ BCS: 31(46)  *Systemic treatment, n (%):*  *Ch:* 21 (31)/ RT: 17(25)/HT: 17(25) | **Group A**  Team instructed PT program consisting of relaxation and strengthening exercises, combined to vein pump therapy and instruction in stretching of scar tissue  Initiation: 6^th^ to 8^th^ post-op w  Duration: 2 days/w for 6w  Exercise sessions duration: 60 min  **Group B**  ‘’Standard treatment of the ward’’ and were offered the same PT program after the 26^th^ post-op w  *Follow up:*  6, 12, 26 and 56w post-Sx | Shoulder function  (Constant Shoulder Score)  Presence of ‘’strings’’ in the axilla  (Physical assessment) | *Undisclosed* | 14 patients dropped out of the trial including  two patients who died and two patients who had  terminal disease, disabling them in attending the final follow-up. |
| Odynets  (2021)  Ukraine | RCT | N= 77  **Group A; n= 38**  *Age, mean ± SD*: 57.10 ± 1.37 *Stage, n (%):* I: 9 (24.0)/ II: 29 (76.0)  *Sx type, n (%):* Madden MX: 38 (100)  **Group B; n=39**  *Age, mean ± SD*: 57.40 ± 1.24 *Stage, n (%):* I: 10 (26.0)/ II: 29 (74.0)  *Sx type, n (%):* Madden MX: 39 (100) | **Group A**  Progressive muscular relaxation and visualization exercises + yoga intervention  Initiation: 2-3w after surgery  Duration: 1m  **Group B**  Yoga intervention only  Initiation: 2-3 w after surgery  Duration: 1m | Pain experience and intensity  (McGill Pain Questionnaire and VAS) | *Disclosed for cancer population:*  McGill Pain Questionnaire  *Undisclosed :*  VAS | A positive, friendly atmosphere during the training sessions and obtaining positive effects from the exercises were motivating factors for the patients to systematically attend the interventions throughout the month |
| Pace do Amaral  (2012)  Brazil | RCT | N= 131  **MT+UL exercises group; n = 65**  *Age, mean ± SD*: 55.0 ± 11.4  *Stage, n (%):*  I/II: 46 (72)/ III/IV: 18 (28)  *Sx type, n (%):* BCS: 15 (23)/ RM: 50 (77)  *Systemic treatment, n (%):*  Ch: 22(88)/ RT: 13(52)/ HT: 15(60)  **UL exercises group; n = 66**  *Age, mean ± SD:* 56.7 ± 11.7  *Stage, n (%):*  I/II: 38 (58)/ III/IV: 28 (42)  *Sx type, n (%):* BCS: 13 (20)/ RM: 53 (80)  *Systemic treatment, n (%):*  Ch: 27 (90)/ RT: 24(80)/HT: 18(60) | **Both groups**  Initiated physical therapy on the 1^st^ post op day according to the institutional routine  **MT+UL exercises group**  UL exercises sessions, followed by an MT protocol consisting of scapular and glenohumeral joint mobs and therapeutic massage applied by trained PT  Duration: 1 month  Frequency: twice a week  MT sessions duration: 20 min  **UL exercises group**  Outpatient physical therapy program combining UL exercises (19 movements of flexion, extension, abduction, add, internal and external rotation alone or combined) to precautions to prevent LE  Initiation: 3^rd^ post-op day  Duration: 1 month  Frequency: 3 times a week  Exercise sessions duration: 45 min  Sets/Reps: 1/10  *Follow-up*  1, 6, 12 and 18 months after Sx | Shoulder ROM  (goniometer)  UL function  (Modified-University of California at Los Angeles Shoulder Rating Scale)  Postoperative complications  (Observations made by the main investigator) | *Undisclosed*  Goniometer  Modified-University of California at Los Angeles Shoulder Rating Scale | One hundred thirty-six women were invited to enroll. Two of them were excluded because of a lesion of the  long thoracic nerve and three of them requested referral to another service (i.e., declined to participate). |
| Paskett  (2021)  USA | RCT | N= 568  **LEAP group; n=315**  *Age, year, median (range):* 58 (27-88)  *Grade, n (%):* Low: 65 (22.0)/Intermediate: 138 (46.6)/High: 93 (31.4)  *Sx type, n (%):* PMX or LUMP: 199 (64.8); MX: 108 (35.2)/ Missing: 5  *Type of node dissection, n (%):* SLND: 158 (50.6)/ ALND: 67 (21.5)/ SLND + ALND: 87 (27.9) *Systemic treatment, n (%):*  Ch: 109 (34.9)/ RT: 214 (68.6)  **EO group; n=253**  *Age, year, median (range):* 59 (24-83)  *Grade, n (%):* Low: 54 (22.5)/Intermediate: 93 (38.8)/High: 93 (38.8)  *Sx type, n (%):* PMX or LUMP: 155 (65.1); MX: 83 (34.9)/ Missing: 4  *Type of node dissection, n (%):* SLND: 100 (41.3)/ ALND: 64 (26.4)/ SLND + ALND: 78 (32.2) *Systemic treatment, n (%):*  Ch: 95 (39.3)/ RT: 166 (68.6) | **LEAP group**  LE education and prevention (LE etiology, signs, symptoms, treatments, preventive self-care practices) and exercise program (breathing, stretching, strengthening and ROM EX)  Initiation: ≤ 6w after Sx  Freq: daily  Exercises duration: 15 min  Duration: unclear  **EO group**  LE education and prevention only  *Follow-up:* 6, 12 and 18 months after Sx | Presence of LE  (arm CIRC)  Severity of LE  (change in arm CIRC at the site of greatest difference)  Shoulder ROM  (self-reported)  Adherence to study protocol | *Undisclosed*  Arm CIRC | Approximately half of the LEAP group performed the breathing, stretching and strengthening EX as instructed  ED group: 39(16.0) dropped out of the study; 8 pt refusal, 9 lost to follow-up, 9 Interviewer forgot, 6 disease progression/death  LEAP group: 68 (22.0); 33 pt refusal, 15 lost to follow-up, 1 Interviewer forgot, 11 disease progression/death |
| Petito  (2014)  Brazil | RCT | N=77  **Early group; n=40**  *Age, mean ± SD*: 55 ± 8  *Sx type, n (%):* MX: 24 (59)/QT: 17 (42)  **Late group; n=40**  *Age, mean ± SD*: 53 ± 12  *Sx type, n (%):* MX: 21 (57)/ QT: 16 (43) | **Exercise program (both groups)**  9 exercises outside hospital with illustrated manual  Duration: 105 post-operative days  Frequency: daily at home  **Early group**  Initiation: 1^st^ post op day  **Late group**  Initiation: After drain removal (postoperative day 7-10, mean postoperative day: 9)  *Follow up:*  Postoperative day 14, 45, 75 and 105 | Evaluation incision (presence of seroma formation and dehiscence)  ROM  (goniometer) | *Undisclosed* | n=14 did not attend 45^th^ postop day (8 in early group; 7 in late group)  n=2 return to Sx (in late group), n=1 died (in late group) |
| Rizzi  (2021)  Brazil | RCT | N= 60  **Free ROM group; n=30**  *Age (y), mean ± SD*: 55.06 ± 10.56  *Stage, n (%):*0: 6 (20.0)/ I: 23 (76.7)/ II: 1 (3.3)  *Sx type, n (%):* QT + symmetrization: 26 (86.7); Margin re-excision + symmetrization: 4 (13.3)  *Type of node dissection, n (%):*  SNB: 23 (76.7)/ALND: 1 (3.3)  *Systemic treatment, n (%):*  Neoadj Ch: 5 (16.7)  **Limited ROM group; n =30**  *Age (y), mean ± SD*: 52.53 ± 9.08  *Stage, n (%):*0: 3 (10.0)/ I: 23 (76.7)/ II: 4 (13.3)  *Sx type, n (%):* QT + symmetrization: 29 (96.7); Margin re-excision + symmetrization: 1 (3.3)  *Type of node dissection, n (%):*  SNB: 2 (66.6)/ALND: 5 (16.7)  *Systemic treatment, n (%):*  Neoadj Ch: 7 (23.3) | **Both groups**  Exercise protocol (UL ROM and cervical muscles stretching EX)  Initiation :1^st^ pod  Day1-14: Exercises 1-6  From Day15: Exercises 1-8  Duration: unclear  **Free ROM group**  Were allowed to perform the protocol exercises and ADL in free amplitude  **Limited ROM group**  Had ROM maintenance limited to 90° until the 30^th^ post-op day, then started free ROM exercises  *Follow-up:* PO 60 and 90 | Shoulder ROM  (Goniometer)  Pain intensity  (Analog verbal scale)  UL function  (DASH)  Presence of dehiscence, seroma, infection or necrosis  (inspection and/or palpation) | *Undisclosed:*  Goniometer  Analog verbal scale  DASH | During follow-up, there were 5 losses, of which 3 were from Free ROM group and 2 from the limited ROM group  Of the 30 patients with dehiscence,  17 (56.7%) presented with the continuous opening of the surgical wound (9 from the Free ROM group and 8 from the  Limited ROM Group), and 43.3% presented  opening points (4 from the Free ROM group and 9 from the  Limited ROM Group. 4 cases of  dehiscence (1 in the Free ROM group and 3 in Limited ROM  Group) and one of seroma (Limited ROM Group) occurred  between PO15 and PO30  33% of patients still had pain  3 m after surgery, with no ≠ between groups. |
| Sagen  (2009)  Norway | RCT | N= 207  **No activity restriction group (NAR); n=104**  *Age, mean ± SD*: 54 ± 90.6  *Sx type, n (%):* BSx: 46 (44)/ BCS: 57 (55)  *Systemic treatment, n (%):*  RT, nodes:47(45)/RT, breast:78(75)  Ch: 42 (40)/ HT: 48 (46)  **Activity restriction group; n=100**  *Age, mean ± SD*: 55 ± 90.6  *Sx type, n (%):* BSx: 51(51)/BCS: 49(49)  *Systemic treatment, n (%):*  RT, nodes:40(40)/RT, breast:73(73) Ch: 38 (38)/ HT: 50(50) | **No activity restriction group**  Supervised physical therapy program which emphasized moderate progressive resistance exercise training  Duration: 6 months  Frequency: 2-3 times a week  Exercise duration: 45 min  Rep: 15 / exercise  Load: 0.5kg the first 2 w increasing individually after  **Activity restriction group**  Physical therapy program with restricted activities of the OA avoiding heavy (>3kg) and strenuous activity.  *Program*: 6 different standardized passive manual techniques emphasizing flexibility and light massage of the affected shoulder, arm and scar.  Duration: 6 months  Frequency: 1/ week  Program duration: 45 min  *Follow up:*  3, 6 months and 2 yrs | Development of arm LE  (VOL diff in mL)  Pain and sensation of heaviness  (VAS) | *Disclosed for general population*  Water displacement  *Undisclosed*  VAS | Adverse events:  n=2 developed adhesive capsulitis with progressive immobilization and n=1 developed  supraspinatus tendinopathy  31/238 eligible participants who met the inclusion criteria refused to participate  The reasons for the 52 missing  individuals at follow-up were:  14 had died, three had moved elsewhere, 13 were not available, 7 refused to participate, four were too frail or ill, two had gone through ALDN on the control side, and nine were lost during follow-up. |
| Schultz (1997)  Sweden | RCT | N=163 with MRM  **Early postoperative shoulder exercise group; n=89**  Age, median (range): 59 (35-83)  **Delayed postoperative shoulder exercise group; n=74**  Age, median (range): 62 (41-84) | **Early postoperative shoulder exercise group**  Active shoulder exercise (anteflexion, abd, rotation)  Initiation: 1^st^ postop day  Frequency: 3 times/day  **Delayed postoperative shoulder exercise group**  Active shoulder exercise (anteflexion, abduction, rotation)  Initiation: 1^st^ postop w  Frequency: 3 times/day  *Follow up:*  1w, 4 and 6 months | Shoulder mobility (abd and anteflexion)  Volume of seroma aspirations and number of aspirations | *Undisclosed* | Postoperatively 31% (50/163) of the patients suffered  from seromas, 38% (34/89) in the early group and 22% (16/74) in the delayed group (Fig. 1). The difference was statistically significant. |
| Siedentopf (2013)  Germany | RCT | N=93  **Intervention group; n=48**  *Age, mean ± SD*: 55.82 ± 10.72  *Sx type, n (%):*  BCS: 29 (62)/RM: 18 (38)  SND: 37 (71)/ ALND: 15 (29)  *Systemic treatment, n (%):*  Ch: 17 (53)/ RT: 23 (70)  **Control group; n=41**  *Age, mean ± SD*: 58.41 ± 9.91  *Sx type, n (%):*  BCS: 24 (60)/ RM: 16 (40)  SND: 32 (78)/ ALND: 9 (22)  *Systemic treatment, n (%):*  Ch: 7 (30)/ RT: 16 (64) | **Intervention group**  Yoga classes  Initiation: Immediately after Sx  Duration: 5 w  Frequency: 2 times /w  Class duration: 75 minutes  10 classes over 5 w  **Control group**  *Yoga classes*  Initiation: 5 weeks after Sx  Duration: 5 w  Frequency: 2 times / w, 10 classes  Class duration: 75 minutes  Yoga class: started with lying postures and the gradual mobilization of arms and legs + breathing exercises + dynamic part of the exercises: standing and sitting positions + Eye exercises + series of concentration exercises. | Quality of life  (German version of the European Organization of Research and Treatment of Cancer Quality of Life questionnaire (EORTC QLQ-C30) and its breast-cancer-specific module EORTC QLQ-BR23) | *Cancer-specific outcome(s)*  EORTC QLQ-C30  *BC-specific outcome(s)*  EORTC QLQ-BR23 | Due to the high number of participants who dropped out for various  reasons (16 in the IG, 16 in the WG) only 31 women  completed the intervention in the IG and 28 women in the WG. |
| Temur  (2019)  Turkey | RCT | N= 72  **Intervention group; n = 36**  *Age, mean ± SD*: 46.7 ± 9.96  *Stage, n (%):*  I: 2 (7)/ II: 16 (53)/ III: 12 (40)  *Sx type, n (%):*  MRM: 22 (73)/ BCS: 8 (27)  **Control group; n= 36**  *Age, mean ± SD*: 45.6 ± 9.03  *Stage, n (%):*  I: 2 (7)/ II: 16 (52)/ III: 13 (12)  *Sx type, n (%):*  MRM: 17 (55)/ BCS: 14 (45) | **Intervention group**  Self-management of LE program (SMLP) + exercising program + simple LD+ closed monitoring to encourage the patient to do the exercises and to follow the instructions provided  *SMLP program:* Training booklet containing information about mechanisms and risk factors of LE and about prevention interventions.  *Exercising program:*  1^st^ 24h: Hand squeezing exercises with a medium-level stress ball  Frequency: 4 times/day  Reps: 15  Active and passive arm exercises, the minimum amount of exercises required for the patient were determined by the doctor and nurse  Initiation: within the first 24h post-Sx  Duration: 6 months  Frequency: 3-6 times/day at first and gradually increased to 10  Exercise sessions duration: 30-60 min  *Simple lymphatic drainage*  The researcher demonstrated and taught deep diaphragmatic breathing exercises, neck drainage, unaffected and affected side axillary drainage and UE drainage.  Frequency of breathing exercises: 3 times a day  Breathing exercises duration: 3 min initially, gradually progress to 5 min  Frequency of self-massage: 2 times a day  Reps: 8-10 per session  **Control group**  No intervention during the study period, except usual post op care. Training booklets were distributed at the end of the study and patients who volunteered for ED were trained about SMLP  *Follow-up:*  1, 3 and 6 months post-intervention | Upper extremity function  (DASH)  Presence of LE- upper extremity CIRC  (Measuring tape)  Quality of life  (EORTC QLQ-30 and EORTC QLQ-BR23) | *BC-specific outcome*  Upper extremity CIRC measurement  EORTC QLQ-BR23  *Cancer-specific outcome*  EORTC QLQ-30  *Disclosed for general population*  DASH | *Lost to follow-up:*  IG: did not follow the research program (n=2), wound site revision in the post-operative period during the research (n=3), developed skin reaction after RT (n=1)  CG: did not follow the research program (n=1), wound site revision in the post-operative period during the research (n=2), developed skin reaction after RT (n=2)  Feedback was received from the patients during the interviews,  and it was found that telephone calls relieve and motivate the patients.  The inclusion of the researcher's photograph in the booklet increased  their motivation and adaptation to the program |
| Teodózio  (2020)  Brazil | RCT | N=572  **Free ROM group, n=254**  *Age, mean ± SD*: 52.54 ± 12.03  *Sx type, n (%):*  Segmentectomy: 107 (42)  MX: 147 (58)  **Restricted ROM group, n=211**  *Age, mean ± SD*: 54.53 ± 10.95  *Sx type, n (%):*  Segmentectomy: 94 (45)  MX: 117 (56) | **Free ROM group**  Active UL movements with ROM over 90° (flexion and abd of shoulder) (leaflet + home guide)  Initiation: 1^st^ postop day  Frequency: 3 times/day (at least once a day)  **Restricted ROM group**  Active UL movements with ROM restricted to 90° (flexion and abd of shoulder) from postoperative day 1 until removal of all surgical stitches (leaflet + home guide)  Frequency: 3 times / day (at least once a day)  *Follow up:*  Postoperative day 1 and 30 days | Presence of seroma  Necrosis  Dehiscence  Hematoma  Infection  Bruise | *Undisclosed* | n=73 refused to participate  n=34 Sx not performed on schedule date  Drop out during follow up: non-return to the wound-dressing  clinic (*n* = 1), hospitalization for reasons other than a surgical approach (*n* = 2) and changes to the treatment protocol (*n* = 1) |
| Testa  (2014)  Italy | RCT | N=70  **Treated group, n=35**  *Age, mean ± SD*: 54.3 ± 8.02  *Stage*: N/A  *Sx type, n (%):*  Maddens’ MRM: 19 (54)  Segmental MX + ALDN: 16 (45)  *Systemic treatment, n (%):*  Ch: 24 (69)/ RT: 30 (86)  **Control group, n=35**  *Age, mean ± SD*: 55.3 ± 8.5  *Stage*: N/A  *Sx type, n (%):*  Maddens’ MRM: 21 (60)  Segmental MX + ALDN: 14 (40)  *Systemic treatment, n (%):*  Ch: 25 (71)/ RT: 27 (77) | **Treated group**  Early physical rehabilitation program with instructions of a PT from latest guidelines for rehabilitation in BC published in 2005 by Italian society of senology  Initiation: 2^nd^ postop day  Program duration: 40 min  Frequency: 5 times / w during all the duration of axillary drainage  *Exercises:* active flexion, extension movement of the cervical spine, lateral flexion and rotation + passive and active assisted caution mobs of hand, wrist, elbow (flexion, extension, pronation, supination)  3^rd^ postop day: Passives exercises of flexion, abd, add and circumduction (25 min) + active internal, external rotation arms’ movements (15 min)  Once drainage removed (approximatively postoperative day 7): 20 PT sessions  Frequency: 5 times / w  Duration: 60 min / session  Exercises: postural and stretching exercises (20 min) + active movements of abd flexion, internal and external rotation of glenohumeral joint (40 min)  **Control group:**  *N*o early physical rehabilitation program with no instructions of a PT  Rehabilitation program from the old rehabilitation guidelines  *Follow up:* Postoperative day 5 and 1, 6 and 12 months | Mobility of the glenohumeral joint  (goniometer)  Grade of pain perceived  (VAS)  Quality of life  (EORTC QLQ30 and QLQ-BR23) | *Undisclosed* | - |
| Todd  (2008)  UK | RCT | N= 116  **Delayed shoulder mobs; n=58**  *Age, mean ± SD*: 56.5 ± 12.4  *Stage, n (%):*  I: 8 (14)/ II: 24 (41)/ III: 26 (45)  *Sx type, n (%):*  WLE: 36 (57)/ MX: 24 (43)  *Systemic treatment, n (%):*  RT: 39 (67)/Ch: 30(52)/HT: 34 (59)  **Early full shoulder mobs; n= 58**  *Age, mean ± SD:* 57 ± 14  *Stage, n (%):*  I: 8 (14)/ II: 27 (48)/III: 23 (38)  *Sx type, n (%):*  WLE: 29 (50)/ MX: 29 (50)  *Systemic treatment, n (%):*  RT: 41 (71)/Ch: 26(45)/ HT: 41(71) | **Delayed shoulder mobilization**  Exercises program that limited arm movements < 90° in all planes, followed by a full shoulder ROM program  Initiation:  *Limited ROM program*: 2^nd^ postop day  *Full ROM program:* 2^nd^ post op w  **Early full shoulder mobilization**  Full shoulder mobs (i.e., movement > 90°) and shoulder ROM exercises  Initiation: 2^nd^ postop day  **Both groups**  Exercise sessions duration: 10 minutes  Frequency: 4 times/day until full shoulder ROM was restored and then once/day for the 1^st^ postop year  Repetitions: 3-4 per exercise  The exercise programme was supervised  during inpatient stay (average 7 days).  *Follow-up:*  1 week, 1 month and 6 months | Incidence of LE-limb VOL difference  (Water displacement)  Shoulder ROM  (Manual goniometer)  Grip strength  (hand-held dynamometer)  Health-related QoL  (FACT-B+4 and SDQ) | *BC-specific outcome*  FACT-B+4  *Disclosed for general population*  Manual goniometer  SDQ  Hand-held dynamometer  Water displacement | 116 (36% of eligible patients) women agreed to take part in the study and seven (6%) withdrew  during the 1^st^ year  Reasons for  declining to participate: lack of interest or unwillingness  because of anxiety about impending S_x_  Most women (73%, n = 85) claimed that they had adhered to their exercise programme |
| Torres  (2010)  Spain | RCT | N= 120  **Early physiotherapy group;n =60**  *Age, mean ± SD*: 52.9 ± 10.7  *Stage*: N/A  *Sx type, n (%):* QT: 24 (40)/Modified MX: 23 (38)/ LUMP:13 (22)  *Systemic treatment, n (%):*  RT: 44 (75)/Ch: 50(85)/HT: 39 (66)  **ED strategy group; n = 60**  Age, mean ± SD: 52.9 ± 12.5  *Stage*: N/A  *Sx type, n (%):* QT: 26 (43)/ Modified MX: 20 (34)/LUMP:14(23)  *Systemic treatment, n (%):*  *RT*: 49 (86)/*Ch*: 45(79)/*HT:* 33 (58) | **Early physiotherapy group**  MLD + progressive massage of the scar, stretching exercises and progressive active and action assisted shoulder exercises, combined with functional activities and proprioceptive neuromuscular exercises + educational strategy  Initiation: 3 to 5 days after hospital discharge  **Educational strategy only group**  Instruction with printed materials about the lymphatic system, concepts of normal load vs overload, source of 2ndary LE, precipitating factors and 4 preventive interventions  Initiation: 3 to 5 days after hospital discharge  Duration of both programs: 3 w  Frequency of both programs: 3 times/ w  *Follow-up:*  1w, 3 months, 6 months and 12 months post-Sx | Incidence of secondary LE  (Arm CIRC) | *BC-specific outcome(s)*  Arm CIRC | Overall, 116 women completed the follow-  up assessments; 59 in the intervention group and 57 in the control group. |
| Wingate  (1989)  USA | RCT | N= 115  **Treated group, n=61**  *Age, mean*: 56.26  **Control group, n=54**  *Age, mean:* 58.27 | **Treated group**  Physical therapy  Initiation: 1^st^ postop day 1  Duration: 8 w minimum  Frequency: 2 session / day  Exercise sessions duration: 30 min  Exercises: motor and sensory status, active hand, wrist, elbow and postural exercises, active and active assisted shoulder exercises, functional activities and PNF  After drain removal: home exercises program with progressive restrictive exercises and PNF  **Control group**  Untreated group with no physical therapy  *Follow up:*  Postoperative day 5 and 1-3 months | Psychopathologic self-report inventory  (SCL-90-R)  Shoulder ROM for flexion and abd  (goniometer)  Functional evaluation of the ipsilateral shoulder  (Scale of difficulty)  Upper extremity CIRC measurement | *Cancer-specific outcome:*  SCL-90-R  *Undisclosed:*  Goniometer  Scale of difficulty  5 levels of upper extremity CIRC measurement | - |
| Zhang  (2016)  China | RCT | N= 1000  **Physical exercise group; n=500**  *Age group, n (%):*  *<50:*  272 (54)/ *≥50:* 228 (46)  *Stage, n (%):*  I/II: 211 (42)/ III: 289 (58)  *Sx type, n (%):* MRM: 500 (100)  **MLD group; n=500**  *Age group, n (%):*  <50: 266 (53)/ ≥50: 234 (47)  *Stage, n (%):*  I/II: 197 (39)/ III: 303 (61)  *Sx type, n (%):* MRM: 500 (100) | **Physical exercise group**  Physical exercise alone  Initiation: 24h before Sx with ED (risk of postsurgical complications and importance of medical intervention and self-management)  Frequency: post-operative day 1, 2, 3 and day of discharge  Session duration: 20-30 min  Postop day1-7 (before drain removal:  Physical exercise program  Passive exercises  Frequency: 3 times / day  Session duration: 15 min.  Postop day7-30: (after drain removal to sutures removal):  Exercises progressed to localized exercises on the affected UL  After removal sutures to 6 months:  Extensive active exercises involving affected shoulder  Frequency: 3 times / day  Session duration: 15 min.  **MLD group**  Physical exercises + Self-MLD  Initiation manual drainage: after sutures removal  Frequency: 3 sessions / day  Session duration: 30 min.  3 steps of 10 min.  *Follow up:*  Week 1, 1, 3, 6 and 12 months | Stage of UL LE  (Observation and tape-measuring)  Scar formation  (Vancouver Scare Scale)  Shoulder function  (max. shoulder abd) | *Undisclosed* | By the third month after Sx, only 4 patients in the MLD group had developed scar contracture, while 12 had developed scar contracture in the PE group. |
| Zhou  (2019)  China | RCT | N=92  **Intervention group; n=46**  *Age, mean ± SD*: 49.94 ± 8.88  *Stage, n (%):*  I: 18 (35)/ II: 27 (53)/ III: 6 (12)  *Sx type, n (%):*  MX + SND: 24 (47)/MX + ALND: 15 (29)  BCS + SND:10(20)/BCS +ALND:2(4)  *Systemic treatment, n (%):* Ch: 41 (80)  **Control group; n=46**  *Age, mean ± SD*: 49.40 ± 9.88  *Stage, n (%):*  I:14(28)/II: 29(57)/III: 8(16)  *Sx type, n (%):*  MX +SND: 25(49)/MX + ALND: 17 (33)  BCS + SND: 6(12)/BCS +ALND: 3(6)  *Systemic treatment, n (%):*  Ch:43 (84) | **Intervention group**  Progressive UL exercises and muscle relaxation training by nurses  Initiation: before Sx  Duration: 6 months  Frequency: 1 session/ day at hospital and 1 session/ week at home after discharge  **Control group**  Routine nursing care (Sx district nursing, drainage tube nursing, routine health ED, physical exercises, vital sign monitoring and post-Sx complications)  *Follow up:*  1, 3, 6 months | Quality of function  (Constant-Murley Score)  Health related quality of life  (FACT-Bv4.0) | *Undisclosed* | All intervention group patients completed the exercises  and training, with no adverse events and a compliance of  100%. |
| Zimmermann  (2012)  Germany | RCT | N=67  **MLD group; n=33**  *Age, mean ± SD*: 60.3 ± 8.2  Stage, n (%):  I: 12 (36)/ II: 15 (46)/ III: 6 (18)  *Sx type, n (%):*  BCS: 20 (61)/ MRM: 13 (39)  SND:14 (42)/ ALND: 19 (58)  *Systemic treatment, n (%):*  Ch: 13 (39)/RT: 22 (67)  **Control group; n=34**  *Age, mean ± SD*: 58.6 ± 12.2  *Stage, n (%):*  I: 11 (32)/II: 16 (47)/III: 7 (21)  *Sx type, n (%):*  BCS: 20 (59)/ MRM: 14 (41)  SND: 18 (53)/ ALND: 16 (47)  *Systemic treatment, n (%):*  Ch: 15 (44)/RT: 25 (74) | **Both groups**  Exercises of limb and chest physiotherapy  Initiation: 2^nd^ postop day  **MLD group**  *Manual lymph drainage*  Initiation: 14^th^ postop day  Duration: 6 months  Frequency: 5 sessions/ week  **Control group**  Applied self-drainage from modification of the method described by Földi and Strönbenreuher  *Follow up:*  6 months | VOL of both arms (water displacement  With glass cylinder with water)  VOL of LE | *Undisclosed* | In the present study, all women who had received MLD on day 2 after Sx and continued receiving it for the 6 ensuing months did not develop secondary LE of the arm on the operated side. In the group of women without MLD, 6 months after Sx, 70.6% of the subjects suffered from LE**.** |
| Majed  (2020)  USA | RCT | N= 69  BC women undergoing MRM  **Intervention group;** **n=35**  *Age group, n (%):*  35-42: 14 (47)/43-48: 10 (33)  49-55: 6 (20)  **Control group; n=34**  Age group, n (%):  35-42: 14 (47)/43-48: 10 (33)  49-55: 6 (20) | **Intervention group**  *Phase 1*  Initiation: day prior to Sx  Measurements: QoL-BC survey and shoulder ROM  *Phase 2*  Initiation: Prior to Sx  Intervention: one-to-one ED (PowerPoint presentation regarding the therapeutic exercises, information about the surgery, and a booklet with pictures of the exercises to take home) in addition to routine hospital care. Demonstration of the exercises by the researcher with a return demonstration by the patient was done.  Follow up every week by phone.  *Phase 3*  Exercise program  Initiation: Immediately after Sx  Exercises: deep breathing + shoulder exercises (extension of the triceps, bicep curl, paddling in sitting position, fluttering with both arms, hands behind neck, forward wall crawls, and side wall crawls). Shoulder flexion was limited to 90° of assisted AROM for the first few days post-Sx and until the drains were removed, gradually increased after the 3^rd^ postoperative day.  Rep: 10 of each exercise during hospitalization  **Control group**  *Phase 1*  Initiation: day prior to Sx  Measurements: QoL-BC survey and shoulder ROM  *Phase 2*  Initiation: Prior to Sx  Intervention: routine hospital care (explanation by the surgeon on the surgical procedure).  *Phase 3*  Initiation: At home  Frequency measurements of ROM and questionnaire: w2 and w4 after Sx | Quality of life  (Breast Cancer Patient  Version (QoL-BC))  Shoulder ROM (Goniometer) | *BC specific outcomes*  Breast Cancer Patient Version (QoL-BC)  Goniometer | 7 were excluded because they didn’t fit criteria or declined to participate, 5 refused to do the exercises, 4 lost to follow up |
| de Almeida Rizzi  (2020)  Brazil | RCT | N= 62  **Free ROM group; n = 31**  *Age, mean ± SD*: 49.90 ± 10.11  *Stage, n (%):*0: 10 (33)/ I: 4 (13)/ II: 3 (10)  IIB:7(23)/III:5(17)/IIIB:1(3)/IV:0(0)  *Sx type, n (%):*  Breast sparing Sx: 14 (47)  MX: 16 (53)/Breast recons: 30(97)  *Type of node dissection, n (%):*  SNB: 15 (50)/ALND: 14 (47)  *Systemic treatment, n (%):*  Neoadj Ch: 13 (43)  **Limited ROM group; n = 31**  *Age, mean ± SD*: 54.46 ± 10.68  *Stage, n (%):* 0: 11 (37)/ I: 4 (13)/ II: 7 (23)/ IIB: 5 (17)/ III: 2 (7)/ IIIB: 0 (0)/IV: 1 (3)  *Sx type, n (%):*  Breast sparing Sx: 10 (33)  MX: 20 (67)/Breast recons: 30(97)  *Type of node dissection, n (%):*  SNB: 21 (70)/ ALND: 7 (23)  *Systemic treatment, n (%):*  Neoadj Ch: 10 (33) | **Both groups**  Exercise protocol consisting of neck and UL stretching exercises and shoulder ROM exercises  Initiation: First post-op day  Day1-14: Exercises 1-6  From Day15: Exercises 1-8  **Free ROM group**  Were allowed to perform the protocol exercises and ADL in free amplitude  **Limited ROM group**  Had ROM maintenance limited to 90° until the 30^th^ post-op day, then started free ROM exercises  *Follow up*  Day 7, 15, 30, 60 and 90 postoperatively | Dehiscence  (Inspection, palpation and tape measure)  Seroma  (Inspection and palpation, medical record)  Infection  (Inspection and palpation, medical record)  Necrosis  (Inspection and medical record)  Shoulder ROM  (Goniometer)  Pain  (VAS)  UL function (DASH) | *Undisclosed* | Limited ROM group: Discontinued:  reoperation before PO30:  removal of extruded  prosthesis  The free ROM group presented one case of  dehiscence that started after PO30 (late), and the limited ROM group presented two cases between PO15 and PO30  (intermediate) and one case after PO30 (late).  Pain complaint was present in all the PT  evaluations of both groups, from moderate to severe intensity |
| de Oliveira  (2014)  Brazil | Controlled non-randomized clinical trial | N= 96  **Exercise group; n = 48**  *Age, mean ± SD*: 56.7 ± 15.1  *Stage, n (%):*  I: 1 (2)/ II: 17 (37)/ III/IV: 28 (61)  *Sx type, n (%):* *MRM:* 48 (100)  *Systemic treatment, n (%):*  *Neoadj Ch:* 22 (48)  **MLD group; n = 48**  *Age, mean ± SD*: 55.6 ± 11.9  *Stage, n (%):*  I: 0 (0)/ II: 9 (20)/III/IV: 34 (79)  *Sx type, n (%):*  MRM: 42 (62)/Halsted RM: 1 (2)  *Systemic treatment, n (%):*  Neoadj Ch: 29 (67) | **Both groups**  *Educational strategy:* Information leaflets about proper care for the OA and lectures  Initiation: 1^st^ post-op day  **Exercise group**  19-exercise supervised program including neck and rotator cuff muscles stretching and active assisted and free AROM exercises  Initiation: 3^rd^ post-op day  Duration: 2 days/w for 30 days  Exercise sessions duration: 40 min  **MLD group**  Manual lymphatic drainage applied by 3 experienced PT  Initiation: 3^rd^ post-op day  Duration: 2 days/w for 30 days  MLD sessions duration: 40 min | UL CIRC  (Measuring tape)  Shoulder ROM  (Goniometer)  Scarring complications  (Signs of wound dehiscence, infection, seroma and puncture) | *Disclosed for general population:*  Goniometer  *Undisclosed:*  Measuring tape | There was no difference in shoulder ROM, wound healing complications or arm circumference, between women who received MLD or performed active EX |
| Huo  (2021)  China | Controlled non-randomized clinical trial | N= 93  **Observation group; n=47**  *Age, mean ± SD*: 48.5 ± 7.0  *Stage, n (%):* I: 7 (14.9)/ II: 22 (46.8)/ III: 18 (38.3)  *Sx type, n (%):* MRM: 47 (100)  **Control group; n= 46**  *Age, mean ± SD*: 47.8 ± 6.4  *Stage, n (%):* I: 5 (10.9)/ II: 27 (58.7)/ III: 14 (30.4)  *Sx type, n (%):* MRM: 47 (100) | **Observation group**  Routine nursing care + personalized rehabilitation EX intervention  Initiation: 24h post-Sx  Duration: up to 6m post-Sx  **Control group**  Routine nursing care | Immune function  (Blood sample)  UL edema  (arm CIRC)  Presence of subcutaneous fluid  (Teiler’s approach)  Shoulder ROM  (Goniometer)  UL function  (DASH questionnaire, ADL score)  QoL  (FACT-B) | *Disclosed for the population of interest:*  FACT-B  *Undisclosed:*  Arm CIRC  Teiler’s approach  Goniometer  DASH  ADL score | The incidences of UL edema and subcutaneous fluid were lower in the observation group (2.1%) than the control group (19.6%; 17.4%) |
| Na  (1999)  South Korea | Non-randomized clinical trial | N= 33  **Rehabilitation group; n = 20**  *Age, mean ± SD*: 43.8 ± 2.1  *Stage*: N/A  *Sx type, n (%):*  MRM: 15 (75)/Partial MX: 5 (25)  **Control group; n = 13**  Age, mean ± SD: 46.9 ± 9.8  Stage: N/A  *Sx type*, n (%):  MRM: 7 (54)/Partial MX: 6 (46) | **Rehabilitation group**  Early postmastectomy rehabilitation program  Initiation: 1^st^ postop day  Duration: Patients received instructions to pursue the program at home for 4w  Program duration: 40 minutes of PT and 30 minutes of exercises  Frequency: 4 times a day  *1^st^ postop day:* Postural exercises, AROM of the shoulder, elbow, wrist, and hands with active use of the involved arm  *From the 3^rd^ postop day:* Physical modalities for pain relief (PENS, heat therapy, cold therapy) and therapeutic exercises including ROM exercises.  Patients with LE received IPC and those with myofascial pain or muscle spasm received trigger points injection with lidocaine  *After drains removal:* Progressive resistance exercises of the UE with an increase in functional activities  **Control group**  No rehabilitative treatment, instruction alone for ROM exercises pertaining to the affected shoulder and postural exercises  *Follow up:* 3^rd^ post op day, at discharge and 1 month after discharge | Symptoms Checklist  (SCL-90-R)  Shoulder ROM  (Goniometer)  Shoulder function  (10 items provided by Wingate)  UL CIRC  (Tape measurement) | *Cancer-specific outcome(s):*  SCL-90-R  *Disclosed for general population*:  Goniometer  *Undisclosed:*  Items provided by Wingate  Circumference measurement | Postoperative complications or problems  Control group; wound breakdown (7.7%), adhesional bands (15.4%), sensory changes around surgical incisions (38.5%)  Rehabilitation group; mild edema (5%), wound breakdown (5%), adhesional bands (15%), sensory changes (9%) |
| Oliveira  (2018)  Brazil | Non-randomized clinical trial | N=116  **Active exercise group; n=58**  *Age group, n (%): <55:*  22 (42)/ *≥55:* 31 (59)  *Stage, n (%):*  I: 1 (20)/II: 17 (34)/III/IV: 32 (64)  *Sx type, n (%):*  MRM Patey: 29 (55)/MRM Madden: 24 (45)  RM Halsted: 0 (0)  *Systemic treatment, n (%):*  Neoadj Ch: 24 (45)/Adj Ch: 8 (36)  RT: 16 (73)/ HT: 14 (64)/ IT: 3(14)  **MLD group; n=58**  *Age group, n (%): <55:*  24 (45)/*≥55:* 29 (55)  *Stage, n (%):*  I: 0 (0)/II: 9 (18)/III/ IV: 43 (82)  *Sx type, n (%):*  MRM Patey: 19 (36)/MRM Madden: 33 (62)  RM Halsted: 1 (2)  *Systemic treatment, n (%):*  Neoadj Ch: 36 (68)/Adj Ch: 18 (62)  RT: 26 (87)/HT:18 (60)/ IT: 5 (17) | **Both groups**  Educational strategy: Information leaflets about proper care for the OA and daily active exercises to do at home) + lectures delivered by the multidisciplinary team during the first month after Sx*.*  Initiation: 1^st^ postop day  **Active exercise group**  Initiation: 48h after Sx  Duration: 30 days  Frequency: 40 min group session, 2/w  Exercises: 30% of the time – stretching of the scalene muscles, trapezius muscles, levator scapulae muscles, pectoralis major and minor muscles, rotator cuff muscles of the shoulder; 60% of the time – active assisted exercise and free active exercise for shoulder flexion, abd, add, internal and external rotation of the UL alone or combined, followed by stretching of the deltoid, latissimus dorsi, rhomboids and pectoralis muscles; and 10% of the time-relaxation, according to treatment protocol of the service  **MLD group**  Manual lymphatic drainage  Initiation: 48h after Sx  Duration: 30 days  Frequency: 40 min individual session, 2/w  *Follow up:* one week before Sx, 2 and 30 months | Velocity visualization of axillary lymph nodes and degree uptake in axillary lymph nodes  (Lymphoscintigraphy)  ROM  UL CIRC | *Undisclosed* | Active exercise group: 3 missed 2 consecutive sessions + 2 lost follow up at 2 months and 17 death and 13 lost to follow up at 30 months  MLD group: 1 missed 2 consecutive sessions + 4 lost follow up at 2 months and 19 death and 4 lost to follow up at 30 months |
| Tirolli Rett  (2022)  Brazil | Controlled non randomized clinical trial | N= 65  *Age, mean ± SD*: 50.61 ± 11.14  *Sx type, n (%):* MRM: 40 (81.6); QT: 9 (18.4)  *Systemic treatment, n (%):*  Ch: 29 (59.1)/ RT: 23 (46.9) | **PT protocol**  Initiation: Between 4-8w after Sx  Freq: 3 times/w  Sets/Reps: 3/8-12  Consultation duration: 60 min  Duration : 20 sessions, 7w | Shoulder ROM  (Goniometer)  Pain intensity and experience  (VAS and McGill Pain Questionnaire) | *Undisclosed*  Goniometer  VAS | Of the 65 women selected, 49 completed the study (dropped out n=12; death n=2; did not answer the questionnaires n=2 |
| Kim  (2019)  South Korea | Retrospective case-control study | N= 115  **Early rehabilitation group; n = 49**  *Age (age range):* 43 (34-61)  *Stage*: N/A  *Sx type*: Skin-sparing total MX and immediate Brecons with tissue expander  *Type of node dissection, n (%):*  SNB: 41 (84)/ ALND: 8 (16)  *Systemic treatment, n (%):*  Neoadj Ch: 3 (6)  **Conventional protocol; n = 66**  *Age (age range):* 42 (24-61)  *Stage*: N/A  *Sx type*: Skin-sparing total MX and immediate Brecons with tissue expander  *Type of node dissection, n (%):*  *SNB:* 46 (70)/ *ALND:* 20 (30)  *Systemic treatment, n (%):* Neoadj Ch: 7 (11) | **Both groups**  Self-exercise ED  Initiation: 1^st^ post-op w  **Early rehabilitation group**  Short term immobilization period (2w) followed by a self-exercise program including progressive shoulder stretch exercises and strengthening exercises  Initiation: 3^rd^ post-op w  Frequency: 4 times a day/ 7 days per w  Sets/Repetitions: 1 / 5-10  **Conventional protocol**  Were asked to immobilize the OA for more than 4w and engaged themselves in the same self-exercise program after the immobilization period  Initiation: From the 5^th^ post-op w  Frequency: 4 times a day/ 7 days per w  Sets/Repetitions: 1 / 5-10  *Follow up:*  1 and 2 months postoperatively | Shoulder ROM  (goniometer)  Pain  (NRS-11)  QoL  (SF-36)  UL function  (DASH)  Postoperative complications  (Plastic surgeon assessment) | *Disclosed for BC survivors:*  SF-36  *Disclosed for patients with adhesive capsulitis:*  Goniometer  *Disclosed for general population:*  NRS-11  DASH | No surgical sites complications that can be attributed to the intervention  At the 1-month follow-up, 4 patients in the conventional group were diagnosed with secondary adhesive capsulitis |
| Lu  (2015)  Taiwan | Retrospective cohort study | N= 1217  **Group A; n= 415**  *Age, mean ± SD*: 51.79 ± 11.97  *Stage, n (%):*0-2: 326 (79)/ 3: 89 (21)  *Sx type, n (%):*  BCS: 123 (30)/Simple MX: 25 (6)  MRM: 267 (64)  *Systemic treatment, n (%):*  RT: 182 (44)/ Ch: 342 (82)  **Group B; n = 672**  *Age, mean ± SD*: 52.67 ± 11.01  *Stage, n (%):* 0-2: 503(75)/ 3:169 (25)  *Sx type, n (%):* BCS: 152(23)/  Simple MX:11(2)/ MRM: 509(76)  *Systemic treatment, n (%):*  RT: 297 (44)/ Ch: 549 (82)  **Group C; n = 130**  *Age, mean ± SD*: 51.88 ± 10.08  *Stage, n (%):* 0-2: 92 (71)/ 3: 38 (29)  *Sx type, n (%):* BCS: 303(25)  Simple MX: 41(3)/MRM: 873(72)  *Systemic treatment, n (%):*  RT: 66 (51)/ Ch: 111 (85) | **Group A**  No ED or PT provided  **Group B**  ED only which provided information on the lymphatic system, the symptoms and signs of LE, suggestions for preventing LE.  **Group C**  ED + PT sessions which included the following treatments: breathing exercise, postsurgical positioning, massaging of scar tissue, mobs of the shoulders and UE exercises, passive and active stretching of the major and minor pectoral muscles  Initiation: 1^st^ postop w in the hospital and was continued at outpatient clinics post discharge  Frequency: 2 times/w  PT sessions duration: 30 min | Occurrence of LE  (Limb-to-limb CIRC difference)  LE severity  (Criteria defined by the International Society of Lymphology) | *Disclosed for patients with LE*  Criteria defined by the International Society of Lymphology  *Undisclosed*  Limb CIRC measurement | - |
| Manfuku  (2021)  Japan | Retrospective case-control study | N= 153  **BME + PT group; n =78**  *Age, mean ± SD*: 54.2 ± 9.8  *Stage, n (%):* 0-I: 28 (48.3)/ II-III: 30 (51.7)  *Sx type, n (%):* MX: 28 (48.3); BCS: 30 (51.7)  *Type of node dissection, n (%):* SNB: 39 (67.3)/ ALND: 19 (32.8) *Systemic treatment, n (%):*  Ch: 23 (39.7)/ RT: 37 (63.8)/ HT: 42 (72.4)  **PNE + PT group; n= 75**  *Age, mean ± SD*: 52.3 ± 11.3< *Stage, n (%):* 0-I: 35 (58.3)/ II-III: 25 (41.7)  *Sx type, n (%):* MX: 37 (61.7); BCS: 23 (38.3)  *Type of node dissection, n (%):* SNB: 42 (70.0)/ ALND: 18 (30.0) *Systemic treatment, n (%):*  Ch: 17 (28.8)/ RT: 32 (53.3)/ HT: 43 (71.7) | **BME + PT group**  PT program that comprised shoulder joint EX and mobs + educational sessions on breast anatomy and surgical procedures  Initiation: 1w before Sx  Duration: 3m  **PNE + PT group**  PT program + educational sessions on pain mechanisms (purpose was to change the patient’s knowledge of their pain states)  Initiation: 1w before Sx  Duration: 3m  *Follow-up*: 1 year after Sx | Pain intensity and pain interference  (BPI)  Shoulder ROM  (Goniometer)  Handgrip strength  (Dynamometer)  CS-related symptoms  (CSI)  Pain-related catastrophizing  (PCS)  Presence of LE  (arm CIRC) | *Disclosed for cancer population*  BPI  *Disclosed for general population*  CSI  *Undisclosed*  Goniometer  Dynanometer  Arm CIRC | *Dropouts:* BME group: 6 (7.7); PNE group: 4 (5.3); no reasons were provided  At 1-year follow-up, 14 participants from the BME group and 11 from the PNE group were excluded from analysis as their main pain was cancer treatment-related pain and not PPMP |
| Morimoto  (2003)  Japan | Prospective observational study | N=72  BC women stage I or II  **PCM group; n=33**  *Age, mean ± SD*: 50.0 ± 11.0  *Stage*: N/A  *Sx type*: PCM  **BCS group; n=38**  *Age, mean ± SD*: 50.8 ± 8.8  *Stage*: N/A  *Sx type*: BCS | **Both groups**  Initiation: postoperative day 1  Duration: After hospital discharge (postop day 10-14) was entrusted to the patient’s own initiative  *Postoperative day 1:*  Prevention of development of rigidity of shoulder joint on the OA: Lateral and forward arm raising on the affected side in the dorsal sitting positions  *Postoperative day 2:*  Training for force releasing through exercise of shoulder joint: Backward arm raising, finger crawling on a wall  and force releasing/concentrating by keeping the arm on affected side at a position of backward raising at 90°  *Postoperative day 3:*  Exercise to approximate preoperative life: Exercises such as wing flapping and touching the ears and back with the hands  *Postoperative day 4:*  Exercise to reduce functional differences between the normal and affected sides: Reduction of right and left side differences through the above-mentioned exercises on postoperative days 1–3  *Follow up:*  Before Sx, postoperative w1, 2, 4, 12 | Shoulder joint ROM  (goniometer)  Grip strength  (Instrument not provided)  Pain after Sx  (Instrument not provided)  Movement associated chest pain  (Instrument not provided)  Operative wound pain  (Instrument not provided)  ADL  (Ability to sleep on the affected side, ability to tie an apron, ability to air the futon in the sun) | *Undisclosed* | At postoperative week 1 and 2, movement-associated  pain was reported by 64% and 67% of the patients,  respectively. At postoperative week 4 and 12, pain was  reported by 49% and 44%, respectively, and its frequency  remained high, especially in the BCS group.  Either pain at night or operative wound pain were  reported by 6–12% and 3–15% of the patients at postoperative  week 1 and 2 and postoperative week 4 and  12, respectively. |
| Paolucci  (2021)  Italy | Prospective cohort study | N= 38  *Age, mean ± SD*: 57.40 ± 1.24  *Sx type, n (%):* Total MX + breast prostheses or tissue expanders: 38 (100) | **Rehabilitative treatment group**  Relaxation and breathing exercises, stretching, GH joint ROM EX, cervical pumping, isometric strengthening EX  Initiation: unclear  Freq: 2 times/w  Rehabilitation sessions duration: 1h  Duration: 5w + 2m at home  *Follow-up*: 1 year | Pain intensity  (VAS)  QoL  (EORTCQLQ-C30)  Personality Traits  (MMPI-2) | *Disclosed for cancer population:*  EORTCQLQ-C30  *Disclosed for general population:*  VAS  MMPI-2 | During treatment, 3 patients dropped out for personal problems  None complained about pain |
| Scaffidi  (2012)  Italy | Prospective observational study | N=83  **Group A; n=25**  *Age, mean ± SD*: 49.6 ± 8.8  *Sx type, n:*  *LUMP:* 10 with 7 SND and 3 ALND  *RM:* 15 with 2 SND and 13 ALND  **Group B; n=58**  *Age, mean ± SD*: 52.1 ± 11.9  *Sx type, n:*  *LUMP:* 35 with 26 SND and 9 ALND  *RM:* 23 with 6 SND and 17 ALND | **Group A**  Preoperative information orally + home rehabilitation program (flexibility and elasticity of muscles surrounding shoulder joint)  **Group B**  Preoperative information orally + information materials + PT treatment at hospital: deep breathing, stretching neck muscles, elevation, abd, external and internal rotation of shoulder, flexion and extension of elbow in neutral position + home rehabilitation program  PT at hospital: 1 per day, 30-40 min  Home rehab program: 3 times/ day  *Follow up:* 60 and 180 days | Shoulder arm mobility  (goniometer)  UL function  (Constant and Murley Score)  Presence of LE  (Universal level meter) | *Undisclosed* | - |
| Springer  (2010)  USA | Prospective observational study | N=94  Age, mean ± SD: 53.39 ± 11.80  *Stage, n (%):*  0: 11 (12)/ I: 40 (43)/ II: 30 (32)/III: 13 (14)  *Sx type, n (%):*  BCT: 41(44)/MRM: 50(53)/Simple MX: 3 (3)  Lymph nodes dissection, n (%):  None: 8 (9)/SND: 20 (21)/ALND: 66 (70)  *Systemic treatment, n (%):*  Ch: 57 (61)/RT: 64 (66)/HT: 67 (7) | **UL ROM program**  Flexion, abduction, internal and external rotation  Pre-operative examination: subjects were instructed in a post-operative UL ROM exercise program, and were educated regarding UL LE precautions and physical exercise initiation and progression  Initiation: post-Sx  Reviewed at 1 month  *Follow-up*: 3 post-surgical visits: 1, 3-6 and 12+ months | Pain (NRS)  Bilateral shoulder ROM (goniometer)  Bilateral shoulder strength  (Break testing of UL)  Volume and girth measurements for both UL (Optoelectronic volumeter, Perometer®)  UL function and disability (Upper Limb Disability Questionnaire) | *BC-specific outcome:*  Perometer®  *Undisclosed*  NRS  Goniometer  Break testing of UL  Upper Limb Disability Questionnaire | - |
| Hsieh  (2008)  USA | Pretest and post-test quasi-experimental study | N= 96  Women referred by local oncologists for rehabilitative exercises  *Sx type:* N/A  *Stage*: N/A  **Sx alone; n = 22**  *Age, mean ± SD*: 55.6 ± 11.3  **Sx and Ch; n = 30**  *Age, mean ± SD*: 55.6 ± 11.0  **Sx and RT; n= 17**  *Age, mean ± SD*: 57.2 ± 9.4  **Sx, Ch and RT; n = 27**  *Age, mean ± SD*: 63.1 ± 9.8 | **All groups**  Individualized exercise intervention based on the results of the medical and cancer history, physical examination, and the initial physiologic and psychological assessments  Initiation: immediately following treatment for BC  Exercise sessions in general: 10-min warm-up, 40-min of aerobic exercises, resistance training and stretching and concluded with a 10-min cooldown  Intensity: 40-65% of HR reserve (based on the treadmill assessment results) | Cardiovascular endurance  (Bruce Treadmill Protocol; HR, BP, predicted VO_2max_, time on treadmill and oxygen saturation)  Pulmonary function- FVC, FEV_1_  (Flowmate^TM^ spirometer)  Cancer-related fatigue  (Piper Fatigue Scale) | *BC-specific outcome(s):*  Piper Fatigue Scale  *Undisclosed:*  Bruce Treadmill Protocol  Flowmate^TM^ spirometer | Participants’  adherence to the exercise intervention was approximately  90%, which can be attributed to cancer exercise specialists  who prescribed individualized cancer interventions that fit patients’ circumstances. |
| Petito  (2012)  Brazil | Quasi-experimental, before and after study | N=64  **Mastectomy group; n=43**  *Age, mean ± SD*: 52.2 ± 9.6  *Sx type, n (%):* MRM: 37(86)/  Simple MX:4 (9)/ RMX:2 (5)  **QT group; n=21**  *Age, mean ± SD*: 63.4 ± 9.0 | **Exercise program (both groups)**  9 exercises  Initiation: Postoperative day 1  Duration: 105 post-operative days  Frequency: daily  Rep: 10/exercise  *Phase 1 (1^st^ postop day until drain removal):*  Two stretches for the cervical region, two exercises for movement of the scapular girdle, one for shoulder flexion and one for extension beyond the midline  *Phase 2 (after drain removal until the 105th postop day:*  Three additional exercises: one exercise for flexion and two for abd of the shoulder.  *Follow up:*  Postoperative day 14, 45, 75 and 105 | Shoulder ROM: flexion, extension, abd  (goniometer) | *Undisclosed* | N=22 did not attend the penultimate or last evaluation, n=5 required reoperation.  Satisfactory adherence from the 7th PO until the 75th PO varied from 75.8 to 91.2%. Furthermore, it was observed that the adherence declined according to the recuperation of the ROM. |
| Singh  (2013)  Canada | Quasi-experimental pretest post-test study | N= 73  **Experimental group; n = 42**  *Age, mean ± SD*: 55.1 ± 14.8  *Stage, n (%):*0 or I: 2 (5)/ II: 14 (34)  III: 19 (46)/ N/A: 6 (15)  *Sx type, n (%):*  MRM: 22 (54)/ Simple MX: 7 (17)  BCS: 12 (29)/B recons: 22 (54)  *Systemic treatment, n (%):*  RT: 22 (54)/Ch: 16 (39)  **Comparison group; n = 31**  *Age, mean ± SD:* 62.8 ± 14.1  *Stage, n (%):* 0 or I: 2 (7)/ II: 10 (32)  III: 13 (42)/ N/A: 6 (19)  *Sx type, n (%):*  MRM: 7 (23)/Simple MX: 9 (29)  BCS: 15 (48)/ Brecons: 3 (10)  *Systemic treatment, n (%):*  RT: 14 (45)/Ch: 16 (32) | **Experimental group**  Standardized preoperative ED delivered by 2 trained PT + 2 monitoring visits + PT treatment if needed focusing on teaching self-management strategies, scar tissue massage and AROM and assisted shoulder exercises  *Standardized preoperative education program:*  *-*General postop mobility exercises  -AROM exercises: 10 reps every 4h  -Education on LE  -Scar management  *Follow-up*: 1, 6 and 7 months post-Sx  **Comparison group**  Standardized preoperative ED alone  *Follow-up:* 7 months post-Sx | Arm mobility-Shoulder ROM  (goniometer)  Presence of LE  (Arm CIRC, tape measure)  UE strength  (Manual muscle testing)  UE function  (DASH)  Quality of life  (FACT-B+4)  Postoperative pain  (VAS) | *Cancer-specific outcome*  FACT-G  Arm CIRC  *Disclosed for general population*  DASH  *Undisclosed*  Goniometer  VAS | One participant  did not complete the 7-month follow-up visit  and was therefore removed from the data analysis, leaving  41 participants in the experimental group and 31 in  the comparison group. |
| Rekha  (2020)  India | Quasi-experimental study | N= 20  *Age range*: 40-60  *Sx type*: Unilateral BSx (MX or BCS) within a month | **Group A; n= 10**  Swiss ball exercises + diaphragmatic breathing exercises (10 repetitions)  Duration: 4w; 5 days/w  **Group B; n= 10**:  Stretching exercises + diaphragmatic breathing exercises (10 repetitions)  Duration: 4 w; 5 days/w | Chest expansion (inch tape)  **-**FEV_1_ (computerized spirometer)  **-** Shoulder ROM (goniometer) | *Undisclosed* | - |
| Kilgour (2008)  Canada | Pilot study | N= 40  **Home-based exercise (HBE) group; n = 20**  *Age, mean ± SD*: 50.6 ± 9.3  *Stage, n (%):* N/A  *Sx type*: MRM + ALDN  **Usual care (UC) group; n = 20**  *Age, mean ± SD*: 49.1 ± 5.7  *Stage, n (%):* N/A  *Sx type*: MRM + ALDN | **HBE group**  HB exercise video program that incorporated the exercises and guidelines described in a brochure from CCS  Initiation: 3^rd^ postop day  Duration: 11 days  *Phase 1*  *Day3-9:* Self-A shoulder ROM and flexibility exercises  Frequency: 3 set/day  Sets duration: 5-7 minutes  *Phase 2*  *Day10-14:* Same exercises as Phase 1  Frequency: 2 sets/day  Sets duration: 10-15 min  **UC group**  Received information on diet and skin scare and a 9-page brochure containing stretching and ROM shoulder exercises printed by the CCS, without further instructions | Shoulder ROM  (goniometer)  Shoulder strength  (Manual muscle testing techniques)  Grip strength  (Hand-grip dynamometer)  Forearm CIRC  (Tape measurement)  Frequency of medication intake, VOL of fluid from the axillary drains and self-perceived pain level (CR-10 Pain Scale) and exertion (Borg Scale)  (Diary) | *Disclosed for general population:*  Goniometer  Manual muscle testing techniques  *Disclosed for patient with lower limb LE*:    Tape measurement  Undisclosed:  Hand-grip dynamometer  CR-10 Pain Scale  Borg Scale | HBE group:  -3/16 women discontinued their home program after 4 days: pain around the shoulder joint and swelling of the axillary region.  -2/16 unable to complete: lacked support from their spouse, family  members or friends.  -1/16 swelling around the axillary incision site:  discontinued her program for 4 days.  Explanations to describe adherence rates were: (1) they understood the importance of arm movement (2) the surgical oncologist appeared on the video recommending  the program and (3) there was  strong support from spouses or family members. |
| Baima  (2017)  USA | Feasibility study | N= 60  *Age, mean, stage and systemic treatment*: N/A  *Sx type*: MX or LUMP  **Gr 1- in person teaching; n= 36**  **Gr 2- video-only teaching; n= 24** | **Both groups**  Prehabilitation exercise program and post-Sx shoulder ROM exercises restrictions > 90° until drains were removed  Initiation 1-4w prior to Sx  Frequency: once daily, suspended post-Sx  Sets/Rep: 3/10  ***Participants could resume the same exercises as desired after Sx and after the drains were removed***  **Group 1- in person teaching**  In-person physical demonstration and instructions of supervised shoulder ROM exercises (Codman, scapular squeezes, reach for the pillow)  **Group 2- video-only teaching**  Instruction’s sheet of shoulder ROM exercises and optional exercises video without additional supervision  *Follow-up:* Between 2w and 6 months post-Sx (variable across study participants) | Pain  (NRS-11)  Shoulder abd ROM  (Goniometer)  Postoperative seroma formation | *Undisclosed*  *NRS-11*  *Goniometer*  *Postoperative seroma formation* | *Reasons for attrition:*  -Accessibility of study staff at follow-up oncology appointments to assess shoulder ROM  -Delayed surgical treatment due to Neoadj Ch  -Disease worsening with changes in initial treatment plan  -Computer or Internet access issues  ‘’Several patients had drains in place for several weeks after their Sx, which prevent them from exercising’’ |

***Abd:*** *abduction;* ***Add:*** *adduction;* ***ADL****: Activities of daily living;* ***Adj Ch****: Adjuvant chemotherapy;* ***ALND:*** *Axillary lymph node dissection;* ***AROM****: Active range of motion;* ***AWS****: Axillary web syndrome;* ***BC****: Breast cancer;* ***BCS:*** *Breast conserving surgery;* ***BME:*** *Biomedical education;* ***BP****: Blood pressure;* ***BSx:*** *Breast surgery;* ***Brecons****: Breast reconstructive surgery;* ***CCI:*** *Comprehensive complication index;* ***CCS****: Canadian Cancer Society;* ***CIRC****: Circumference;* ***Ch:*** *Chemotherapy;* ***CG****: Control group;* ***CS:*** *Central sensitization;* ***DASH****: Disabilities of the Arm, Shoulder and Hand;* ***DXA:*** *Dual-energy X-ray absorptiometry;* ***ED****: Education;* ***EORTC QLQ:*** *European Organization for Research and Treatment of Cancer quality of life questionnaire;* ***FACT-B****: Functional Assessment of Cancer Therapy-Breast;* ***FEV_1_:*** *Forced expiratory volume in one second;* ***FVC:*** *Forced vital capacity;* ***HB****: Home-based;* ***HR****: Heart rate;* ***HRQOL:*** *Health-related quality of life;* ***HT:*** *Hormonotherapy;* ***IG****: Intervention group;* ***IORT:*** *Intraoperative radiotherapy;****IPC****: Intermittent pneumatic compression;* ***IVLD****: Interlimb volume difference;* ***KBR:*** *Kinect based rehabilitation;* ***LE****: lymphedema;* ***LUMP****: Lumpectomy;* ***MFBIA****: Multi-frequency bioimpedance;* ***min****: minutes;* ***MLD****: Manual lymphatic drainage ;* ***Mobs:*** *mobilizations****; MRM****: Modified radical mastectomy;* ***MT:*** *Manual therapy;* ***MX****: Mastectomy;* ***N/A****: Not available;* ***Neoadj****: Neoadjuvant;* ***NRS:*** *Numeric Rating Scale;* ***OA****: Operated arm;* ***OT****: Occupational therapist;* ***PCM****: Pectoral muscle-conserving mastectomy;* ***PENS:*** *Percutaneous electrical nerve stimulation;* ***PMX:*** *Partial mastectomy****; PMMX:*** *Partial modified mastectomy****; PNE:*** *Pain neuroscience education* ***PPMP:*** *Persistent post-mastectomy pain;* ***PROM****: Passive range of motion;* ***PT****: Physical therapy(ist);* ***QT:*** *Quadrantectomy;* ***RCT:*** *Randomized controlled trial****; RM:*** *Repetition maximum;* ***RMX:*** *Radical mastectomy;* ***ROM:*** *Range of motion;* ***RPE:*** *Rated Perceived Exertion;* ***RT:*** *Radiotherapy;* ***SD****: Standard deviation;* ***SDQ****: Shoulder Disability Questionnaire;* ***Self-A****: self-administered;* ***SIP****: Sickness Impact Profile;* ***SNB:*** *Sentinel lymph node biopsy;* ***SND****: Sentinel lymph node dissection;* ***ST****: Soft tissue;* ***Sx:*** *Surgery;* ***TKS****: Tampa Kinesiophobia Scale;* ***UE:*** *Upper extremity;* ***UL:*** *Upper limb;* ***VAS****: Visual Analog Scale;* ***VOL****: Volume;* ***w****: week;* ***WLE****: Wide local excision;* ***6MWT****: 6-Minute Walk Test*

Supplementary Table 2: Description of the interventions of the included studies (based on TIDieR checklist)

| **First author (Year)** | **Intervention’s name**  **(Comparison arm)** | **Materials and procedures** | **Provider(s) and modes of delivery** | **Location, schedule, and intervention’s characteristics** | **Tailoring and modifications** | **Adherence** |
| --- | --- | --- | --- | --- | --- | --- |
| Ammitzbøll  (2019) | **PRT**  (Usual care) | *Materials:* Dumbbells and resistance bands were provided for HB EX sessions  *Procedures:* EX involved the major muscle groups in the UL, lower limb, and core | PTs with a short  introduction to BC surgery, treatment, rehab needs, and complications  *P1*: in group, SPV  *P2:* Mixed (in group + self-A) | *Location:*  *P1*: Study hospital *P2*: HB  prompted by weekly mobile phone text messages for EX reporting.  *P1* (*w1-20)*  Initiation: 3^rd^ post-op w  Freq: 3 days/w  Int: started at <60% 1-RM  *P2 (w21-50)*  Initiation: after P1  Freq: 3 days/w  Duration:50-55 min (10-15-min warm-up, 40 min of resistance training)  REP/Load/Sets:  w1-4 :15-20/25 RM/2-3  w5-8: 15-17/20 RM/3  w9-12: 10-12/15 RM/3  w13-50: 10-12/10-12 RM/3 | *Individualized:*  Monthly tests by supervisors guided individual  progression in load and intensity  *Modifications*: NR | *Adherence*: The rate of intervention adherence to 2 or more EX sessions per week in phase 1 was 79%, and 85% reported exercising in more than 80% of the weeks in phase 2 (results not shown).  *Dropouts:*  IG: hospital anxiety (n=1), cancer recurrence (n=1), deaths (n=3)  CG: pain due to scar tissue (n=1), recent breast recons (n=1), no time or resources (n=2), cancer recurrence (n=3) |
| Ammitzbøll  (2019) | **PRT**  (Usual care) | *Materials:* Dumbbells and resistance bands were provided for HB EX sessions  *Procedures:* EX involved the major muscle groups in the UL,  lower limb, and core | *Providers*: NR  Phase 1: in group  Phase 2: Mixed (in group + self-A) | *Location:*  Phase 1: study hospital  Phase 2: HB, only prompted by weekly mobile phone text messages for EX  reporting.  *P1* (*w1-20)*  Initiation: 3^rd^ post-op w  Freq: 3 days/w  Int: started at <60% 1-RM  *P2 (w21-50)*  Initiation: after P1  Freq: 3 days/w  Duration:50-55 min (10-15-min warm-up, 40 min of resistance training)  REP/Load/Sets:  w1-4 :15-20/25 RM/2-3  w5-8: 15-17/20 RM/  w9-12: 10-12/15 RM/3  w13-50: 10-12/10-12 RM/3 | NR | *Adherence:* NR  *Lost to follow-up:*  IG: death (n=3),  logistics (n=3), unable to contact (n=1), hospital anxiety (n=1), personal reasons (n=1) and cancer recurrence (n=1)  CG: unable to contact (n=3), other surgery (n=1), no time (n=2), personal reasons (n=3), withdrew consent (n=1), pain from scar (n=1), cancer recurrence (n=3) |
| Anderson  (2012) | **EX program + LPM**  (UC) | *Materials:*  LPM: Instructions and care for the affected arm and hand, video-taped tutorial of arm strengthening and lymph flow EX and instructions to wear a compression sleeve  UC: ED (LE awareness, recommendations about PA nutrition and for improving function and strength  EX program: If participants chose HB EX (*P3*), EX specialist contacted them during this time to discuss adherence/barrier issues, answer EX-related questions, and to modify EX prescriptions as needed.  *Procedures:*  EX program: aerobic warm-up, moderate to hard walking, upper and lower body strength training and stretching EX | Individual certified by the ACSM as an EX specialist and by the AHA’s ACLS led the EX sessions  *P1*: SPV  *P2*: Mixed (self-A + SPV 1/w)  *P3*: self-A | *Location:*  *P1*: CRC/*P2*:CRC + HB/ *P3*:HB  LPM  Initiation: 4-12w post-op  EX program  Initiation: after LPM  Freq: 2 days/w to once/w Int: weights were increased weekly by approximately 1–2.5 lbs on upper body EX and 1-5 lbs on lower body EX.  Duration: 65 min (5-min warm-up, 30-min of walking, 20 min of strengthening EX and 10 min of stretching)  REP/Load: Up to 12/50% 1 RM | The sessions were customized to meet baseline levels of strength and function.  *Modifications*: NR | *Adherence:* Participants completing 71.2% of all prescribed EX sessions with a range of 0–97%. 61% of participants attended more than 75% of sessions and only 13% attended less than 50% of sessions  *Strategies used to ↑ compliance:*  -Individual theory-based behavioral reinforcement sessions and a monthly group session to discuss barriers and obstacles in implementing an EX program  -Monitoring of progress  -Incentives for participation  -Transportation provided |
| Bendz  (2002) | **Shoulder EX program**  (Early vs delayed) | *Materials:*  (Preoperative instructions) were advised to use the arm as much as comfortable but to avoid lifting and carrying heavier items  *Procedures:* Included intermittent hand contractions and comprehensive ROM program | Specialized PTs  Mixed:  1^st^ pod-14^th^: HB  From 14^th^ pod: SPV | *Location:*  1^st^ pod-14^th^: HB  From 14^th^ pod: outpatient clinics  Group A; Initiation: 1^st^ pod  Group B; Initiation: 2^nd^ post op w  Both groups  Freq: 3 times/d  Int: NR  REP/Sets: 5/NR  Duration: NR | *Tailoring:*  NR  *Modifications: NR* | *Adherence:* NR  *Dropouts:* death (n=5),  moving from the area (n=6), other diseases (n=3), surgery on the opposite side (n=3)  and personal reasons (n=8) |
| Beurskens  (2007) | **Exercise therapy**  (Usual care) | *Materials:*  Patients assigned to the CG received a leaflet flyer with advice and EX for the arm/shoulder for the first weeks following surgery and had no further contact with a PT.  *Procedures:*  NR | PTs  Received information about t_x_ guidelines (EX for arm/shoulder, posture correction, coordination EX, EX for muscular strength, improvement of general physical condition, EX to prevent LE and instruction for soft tissue massage of the scar if required  One-on-one sessions | *Location:*  Private PT office of their own choice  Initiation: 2^nd^ post-op w  Freq: 1-2/w for the first 3w and then once a fortnight or less + 10 min of home EX daily  REP/Sets: NR  Duration: 3 months (9 t_x_ sessions, determined because of insurance coverage) | *Tailoring:*  Unclear (PT had to comply with specific t_x_ regimes but judged the number of PT sessions that was sufficient to improve shoulder function)  *Modifications:* NR | *Adherence*: NR  Thirty women completed the study protocol. In the follow-up period one patient from the control group died before the last assessment |
| Box  (2002) | **PCMP**  (Usual care) | *Materials:*  IG: progressive educational strategies (LE risk factors, discussion of the lymphatic system, signs of arm swelling, risk-minimization strategies)  CG: received an EX instruction booklet  *Procedures:* Preop  assessment and explanation with postop reviews to monitor shoulder ROM, progress EX program, LE awareness ED and individualized intervention as required. | PTs  One-on-one sessions | *Location*:  Outpatient Breast Surgical Clinic at Royal  Brisbane Hospital  Initiation: unclear (post-BSx)  Freq: NR  REP/Sets: NR  Duration: NR | *Tailoring:* Individualized intervention was provided as required  *Modifications:* NR | *Adherence*: NR  *Dropouts: 9*%; relocation interstate or overseas (2), unavailability for final  assessment (1) and death (3)  *Strategies used to ↑ compliance:*  -Positive reinforcement by PT |
| Box  (2002) | **PCMP**  (Usual care) | *Materials:*  IG: progressive educational strategies (LE risk factors, discussion of the lymphatic system, signs of arm swelling, risk-minimization strategies)  CG: received an EX instruction booklet solely  *Procedures:* NR | PTs  One-on-one sessions | *Location*:  Outpatient Breast Surgical Clinic at Royal  Brisbane Hospital  Initiation: unclear (post-BSx)  Freq: NR  REP/Sets: NR  Duration: NR | *Tailoring*: NR  *Modifications*: NR | *Adherence:* NR  Reasons for the 9% loss over in the 2-year period were relocation  interstate or overseas (2), unavailability for final assessment (1) and death (3) |
| Cho  (2016) | **PT + MLD**  (PT) | *Materials*: For strengthening EX, a Thera‐Band was used  *Procedures:*  EX: UE strengthening and stretching combined ST and shoulder girdle mobs and PROM EX  MT: Gentle circular mobs of tight and stiff tissues of the chest wall and antecubital fossa with full hand or 2-finger contact and longitudinal tissue stretch with the patient’s arm in possible range of abd  MLD: light form of circular  massage and stationary circle, pumping and scooping, and rotary mvts performed with varying degrees of pressure. | EX: All EX were performed under the supervision  of PTs  MLD: 2 LE therapists (1^st^ post-op w)  Self-A (2^nd^ to 4^th^ post-op w) | *Location*: Department of Rehabilitation Medicine of Asan Medical Center, Seoul, Korea  Initiation: ≥ 4w after BSx  PT program  Freq: 3 times/w  Int: 60-80% 1RM  REP/Sets: 10/3  Duration: 4w  MLD  Freq: 5 days/w  Duration: 30 min each session for 4w | *Tailoring:* Treatment intensity was progressively increased from  comfortable to mild discomfort within the tolerable range  *Modifications:* NR | *Adherence*: NR  *Dropouts*:  Seven patients were unable to complete  the final evaluation (reasons not stated): 41 patients completed the study protocol |
| Cinar  (2008) | **ROM EX + PT program**  (HB EX program) | *Materials:*  Both groups were informed about skin care and issues that they should take care during ADL  *CG:* Postoperative EX forms to perform at home  *Procedures:* 1^st^ pod: hand/elbow AROM EX;  2^nd^ pod: Isometric hand/forearm EX; 3^rd^ and 4^th^: AROM shoulder EX  PT program: Passive stretching, strengthening and ROM EX (pendulum, wall climbing, overhead pulley, horizontal abd, posture, wand, dorsal strengthening, and stretching EX for levator scapula) | Each EX was thought  by PTs until it was performed properly  15 SPV and individual sessions and 8w self-A | *Location*: 15 sessions in the Department of Physical  Medicine and Rehabilitation, Ankara Numune Training and Research Hospital +8w HB  Initiation:  ROM EX: 1^st^ pod  PT program: > drains removal  Freq: NR  Int: NR  REP/Sets: NR  Duration: unclear (15 SPV PT sessions + 8w HB) | *Tailoring:* NR  *Modifications:* NR | NR |
| De Groef  (2017) | **PT + MT**  (PT + placebo) | *Materials: NR*  *Procedures:*  PT program: passive mobs, stretching and transverse strain of pectoral muscles, scar tissue massage, EX schemes to restore and improve muscle flexibility, endurance and strength, posture and mvts control and shoulder AROM  MT: manual myofascial  release techniques on (1) active myofascial trigger points at the UL region and (2) myofascial  adhesions in the pectoral, axillary and cervical  regions, diaphragm, and scars. The pressure applied  by the therapist hands proceed from the superficial to  deep layers of the myofascial tissues. Where a  resistance was felt, the barrier was softly maintained  until a release was felt.  Placebo: static bilateral hand treatment at the upper body and arm | MTs  Both MTs had a master’s degree in Rehabilitation  Sciences, one with four years of clinical experience  and one just graduated. The latter underwent 2  m of training before the start of the study. 2 other MTs performed the MT/placebo interventions. Both  MTs were educated in MT and  had several years of clinical expertise.  Individual sessions | *Location*: Department of Physical Medicine and  Rehabilitation of the University Hospitals Leuven.  Initiation:  PT program: 1^st^ pod  MT: 2 m post-op  Placebo: 2m post-op  Freq (PT): 2/w to 1/w after 2m  Freq (MT and placebo): 1/w  Int: NR  REP/Sets: NR  Duration:  PT: 4m (30 min each session)  MT: 2m  Placebo: 2m (30 min each session) | *Tailoring:*  PT program was standardized  *Modifications*: NR | *Adherence:* NR  *Dropouts:*  *IG:* not able to come to the hospital (n=3)  *CG:* No dropouts |
| De Rezende  (2006) | **ROM EX program**  (Directed vs Free ROM) | *Materials:* NR  *Procedures:*  *Directed G:* The PT technique used was kinesiotherapy based on spontaneous EX including movements for flexion, extension, abd, add and internal and external rot of the shoulder, either isolated  or combined (Regimen of 19 EX; pictures and description provided)  *FROM G*: Same ROM EX program without a previously defined sequence or number of repetitions | PTs and students trained in the specialization  EX were practiced in groups of 5-20  women | *Location:* Physiotherapy Outpatient Center  of CAISM-Unicamp  Initiation: 1^st^ pod  Freq: 3 days/w  Int: NR  REP/Sets: 10/NR  Duration: 42 days (40 min each session) | *Tailoring:*  The course followed in these EX  depended on PT ability and experience.  *Modifications:* NR | *Adherence:*  The groups were homogeneous with respect to the  number of PT sessions performed (max 18):  Directed G 13.83 ± 3.05 sessions  FROM G :13.19 ±  1.9 sessions |
| Devoogdt  (2018) | **ED + Exercise therapy +MLD**  (ED+EX therapy) | *Materials:*  *Both G*: Received information about the prevention of LE (elevate the arm in case of heaviness, avoid lifting heavy objects, use the arm in ADL, avoid limb constriction, avoid extremes of T°, apply skin care, and avoid an increase in body weight) + EX therapy (mobilizing EX) during hospitalization  *Procedures:*  *EX:* passive mobs of the  shoulder; stretching and transverse strain of the breast muscles, scar tissue massage; and active mobilizing and stabilizing EX*.*  MLD: neck and ALN were emptied, axilloaxillary anastomoses at the breast and back and lymphatics at the lateral side of the shoulder (Mascagni pathway) were stimulated, and the arm and hand were drained from proximal to distal.  *CG*: Protocol described above without MLD | All t_x_ (information, EX therapy and MLD) were  performed by 4 therapists. Two of them had undergone  MLD training with the Leduc method, and the two others  had undergone MLD training with the Vodder method  Individual sessions | *Location:* Department of  Physical Medicine and Rehabilitation of the University Hospitals  Leuven.  Initiation:  EX: during hospitalization  MLD: 1w > drains removal  Freq (EX): 2/w to 1/2w  Freq (MLD): 1-3/w to once/w  REPS/Sets: NR  Duration: 6m (30 min each session)  MLD: 20w | *Tailoring: NR*  *Modifications: NR* | *Adherence:* Reasonable compliance  IG: 11 participants (15%) received 23-29 MLD  sessions, 26 participants (36%) received 30-35 sessions, and  36 participants (49%) received > 35 sessions.  Reasons for absence: illness related  to Ch and/or RT, problems with transport, holiday, and illness from other causes |
| Feyzioglu  (2020) | **Kinect-based rehab**  (PT group) | *Materials:*  Both groups: Received imitations for shoulder ROM amplitudes, weightlifting, jumping and running up to 6w post-op  *Procedures:*  *Both G:* HB EX program consisting of breathing, ROM and pumping EX (w0-2)  *KBR G:* Xbox 360 Kinect video game program (requiring AROM of the UL) combined with tissue massage and passive mobs  *PT G:* UE PT program (shoulder ROM, stretching and strengthening EX) including scar tissue massage and mobs | The treatment program (tissue massage and mobs) was performed by  experienced PTs  Mixed (SPV + HB) | *Location:* Unclear  Initiation:  General EX: 1^st^ pod  KBR and PT program: 2^nd^ post-op w  Freq (both groups): 2 days/w  Int: NR  REPS/Load/Sets: provided in Table 1, varied according to the EX  Duration: 6w (45 min each session) | *Tailoring*:  EX: standardized program  *Modifications:* NR | *Adherence*: NR  *Dropouts:*  KBR G: refused the intervention (n=1)  PT G: refused the intervention (n=1), Ch AEs (n=1), new metastasis focus (n=1) |
| Kilbreath (2012) | **EX group**  (Usual care) | *Materials:*  *Both G*: Postoperative care which included written information outlining postop arm EX they were to perform, literature on prevention of LE (avoiding lifting heavy objects, prolonged activities such as scrubbing, insect bites, injections, BP test on the at OA). For weekly SPV sessions, free weights were used; for the HB program, women were provided with a Thera-band  *Procedures:*  *EX group:* EX sessions of resistance training and passive stretching for shoulder muscles + home program of resistance training and stretching (positions maintained 5-15 mim) | PTs and/or OTs  Mixed: SPV sessions + HB program | *Location:* HB + unclear (hospital?)  Initiation: 4-6 w post-op  Freq: once/w  Int: instructed to work towards a target of about 15  (Hard) on the Borg Effort Scale  REPS/Sets: 8-15/2  Duration: 8w | *Tailoring:*  Adaptive strategies were used if women were unable to achieve > 90° elevation  *Modifications:* NR | *Adherence:*  *EX group:* median  number of sessions attended was 7 out of, with adherence to the SPV training 78%  *HB program*: The minimum requirements were 40 sessions of  stretching and 24 sessions of resistance training over the 8 weeks; mean compliance: 90%. Seven women completed less than 75% of their stretching sessions and 4 women completed less than 75% of their resistance training sessions |
| Kilbreath (2006) | **EX group**  (Usual care) | *Materials:* EX booklet was given to patients before hospital discharge; a Theraband was used for strengthening EX  *Procedures:*  *Usual care G:* Monitoring by a breast care nurse, may be seen by a PT to review UL EX and by an OT who discussed prevention of LE before discharge  *EX group:* Usual care + shoulder ROM, strengthening (focused on shoulder flexors, abductors, and external rotators) and stretching EX  (was held passively for 5 min on day 1 and progressed up to 15 min over the next 2w) | PTs  Mixed (HB once/w + SPV once/w) | *Location:* HB + unclear (hospital?)  Initiation: 4-5w post-op  Freq: 2/w  Int: Low to ‘’somewhat hard’’ on the Borg Scale  REPS/Sets: 8-12/2  Duration: 8w | *Tailoring*:  EX intensity progressed according to participants perceived exertion (Borg scale)  *Modifications:* NR | *Adherence*: NR |
| Lauridsen  (2005) | **Standart t_x_ + PT**  (Standard t_x_ + delayed PT) | *Materials:* NR  *Procedures:*  *Standard t_x_:* daily demonstrations and instructions in shoulder and vein pump EX during the first postop w  *PT program:* relaxation and strengthening EX, combined to vein pump therapy and instruction in stretching of scar tissue | Standard t_x_: Instructions were given by PTs and the patients were encouraged  to continue exercising after hospital discharge  PT program: To ensure that the patients received uniform t_x_ the same two PTs handled all sessions | *Location:* NR  Initiation:  Group A: 6^th^ to 8^th^ post-op w  Group B: 26^th^ post-op w  Freq: 2 days/w  Int: NR  REPS/Sets: NR  Duration: 6w (60 min each session) | *Tailoring:* No individual therapy was applied  *Modifications:* NR | Ad*herence:* NR  *Dropouts:*  14/139 (10%); including  two patients who died and two patients who had terminal disease, disabling them in attending the final follow-up |
| Pace do Amaral  (2012) | **MT + UL EX**  (UL EX) | *Materials:* Women were advised to take precautions to prevent LE of  the operated UL  *Procedures:*  *UL EX: (1^st^-3^rd^ pod)* forward flexion, external rotation and shoulder abd; (> 3^rd^ pod- 1m) 19 movements of flexion, extension, abd, add, internal, and external rotation of the UL, alone or combined  *MT*: Scapular and GH mobs and therapeutic massage. Techniques used for mobs were gliding, oscillation, and traction for the GH joint; and add, abd, elevation,  depression, and internal and external rotation for the scapula. Therapeutic massage (friction maneuvers and deep gliding) was used in the presence of wound adherence or lymphatic cording | PTs  UL EX: Providers unspecified. All women underwent an EX protocol in a group setting  MT: Seven PTs were trained by the main investigator to perform the technique.  Individual sessions | *Location:* Division of Physical Therapy of the Women’s Integrated  Health Care Center of the University of Campinas  Initiation: 1^st^ pod  Freq (EX): 3/w  Freq (MT): 2/w  Int: NR  REPS/Sets: 10/1  Duration:  EX: 1m (45 min each session)  MT: 1m (20 min each session) | *Tailoring:* NR  *Modifications:* NR | Ad*herence:* NR  *Dropouts:*  *MT+UL*: 40/65 (62%)  *UL:* 36/66 (55%); moved away or death |
| Petito  (2014) | **EX protocol**  (Early vs delayed) | *Materials:* Participants were supported by an illustrated manual prepared and validated for the study. All the patients received guidance regarding the management of the drain at home, and those in the EG received guidance and performed the initial EX under the supervision of the researcher  *Procedures:* NR  (Was composed of 9 EX) | HB  (Guidance was provided by the principal investigator if needed) | *Location:* HB program  Initiation:  EG: 1^st^ pod  DG: > drain removal (postop day 7-10, mean postop day: 9)  Freq: once/day  Int: NR  REPS/Sets: NR  Duration: 15w (105 days) | *Tailoring:* NR  *Modifications:* NR | Ad*herence:* NR  *Dropouts*:  *EG:* 8/48 (17%)  *DG*: 9/46 (20%); lost to follow-up, death, returned to surgery |
| Sagen  (2009) | **Physical therapy program**  (AR vs no restrictions) | *Materials:* Each patient in the AR group was given standard detailed information on the  restricted activities in a sealed envelope.  *Procedures:* Moderate progressive resistance EX +  6 different standardized passive manual techniques emphasizing flexibility and light massage of the affected shoulder, arm and scar. | PTs  SPV sessions | *Location:* Outpatient clinic  Initiation: Unclear  Freq (AR): once/w  Freq (NAR): 2-3 days/w  Int: NR  REPS/Sets: 15/NR  AR: restricted activities of the OA avoiding heavy weights (>3kg)  NAR: 0.5kg the (0-2 w) ↑ individually after  Duration: 6m (45 min each session) | *Tailoring:* PT program was standardized  *Modifications:* NR | Ad*herence:* Adherence to the allocated rehabilitation programs was 83% in the NAR group and 89% in the AR group.  *Dropouts*  NAR: 36/104 (35%)  AR: 16/104 (15%); death, moved away, not available, refused to participate, too frail or ill, other Sx, lost to follow-up |
| Schultz (1997) | **Shoulder EX**  (Early vs delayed) | *Materials:* NR  *Procedures: P*atients  were instructed to do active shoulder EX to  regain full range of motion, especially in the directions  that may be difficult postoperatively, i.e., anteflexion, abd, and rotation | PTs  EX were performed under individual guidance of PTs before discharge; HB > discharge | *Location:* Outpatient department; HB  Initiation:  EG: 1^st^ pod  DG: 1^st^ post-op w  Freq: 3 times/day  Int: NR  REPS/Sets: NR  Duration: NR | *Tailoring:* NR  *Modifications:* NR | Ad*herence:* NR |
| Siedentopf (2013) | **Yoga**  (Early vs delayed) | *Materials:* NR  *Procedures:* Each lesson started with lying postures and the gradual mobs  of arms and legs to encourage flexibility and strength. Various breathing EX followed to allow participants to feel their chest wall expanding. During the dynamic part of the EX, a range of standing and sitting positions were shown. At the end of the class, participants were led through a series of concentration EX to help them relax and become aware of the effects of the yoga EX | Two certified yoga  teachers, one demonstrating the asana, the other  teacher assisting the participants with props | *Location:* Hospital’s gym  Initiation:  EG: 1^st^ pod  DG: 5^th^ post-op w  Freq: 2/w  Int: NR  REPS/Sets: NA  Duration: 5w (75 minutes each session) | *Tailoring:*  Positions variations tailored to the needs of patients were included as needed. The teachers were careful to ensure that participants  performed each EX according to their own optimal degree of Int.  *Modifications:* The yoga class encompassed a  large variety of movements, all of which were easily doable in  their modified versions. The modifications were created to meet post-op needs and to help during the period following therapy when the women were less flexible | Ad*herence:*  Various reasons were cited for canceling classes, but most patients gave no reason (7 women in the EG, 8 women in the DG). The 2^nd^ most frequently cited reason was ongoing adjuvant t_x_  The average number of attended yoga classes was 7.62 (SD ± 3.78) in the EG and 7.25 (SD  ± 4.21) in the DG with a range of 0–11 in both groups (p = 0.89,  Mann-Whitney U-test). No statistical difference was found in the numbers attending classes. |
| Temur  (2019) | **SMLP + EX + SLD**  (Usual care) | *Materials:* (SMLP) Training booklet containing information about mechanisms and risk factors of LE and about prevention interventions were given to patients in the IG and distributed at the end of the study for patients in the CG  *Procedures:*  EX: Hand squeezing EX with a medium-level stress ball + active and passive arm EX (pictures and description provided)  SLD: deep diaphragmatic  breathing EX, neck drainage, unaffected side axillary  drainage, affected side axillary drainage, and UE drainage. Before the massaging started, the patients performed breathing EX | *Providers*: NR  EX: Mixed (SPV then switched to HB)  SLD: Self-A | *Location:* Adult Hospitals General Surgery Department + HB  Initiation: 1^st^ pod  Freq (EX): 3-6 times/day; gradually increased to 10  Freq (SLD): twice/day  Int: NR  REPS/Sets: varied according to the EX and timing (see table 1)  Duration: 6m (30-60 min per EX session) | *Tailoring:*  The minimum amount of EX required for the patient were determined by the doctor and nurse taking into consideration the physical condition  of the patient, and his general well-being during the postop period.  *Modifications:* NR | Ad*herence:* NR  *Dropouts:*  IG: did not follow the research program (n=2), wound site revision in the post-op period (n=3), developed skin reaction after RT (n=1)  CG: did not follow the research program (n=1), wound site revision in the post-operative period during the research (n=2), developed skin reaction after RT (n=2) |
| Teodózio  (2020) | **ROM EX**  (Free vs restricted) | *Materials:* Patients in both  groups received an instructional leaflet and were information not to perform rough movements or  lift high weights in these first 30 postop days  *Procedures:*  Free G*: A*ctive UL mvts with ROM over 90° for flexion and abduction of shoulder from the 1st pod  Restricted G: Active UL movement with ROM restricted to 90° from the 1st POD until removal of all surgical stitches, when a ROM over 90° was allowed (pictures and description are not provided) | HB  (Participants were instructed  by the PTs regarding the UL EX to be performed) | *Location:* HB  Initiation: 1^st^ pod  Freq: 3 times/day (at least once/day)  Int: NR  REPS/Sets: NR  Duration: 4w | *Tailoring:* NR  *Modifications:* NR | Ad*herence:* Adherence  was considered when the EX was performed three times a day or at least one time a day on some days during  the intervention weeks (results not provided)  *Dropouts:* 0,9%; non-return to the wound-dressing clinic (*n* = 1), hospitalization for reasons other than a surgical approach (*n* = 2) and changes to the treatment protocol (n=1) |
| Testa  (2014) | **PT program based on the latest guidelines**  (Based on old guidelines) | *Materials:* NR  *Procedures:*  IG: (From 2^nd^ pod) active flexion, extension, lateral flexion and rotation of the C-spine + passive and active assisted caution mobs of hand, wrist, elbow (flexion, extension, pronation, supination). (From 3^rd^ pod) Arms passive EX of flexion, abd, add and circumduction + active internal, external rotation. (Once drains were removed) 20 PT sessions including EX postural and stretching EX + active movements of abd, flexion, internal and external rotation of glenohumeral joint  CG: No early physical rehabilitation treatment, neither has received instructions from PTs | PTs  Individual and SPV sessions | *Location:* Breast Unit of ‘’San Giuseppe Moscati’’ hospital  Initiation: 2^nd^ pod  Freq: 5 times/w  Int: NR  REPS/Sets: NR  Duration:  (EX): 40 minutes  (PT sessions): 60 minutes | *Tailoring:* Patients starting complaining pain was the limit beyond PTs did not go during EX  *Modifications:* Patients of the CG from March 2010 to August 2010 were treated following the old rehabilitation guidelines. Patients of the IG from Sept 2010 to February 2011 were treated adopting EX described in the guidelines for rehabilitation in BC patient published on 2005 by the Italian society of senology | Ad*herence:* NR |
| Todd  (2008) | **Shoulder EX**  (Early vs delayed) | *Materials:* NR  *Procedures:*  Delayed G: EX that limited movement of the arm below 90◦ in all planes of movement over the 1^st^ week, followed by the introduction of a full range of shoulder movement in the 2^nd^ week.  Early G (control): Full shoulder  mobs (i.e., movement above 90◦) was introduced immediately, with vigorous arm and shoulder EX  started within the first 2 pod. | Nursing staff was responsible for the delivery of exercise sessions in clinical setting  The EX program was SPV during inpatient stay (average 7 days).  Group sessions (number of participants per group not indicated)  + HB | *Location:* Clinical setting (NR) + HB  Initiation:  DG: 2^nd^ pod (limited ROM), 2^nd^ w (full ROM)  EG*:* 2^nd^ pod (full ROM)  Freq: 4 times/day until full shoulder ROM was restored and then once/day for the 1^st^ post-op year  Int: NR  REPS/Sets: 3-4/1  Duration: 1 year (10 min each session) | *Tailoring:* Standardized EX program  *Modifications:* NR | Ad*herence*  There was regular telephone contact with all study participants, and the majority of women (73%, n = 85) claimed that they had adhered to their EX program throughout the year  *Dropouts:*  7 participants (6%) withdrew during the first year (death, moved away, refused the allocated intervention) |
| Torres  (2010) | **PT + ED strategy**  (ED strategy) | *Materials:* (ED strategy) Instruction with printed materials about the lymphatic system, concepts of normal load versus overload, the source of 2nd lymphoedema,  the identification of possible precipitating factors, and the four categories of interventions to prevent  2nd lymphoedema (avoidance of trauma or injury, prevention of infection, avoidance of arm constriction, and use and EX of the arm) together with individual strategies for implementing  these measures  *Procedures:* PT: included MLD technique used for the t_x_ of post-op oedema (thorax, breast,axilla, and upper arm of affected side), using a modification of the strokes described by Leduc, progressive massage of the scar, stretching EX for levator scapulae, upper trapezius, pectoralis major, and medial and lateral rotators muscles of the shoulder; and progressive  active and action assisted shoulder EX, started in conjunction with functional activities and proprioceptive neuromuscular facilitation EX without resistance | PTs  Each group had one PT, who carried out  all interventions. The PTs had more than five years’ experience in the t_x_ of vascular diseases  using LD | *Location:* unclear  Initiation: 3-5 days after hospital discharge  Freq: 3 times/w  Int: NR  REPS/Sets: NR  Duration: 3w | *Tailoring:* NR  *Modifications:* NR | Ad*herence:* NR  *Dropouts:*  Overall, 116 women completed the follow-  up assessments; 59/60 in the IG and 57/60 in the CG (no reasons provided) |
| Wingate  (1989) | **Physical therapy**  (No intervention) | *Materials:* Instructions with printed materials were provided for home EX program.  *Procedures:*  IG: Motor and sensory status, active hand, wrist, elbow and postural EX, active and active assisted shoulder EX, functional activities and PNF (EX descriptions or pictures were not provided)  CG: Untreated group with no physical therapy | *Providers:* NR  SPV upon hospital discharge + HB | *Location:* Clinical setting (unclear) + HB  Initiation: 1^st^ pod  Freq: 2 times/day  Int: NR  REPS/Sets: NR  Duration: 8w minimum | *Tailoring:* NR  *Modifications:*  NR | Ad*herence:* NR |
| Zhang  (2016) | **EX program**  (EX + MLD) | *Materials:* Patients in both groups received ED regarding the risk of post-op complication sand the importance of medical intervention and self-management.  *Procedures:* EX: Began with passive EX within the first 7 days after surgery, before removal of the drainage tube. Between removal of the drainage tube and the surgical sutures at 7–30 days, the EX progressed to localized active EX on the affected UL. After removal of the surgical sutures, extensive active EX involving the affected shoulder was undertaken; MLD: Each session was divided into three sequential steps to activate lymph vessels, to soften scar tissue, and to stimulate lymph drainage (details provided in the article) | *Providers: unclear*  Mixed (SPV + HB)  MLD: Self-A | *Location:* San Yat-Sun  University Cancer Center + HB  Initiation:  EX: < 24h surgery  MLD: > sutures removal  Freq: 3 times/day  Int: NR  REPS/Sets: NR  Duration: 6m  EX sessions: varied between 15 to 30 min  MLD: 30 min | *Tailoring:* NR  *Modifications:* NR | Ad*herence*: NR |
| Zhou  (2019) | **UL EX**  (Usual care) | *Materials:* NR  *Procedures:*  IG: Progressive UL EX and PMRT (description provided in table 1); CG: Routine nursing care (surgery district nursing, drainage tube nursing, routine health ED, physical EX, vital sign monitoring and post-surgery complications) | Nursing staff  In individual or group format  until the patients could perform the EX and training  freely and easily without discomfort.  Mixed: If the patients had difficulties in performing the EX and training following surgery, the nursing staff would help and  encourage the patient via one-to-one supervision (in  hospital) or home visiting (discharged from hospital)  until the patients could do the EX and training independently | *Location:* A general hospital in Xi’an, China  Initiation: before surgery  Freq (EX): 3-7 sessions per day  Freq (PMRT): twice/day  Int: NR  REPS/Sets: NR  Duration: 6m (varied between 10 to 30 min per session) | *Tailoring:* For those patients without abnormal condition and complications after surgery, EX were performed in a step-by-step modality. If the patients had complications or abnormal conditions after surgery, the  duration, freq, and shoulder ROM would be decreased accordingly  *Modifications:* NR | Ad*herence:* All intervention group patients completed the EX and training, with AEs and a compliance of 100%. |
| Zimmermann  (2012) | **EX + MLD**  (EX + self-MLD) | *Materials:* NR  *Procedures:*  IG: (EX) PT program including EX of limb and chest PT (MLD) Massage strokes were applied to the side of the edematous limb, starting at the base of the neck and then progressing to the affected limb. The massage was always directed proximally from the  upper arm to the axilla, and then from the hand to the elbow. Finally, the whole limb was massaged from the distal to the proximal extremity  CG: Applied self-drainage from modification of the method described by Földi and Strönbenreuher | PTs  IG: SPV individual sessions  CG: Mixed (EX SPV, MLD self-A) | *Location:* NR  Initiation:  EX: 2^nd^ pod  MLD: 14^th^ pod  Freq (EX): NR  Freq (MLD): 5 sessions/ week  Int: NR  REPS/Sets: NR  Duration: 6m | *Tailoring:* NR  *Modifications:* NR | Ad*herence:* NR |
| Majed  (2020) | **UL EX + ED**  (Usual care) | *Materials:* The intervention group received pre-surgery ED and training on therapeutic EX. ED consisted of a PowerPoint presentation  regarding the therapeutic EX, information about the surgery, and a booklet with pictures of the EX to take home.  *Procedures:*  IG: The EX included deep breathing as well as shoulder EX such as extension of the triceps, bicep curl, paddling in sitting position, fluttering with both arms, hands behind neck, forward wall crawls, and side wall crawls + ED  CG: received routine hospital care that did not include any EX training or ED. Routine hospital care included explanation by the surgeon on the surgical procedure with follow-up at two and four weeks after discharge | ED: one-to-one sessions  EX: Demonstration of the EX by the PI was done to ensure proper techniques. The researcher called the patients at home every week to ensure that the women were continuing the EX + HB | *Location:* HB  Initiation: prior to surgery  Freq: NR  Int:NR  REPS/Sets: 10/NR  Duration: 4w | *Tailoring:* NR  *Modifications:* NR | Ad*herence:* NR  *Dropouts:*  IG: 5/35; refused to do the EX  CG: 4/34; lost to follow-up |
| de Almeida Rizzi  (2020) | **UL EX**  (Free vs limited ROM) | *Materials:* NR  *Procedures:* The patients started physical therapy at the hospital the day following surgery after they had learned and performed six EX (1–6 of protocol) At PO15, two EX were included (7 and 8 of the protocol)  IG: was allowed to perform the protocol EX and ADL in free amplitude (i.e., at the limit of pain or the sensation of tightening of the scar)  CG: limited-range group (control-standard center protocol), which had ROM maintenance limited to 90° for 15 more days (i.e., until the POD 30), when free-range EX also were allowed | All evaluations and physiotherapeutic interventions  were performed by PTs with expertise in breast oncology. | *Location:* Mastology Outpatient Clinic  Initiation: 1^st^ pod  Freq:  Int: NR  REPS/Sets: NR  Duration: NR | *Tailoring:* NR  *Modifications:* NR | Ad*herence*: NR  *Dropouts:*  IG: 4/31 (lost to follow-up)  CG: 3/31 (reoperation, lost to follow-up) |
| de Oliveira  (2014) | **EX +ED**  (MLD+ED) | *Materials: ED:* All participants were provided with information leaflets about proper care for the OA to prevent trauma, infection, and to avoid activities that involved to much load and/or repetition  *Procedures: MLD:* (1) ganglion evacuation in the axillary region ipsilateral to Sx and over ipsilateral inguinal region; (2) reabsorption, performed by gentle pressure and slow rhythmic mvts; *EX:* Nineteen EX were performed per sessions: 30% stretching of the scalene, trapezius, levator scapulae, pectoralis major and minor and rotator cuff muscles; 60% of AROM of the shoulder joint, followed by stretching of the deltoid, latissimus dorsi, rhomboids and pectoralis muscles and; 10% of relaxation EX | *MLD:* Three experienced PTs  *Lectures:* Multidisciplinary team of psychologists, nurses, social service workers and dietitians  *EX:* SPV by the physical therapy team | Location: Women’s Hospital Integral Healthcare Center-CAISM/UNICAMP  Initiation: 3^rd^ post-op day  Freq: 2 days/w  Int: NR  Duration: 1m | *Tailoring:* NR  *Modifications*: NR | *Adherence*: NR  *Dropouts:*  *EX group* 2 (4.17); *MLD group* 5 (10.4) |
| Huo  (2021) | **Personalized EX program**  (Usual care) | *Materials:* Health education brochures; the rehabilitation training did not require a venue or any equipment  *Procedures:*  IG: Routine nursing care + personalized rehabilitation EX intervention: (I) early rehabilitation EX (fist, wrist, forearm, elbow, elbow holding, upper arm, neck, body extension and shoulder lifts); (II) mid-term rehabilitation EX (stretching, pushing, pulling, shaking hands, chest expansions, side lifting, lifting, encircling, abdominal and back, body rotation and finishing EX; (III) late rehabilitation EX (warm-up, head shaking, head-up, arm extension, waist, waist turning circle and finishing EX); CG: received routine nursing intervention, including condition observation, ward environment maintenance, medication and diet guidance and underwent routine rehabilitation training which included fisting (hold the ball for 5s and release 5s, repeat 20 times), wrist (flexion and extension) and lifting EX | Providers: NR  Before hospital discharge: SPV  After hospital discharge: Mixed (SPV or HB) | Location: Rehabilitation center room of the hospital or HB after hospital discharge  *P1:*  Initiation: 24h after Sx  Freq: 10 sessions (Pod 1,2, 3, 5, 7, 9, 10, 11, 12 and 14); 3 /day  Duration: 2w  *P2:*  Initiation: 4w after Sx  Freq: 10 sessions; 3/day  Duration: 8w  *P3:*  Initiation: 12w after Sx  Freq: 8 sessions; 3 /day  Duration: up to 6m post-Sx | *Tailoring:*  EX were chosen based on the time that had elapsed  since the BSx  *Modifications:* NR | NR |
| Na  (1999) | **EX + PM**  (Usual care) | *Materials:* Printed material about the planned self-EX program and proper positioning  *Procedures:*  *Rehabilitation group:* 1^st^ pod: Assisted ROM EX of the shoulder, elbow, wrist and hands combined with active use of the involved arm for light functional activities; Patients also received passive pain-relief modalities (PENS, heat therapy, cold therapy) and therapeutic EX on the 3^rd^ pod; Patients were encouraged to elevate the OA on a pillow above the heart level while sleeping and as often as possible during the day; Isometric pumping EX of distal muscles were also included.  *Control group:* Were provided with no rehabilitative treatment but with instruction alone for ROM EX and postural EX | Providers: unclear  SPV + Self-A | Location: Hospital setting +HB upon hospital discharge  Initiation: 1^st^ pod  Freq: 4 times/day  Int: NR  Program duration: 40 minutes of PT and 30 minutes of EX  Duration: 4w | *Tailoring:* Patients with LE received intermittent pneumatic compression  *Modifications*: NR | *Adherence*: NR  *Dropouts:* NR |
| Oliveira (2018) | **EX +ED**  **(MLD+ED)** | *Materials: ED:* All participants were provided with information leaflets about proper care for the OA to prevent trauma, infection, and to avoid activities that involved to much load and/or repetition  *Procedures: MLD:* (1) ganglion evacuation in the axillary region ipsilateral to Sx and over ipsilateral inguinal region; (2) reabsorption, performed by gentle pressure and slow rhythmic movements  *EX:* Nineteen EX were performed per sessions: 30% stretching of the scalene, trapezius, levator scapulae, pectoralis major and minor and rotator cuff muscles; 60% of AROM of the shoulder joint, followed by stretching of the deltoid, latissimus dorsi, rhomboids and pectoralis muscles and; 10% of relaxation EX | *MLD:* Three experienced PTs  *Lectures:* Multidisciplinary team of psychologists, nurses, social service workers and dietitians  *EX:* SPV by the physical therapy team | Location: Hospital setting +HB upon hospital discharge  Initiation: 1^st^ pod  Initiation: 3^rd^ post-op day  Freq: 2 times/w  Int: NR  Duration: 1m | *Tailoring:* NR  *Modifications*: NR | *Adherence*: NR  *Dropouts: EX group:* 2m: 2(3.44); 30m: 47 (81.03)/ MLD group: 2m: 4(6.90); 30m: 23 (39.66) |
| Tirolli Rett  (2022) | **PT protocol** | *Materials:* Elastic bands and dumbells of 0.5 to 1.0 kg; all participants received written guidance regarding limb and skin care  *Procedures:* Consultation involved passive GH and scapulothoracic mobs, cicatricial mobs, passive stretching of the cervical musculature and UL, pendulum EX, and shoulder AROM EX | Providers: PTs  SPV and individualized consultations | Location: PT sector of OncoRadium, municipality of Aracaju, Sergipe  Initiation: Between 4-8w after Sx  Freq: 3 times/w  Sets/Reps: 3/8-12  Consultation duration: 60 min  Duration : 20 sessions, 7w | *Tailoring:* NR  *Modifications:* NR | *Adherence:* NR  *Dropouts:* Of the 65 women selected, 49 completed the study (dropped out n=12; death n=2; did not answer the questionnaires n =2) |
| Kim  (2019) | **EX**  (Delayed EX) | *Materials:* NR  *Procedures: Rehabilitation protocol:* Short term immobilization period (2w) followed by a self-EX program that consisted of 6 types of progressive shoulder stretch EX, including beach pose, chest stretch, and biceps curl with low weight; *Conventional protocol:* Patients were asked to immobilize the OA for more than 4w and engaged themselves in the same self-EX program after the immobilization period | Providers: unclear  EX: self-A  PT: supervised | Location: HB  Initiation: *Rehabilitation group:* 3rd post-op w; *Conventional protocol group:* 5^th^ post-op w  Freq: 4 times/day; 7 days/w  Int: NR  Sets/REPS: 1/5-10  Duration: Unclear | *Tailoring:* In patients diagnosed with adhesive capsulitis, supervised shoulder physical therapy was performed  *Modifications*: NR | *Adherence*: NR  *Dropouts:* NR |
| Lu  (2015) | **PT + ED**  **ED**  (Usual care) | *Materials:* Printed instructions were provided  *Procedures: Group A:* Received neither education nor PT post Sx; *Group B:* Received an educational program on LE (information on the lymphatic system, symptoms and signs of LE and suggestions for preventing LE) between PO0 and PO7; *Group C:* PT program which included breathing EX, postsurgical positioning, massaging of scar tissue and stretching of soft tissue, shoulders mobs and shoulder and UE EX | Supervised by PTs during the first post-op w and continued at outpatient clinics post-discharge | Location: Hospital-based + outpatient clinics  Initiation: 1^st^ post-op w  Freq: 2 times/w  Int: NR  PT sessions duration: 30 min  Duration: Unclear | *Tailoring:* The total number of PT sessions varied according to the clinical condition of each patient  *Modifications*: NR | *Adherence*: NR  *Dropouts:* NR |
| Manfuku  (2021) | **BME +PT**  (PNE + PT) | *Materials: PT:* Pamphlet which included illustrations of shoulder joint EX and instructions on how to prevent LE (skin care, precautions on daily life, precautions on activity and overuse, prevention of infections and injuries); *BME and PNE:* Was explained face-to-face using a pamphlet with pictures and illustrations  *Procedures: PT*: comprised passive mobs to restore shoulder ROM, stretching to improve flexibility of the scar, and EX for muscular strength  *BME:* Educational sessions on the anatomy of the breast, surgical procedure for BC, mechanisms underlying postop pain; *PNE:* included information on the characteristics of acute and chronic pain, pain-related side effects of BC treatment modalities, how pain is processed in the brain, how pain persists and on potential pain-sustaining factors | Providers: All interventions and assessments were provided by PTs who where experienced in the management of patients with cancer pain  SPV individual sessions | Location: Outpatient clinic  Initiation: *BME or PNE*: 1w before surgery  *PT:* 1w after surgery  Freq: once/1-2w  Int: Low to moderate (increased over the course of treatment)  Program duration: 40 min  Duration: 3m | *Tailoring:* EX intensity progressively increased with participants gaining competence both in the clinic and at home  *Modifications :* NR | *Adherence:* NR  *Dropouts:* BME group: 6 (7.7); PNE group: 4 (5.3); no reasons were provided |
| Morimoto (2003) | **EX** | *Materials:* A leaflet and a video were used to explain the contents of the program and its applications  *Procedures: Postop day 1:*  Prevention of development of rigidity of shoulder joint on the OA: Lateral and forward arm raising on the affected side in the dorsal sitting positions; *Postop day 2:* Training for force releasing through EX of shoulder joint: Backward arm raising, finger crawling on a wall and force releasing/concentrating by keeping the arm on affected side at a position of backward raising at 90°; *Postop day 3:*EX to approximate preoperative life: wing flapping and touching the ears and back with the hands; *Postop day 4:* EX to reduce functional differences between the normal and affected sides: Reduction of right and left side differences through the above-mentioned EX on postop days 1–3 | Evaluations were carried by the nurse in charge  Self-A | Location: HB  Initiation: 1^st^ pod  Freq: 3-4/day, daily  Int: NR  Duration: After hospital discharge (postop day 10-14) was entrusted to the patient’s own initiative | *Tailoring:* NR  *Modifications*: NR | *Adherence*: NR  *Dropouts:* NR |
| Paolucci  (2021) | **Rehabilitative treatment (EX)** | *Materials:* For greater protection, patients were given an illustrative pamphlet with the EX to continue to perform at home  A small cylinder was used for postural re-alignment EX and rubber bands for strengthening EX + Rehabilitation diary  *Procedures*: *P1:* Relaxation and diaphragmatic breathing EX in the supine position, EX of postural re-alignment, decoaptation and co-activation of the GH joint, stretching of the pectoral muscles and subscapularis muscle, mobs of the scapulothoracic joint, cervical pumping, AROM, isometric strenghtening EX in flexion, abd, and add, Codman’s pendulum, balance EX  *P2:* EX to be performed at home + indication to walk at least 30 min every day; AROM EX, twisting of the trunk in sitting and standing position, active cervical mobs | Providers (*P1*): PTs  Mixed  *P1*: SPV  *P2*: self-A | Location: *P1:* Rehabilitation outpatient clinic of University Hospital Umberto I; *P2:* HB  Initiation: unclear  *P1:*  Freq: 2 times/w  Sets/REP: 3/10  Rehabilitation sessions duration: 1h  Duration: 5w + 2m at home  *P2:*  Freq: at least 2 times/w | *Tailoring:* The PT tailored the rehabilitation to patients’ functional problems  *Modifications:* NR | *Adherence:* NR  *Dropouts:* 3 (7.89) for personal problems |
| Scaffidi  (2012) | **EX + PT + ED**  (EX+ED) | *Materials:* Educational information was given in written form to group B, in addition with an exhaustive brochure containing pictures and explanations of the EX  *Procedures: Both groups:* Received preoperative verbal information regarding postoperative side effects and on precautions to observe immediately after Sx and afterwards in everyday life; Were provided with a home-based rehabilitation program which consisted of EX to maintain flexibility and elasticity of the muscles surrounding the shoulder joint; *Group B:* PT sessions that were initially focused on deep breathing EX, stretching neck muscles, elevation, abd, external and internal rotation of shoulder, flexion and extension of elbow in neutral position | Providers: Dedicated PTs were in charge of PT sessions from the day of Sx until the hospital discharge  Mixed: SPV + self-A | Location: S. Andrea Hospital, Rome + HB  Initiation: Preoperative period  Freq: *PT (hospital-based*): 1/day*; EX program*: 3 times/day  Int: NR  Duration: unclear | *Tailoring:* In the presence of articular and/or functional limitations and/or LE at follow-up visits, the most appropriate PT program was prescribed  *Modifications*: NR | *Adherence*: Patient compliance to the home rehabilitation program was judged ˝really high˝ but was not detailed in the article  *Dropouts:* NR |
| Springer  (2010) | **EX + ED** | *Materials:* NR  *Procedures: S*ubjects were instructed in a postop UL ROM EX program, and were educated regarding UL LE precautions and physical EX initiation and progression | Baseline and follow-up evaluations were conducted by a PT  Self-A | Location: HB  Initiation: *ED:* Preoperative period; *EX:* After Sx  Freq: NR  Int: NR  Duration: Unclear | *Tailoring:* At 1-month postop visit, the EX program was reviewed and additional individualized home program instructions were provided as needed.  *Modifications*: NR | *Adherence*: NR  *Dropouts:* NR |
| Hsieh  (2008) | **EX** | *Materials:* NR  *Procedures:* 10-min warm-up, 40 min of aerobic EX (outdoor or treadmill walking, stationary cycling, recumbent stepping opr walking on a underwater treadmill), resistance training and stretching and 10-min cooldown | Providers: Trained cancer-certified cancer EX specialists  SPV | Location: Outpatient oncology rehabilitation center  Initiation: immediately following treatment for BC (unclear)  Freq: 2-3 days/w  Int: 40-65% of HR reserve (based on the treadmill assessment results)  EX sessions duration : 1h  Duration: 6m | *Tailoring:* EX prescriptions and interventions were individualized based on the results of the medical and cancer history, physical examination and the initial psychological and physiologic assessments  *Modifications*: NR | *Adherence*: Follow-up examinations revealed that participants’  adherence to the EX intervention was approximately  90%,  *Dropouts:* NR |
| Petito  (2012) | **EX** | *Materials:* NR  *Procedures: Phase 1 (1^st^ postop day until drain removal):*  Two stretches for the cervical region, two EX for movement of the scapular girdle, one for shoulder flexion and one for extension beyond the midline  *Phase 2 (after drain removal until the 105th postop day:* Three additional EX: one EX for flexion and two for abd of the shoulder. | Providers: ˝Instructor˝ (unclear)  Self -A | Location: HB  Initiation: 1^st^ pod  Freq: Daily  Int: NR  Rep: 10/ exercise  Duration: 105 post-op days | *Tailoring:* NR  *Modifications*: NR | *Adherence*: Satisfactory adherence from the 7th PO until the 75th PO varied from 75.8 to 91.2%. Adherence declined according to the recuperation of the ROM.  *Dropouts:* N=22 did not attend the penultimate or last evaluation, n=5 required reoperation. |
| Singh  (2013) | **PT + ED**  (ED) | *Materials:* All participants were given the same printed educational material, which included information on postop EX and activity modification, scar massage and prevention of secondary LE  *Procedures: Both groups:* Standardized ED, including AROM EX, information about LE, and information about scar massage along the line of the incision after healing; Once the drain was removed (>1w post-op), participants were asked to begin supine active assisted shoulder flexion and extension and wall-walking EX and gentle seated pectoral stretch; *Exp group:* Were provided with PT treatments which focused on teaching self-management strategies, LE management, scar tissue massage and progressive active and assisted shoulder EX | Providers: Trained PTs  Mixed: SPV + self-A | Location: unclear + HB  Initiation: Preoperative period (ED) and 1^st^ post-op w (EX and PT)  Freq: unclear  Int: NR  Duration: 12m | *Tailoring:* Further PT treatments were provided if indicated  *Modifications*: NR | *Adherence*: NR  *Dropouts:* One participant  did not complete the 7-month follow-up visit |
| Rekha  (2020) | **EX**  (EX) | *Materials:* NR  *Procedures: Int group:* was treated with Swiss ball EX (roll away, roll on, hold on, hold up and cat press) and diaphragmatic breathing EX; *Control group:* Was treated with stretching (butterfly stretch, blade squeeze, shoulder stretch, corner stretch and chest stretch) and diaphragmatic breathing EX | Providers: PTs  SPV | Location: Saveetha Medical College Hospital  Initiation: within one-month period following Sx  Freq: 5 days/w  Int: NR  Duration: 4w | *Tailoring:* NR  *Modifications*: NR | *Adherence*: NR  *Dropouts:* NR |
| Kilgour (2008) | **EX**  (Usual care) | *Materials:* HBE group received a home EX video that included the Ex and guidelines found in the CCS brochure  *Procedures: P1:* ROM and flexibility EX of the neck and shoulder regions (Codman pendulum arm swing, canoe stroke, wall walk, ball squeezing)  *P2:* Same EX but longer sets | Self-A | Location: HB  Initiation: 3^rd^ pod; P1 (1^st^ pod to PO9); P2 (PO10 to PO14)  Freq: 2-3 sets a day  Int: NR  Sets duration: 5-15 minutes  Duration: 11 days | *Tailoring:* NR  *Modifications*: NR | *Adherence*: HBE group: *P1:* 80% completed at least 1 set, 2 sets (76%) or all three sets (59%); *P2:* 50% of women completed 1 and 2 sets  *Dropouts:* NR |
| Baima  (2017) | **EX**  (EX) | *Materials:* Participants in group A received an information sheet with the EX and a video link and those in group B received an instruction sheet with optional video  *Procedures:* (1) Codman’s exercise; (2) Scapular squeezes; (3) Reach for the pillow  Both groups were advised to restrict any upper arm EX above 90° of abduction while drains were in. | Group 1: a member of the research team demonstrated each of the three EX  Group 2: self-A | Location: HB  Initiation: 1-4w prior to Sx  Freq: Once daily, suspended post Sx  Sets/REPS: 3/10  Int: NR  Duration: unclear | *Tailoring:* NR  *Modifications*: NR | *Adherence*: 34 out of 45 subjects (75% after in-person teaching; 77% after video-teaching) chose to EX  *Dropouts:* NR |

***Abd:*** *abduction;* ***ACSM:*** *American Health Association for Advanced Cardiac Life Support;* ***Add:*** *adduction;* ***ADL****: Activities of daily living;* ***AR****: Activity restriction;* ***AROM****: Active range of motion;* ***BC****: Breast cancer;* ***BME:*** *Biomedical education;* ***BSx:*** *Breast surgery;* ***CCS:*** *Canadian Cancer Society;* ***Ch:*** *Chemotherapy;* ***CG****: Control group;* ***CRC:*** *Clinical Research Center;* ***DG:*** *Delayed group* ***ED****: Education;* ***EG*** *: Early group;* ***EX****: Exercise(s);* ***Freq:*** *Frequency;* ***FROM****: Free range of motion;* ***GH:*** *Gleno-humeral;* ***HB****: Home-based;* ***IG****: Intervention group;* ***Int:*** *Intensity;* ***KBR:*** *Kinect based rehabilitation;* ***LD:*** *Lymphatic drainage;* ***LE****: Lymphedema;* ***LPM****: Lymphedema Prevention Module;* ***m:*** *months;* ***min****: minutes;* ***MLD****: Manual lymphatic drainage;* ***Mobs:*** *mobilizations;* ***MT:*** *Manual therapy;* ***Mvts****: Movements;* ***NAR:*** *No activity restriction;* ***NR:*** *Non reported;* ***OT****: Occupational therapist;* ***PENS:*** *Percutaneous electrical nerve stimulation****; pod****: postoperative day;* ***PNE:*** *Pain neuroscience education;* ***Preop****: Preoperative;* ***PROM****: Passive range of motion;* ***PT****: Physical therapy(ist)****; REP:*** *Repetitions;* ***RM:*** *Repetition maximum;* ***ROM:*** *Range of motion;* ***RT:*** *Radiotherapy;* ***Self-A :*** *Self-administered;* ***SLD:*** *Simple lymphatic drainage;* ***SMLP:*** *Self-management of lymphedema program;* ***SPV****: Supervised;* ***ST****: Soft tissue;* ***Sx****: Surgery;* ***t_x_****: Treatment(s);* ***UC:*** *Usual care;* ***UE:*** *Upper extremity;* ***UL:*** *Upper limb;* ***w****: week;*
